# Multi-model Meteorological and Aeolian Predictions for Mars 2020 and the Jezero Crater Region

**DOI:** 10.1007/s11214-020-00788-2

**Published:** 2021-02-08

**Authors:** C. E. Newman, M. de la Torre Juárez, J. Pla-García, R. J. Wilson, S. R. Lewis, L. Neary, M. A. Kahre, F. Forget, A. Spiga, M. I. Richardson, F. Daerden, T. Bertrand, D. Viúdez-Moreiras, R. Sullivan, A. Sánchez-Lavega, B. Chide, J. A. Rodriguez-Manfredi

**Affiliations:** 1 Aeolis Research, Tucson, AZ USA; 2 Jet Propulsion Laboratory, California Institute of Technology, Pasadena, CA 91001 USA; 3 Centro de Astrobiología (CSIC-INTA), 28850 Madrid, Spain; 4 Space Science Institute, Boulder, CO 80301 USA; 5 Ames Research Center, Mountain View, CA USA; 6 The Open University, Milton Keynes, UK; 7 Belgian Institute for Space Aeronomy, Brussels, Belgium; 8 Laboratoire de Météorologie Dynamique/Institut Pierre Simon Laplace (LMD/IPSL), Sorbonne Université, Centre National de la Recherche Scientifique (CNRS), École Polytechnique, École Normale Supérieure (ENS), 75005 Paris, France; 9 Institut Universitaire de France, 75005 Paris, France; 10 LESIA, Observatoire de Paris, Université PSL, CNRS, Sorbonne Université, Université de Paris, 92195 Meudon, France; 11 Cornell Center for Astrophysics and Planetary Science, Cornell University, Ithaca, NY 14853 USA; 12 Universidad del País Vasco (UPV/EHU), Bilbao, Spain; 13 Institut Supérieur de l’Aéronautique et de l’Espace (ISAE), Toulouse, France

**Keywords:** Mars, Meteorology, Aeolian, Atmosphere, Dust devils, Mars 2020, Jezero crater

## Abstract

Nine simulations are used to predict the meteorology and aeolian activity of the Mars 2020 landing site region. Predicted seasonal variations of pressure and surface and atmospheric temperature generally agree. Minimum and maximum pressure is predicted at $\text{Ls}\sim 145^{\circ}$ and $250^{\circ}$, respectively. Maximum and minimum surface and atmospheric temperature are predicted at $\text{Ls}\sim 180^{\circ}$ and $270^{\circ}$, respectively; i.e., are warmest at northern fall equinox not summer solstice. Daily pressure cycles vary more between simulations, possibly due to differences in atmospheric dust distributions. Jezero crater sits inside and close to the NW rim of the huge Isidis basin, whose daytime upslope (∼east-southeasterly) and nighttime downslope (∼northwesterly) winds are predicted to dominate except around summer solstice, when the global circulation produces more southerly wind directions. Wind predictions vary hugely, with annual maximum speeds varying from 11 to $19~\text{ms}^{-1}$ and daily mean wind speeds peaking in the first half of summer for most simulations but in the second half of the year for two. Most simulations predict net annual sand transport toward the WNW, which is generally consistent with aeolian observations, and peak sand fluxes in the first half of summer, with the weakest fluxes around winter solstice due to opposition between the global circulation and daytime upslope winds. However, one simulation predicts transport toward the NW, while another predicts fluxes peaking later and transport toward the WSW. Vortex activity is predicted to peak in summer and dip around winter solstice, and to be greater than at InSight and much greater than in Gale crater.

**Supplementary Information:**

The online version contains supplementary material available at 10.1007/s11214-020-00788-2.

## Introduction

The Mars 2020 Perseverance rover and accompanying Ingenuity Helicopter will land on Mars on 18 February 2021. On Mars, the areocentric solar longitude, Ls, will be $\sim 5^{\circ}$ at this time, corresponding to local (northern hemisphere) spring of Mars Year (MY) 36. The center of the landing ellipse has longitude $77.43~^{\circ}\text{E}$, latitude $18.47~^{\circ}\text{N}$, and elevation $-2.55~\text{km}$. This puts the landing site on the northwestern slopes of Jezero crater, a $\sim49~\text{km}$ diameter impact crater sitting on the northwestern slopes of the $\sim1500~\text{km}$ diameter Isidis basin, on the global-scale topographic dichotomy boundary. This provides an opportunity to measure both the near-surface atmospheric circulation and the aeolian activity due to that circulation at an interesting new location on the Martian surface, one at which the circulation is influenced by both regional (dichotomy boundary and Isidis basin) and local (Jezero crater) topography. Over the course of the mission the rover may drive a significant distance, allowing it to quantify the changing meteorology and circulation – and how those changes affect aeolian activity – across a wider region.

The set of instruments carried by Mars 2020, described in Sect. 2 and in other papers in this special issue, are able to monitor a wide range of variables that are relevant to meteorological and aeolian studies. They will measure both meteorological variables (pressure, temperature, winds, and water vapor) and the forcing that drives their behavior over time (e.g. aerosol abundances and properties, and radiation fluxes at the surface). These measurements will enable investigations of processes operating on timescales from seconds to years, ranging from understanding the statistics of convective vortices and atmospheric turbulence to determining the impact of local topography or atmospheric dust opacity on near-surface wind stress, sand motion, and dust lifting. Images of the surface, combined with measurements of the local circulation, will provide insight into aeolian features such as ripples, dunes, ventifacts, and dusty convective vortex (dust devil) tracks. Changes detected on the surface or rover deck will be correlated with winds and vortex activity to shed light on aeolian processes and the threshold conditions that must be exceeded for dust lifting or active saltation.

The model predictions and intercomparisons presented here are intended to provide context for such meteorological and aeolian studies, but also provide useful information for science teams and engineers working to refine mission operations. In addition, Objective D3 of the Mars 2020 mission is to make surface weather observations to validate global atmospheric models (Farley et al. 2020, this journal). As noted in the 2020 Mars Exploration Program Analysis Group (MEPAG) Goals Document (Banfield 2020): “Numerical modeling of the atmosphere is critical to understanding atmospheric and climate processes. Models provide dimensional and temporal context to necessarily sparse and disparate observational datasets […] and constitute a virtual laboratory for testing whether observed or inferred conditions are consistent with proposed processes.” The seven models used in this study (with output at two different horizontal resolutions presented for two of them, making nine simulations in total) represent a significant fraction of the Mars atmospheric models in current use. The results obtained during this intercomparison exercise should therefore enable a rapid assessment of how well current Mars models are able to predict the near-surface atmospheric state at a new location on Mars, and pave the way for more detailed investigations of model-data discrepancies and their causes.

The near-surface atmosphere is where many key processes occur that control the entire atmospheric circulation, from surface exchange of heat, momentum, and trace gases (including water vapor) to surface heating that drives daytime planetary boundary layer (PBL) convection (e.g. Stull 1988). The greatest source of climate variability on Mars is associated with major dust storms, due to the large impact of dust loading on the thermal state of the thin Martian atmosphere (e.g. Gierasch and Goody 1972; Wolff et al. 2017). Yet we still do not understand what controls the onset, evolution, and decay of such storms, in particular the processes involved in dust lifting by wind stress, which likely drives onset of such storms (Newman et al. 2002a,b; Kok 2010; Newman and Richardson 2015; Musiolik et al. 2018; Swann et al. 2020). Our knowledge of near-surface meteorology is mostly gleaned from the few surface missions to date, which have largely provided bulk variables (temperature, pressure, horizontal wind, etc.) at a single height above the surface (e.g. Martínez et al. 2017). Unfortunately, real understanding of PBL and surface processes on Mars – such as the vertical mixing of heat and momentum and the exchange of both with the surface – require the measurement of variables at multiple heights and/or fluxes of heat, momentum, etc. (Rafkin et al. 2009; Newman et al. 2020). For Earth atmospheric models, parameterizations of these processes are based on numerous rigorous field observations. For Mars, parameterizations are typically adapted from those developed for Earth, with the choice of scheme and its parameters then determined by comparing their predictions of bulk variables with Mars observations. Schemes have also been developed by using Mars Large Eddy Simulations (LES) to provide simulated PBL measurements (e.g. Temel et al. 2021), but the problem remains of how to validate the LES results themselves. Overall, a wide range of schemes and parameter values are used in Mars models, in large part due to this lack of information on what the correct choices are. Vertical grid spacings, especially near the surface where profiles of temperature and wind often change rapidly with height, also vary widely between models, as does the specific 4-D distribution of atmospheric dust, choice of roughness length and surface thermal properties, and even how variables are extrapolated from model layers to a given height above the surface (see Sect. 3.8). As a result, and as shown in this study, models typically differ widely in their predictions of the near-surface atmosphere. Differences in the predicted near-surface wind directions and wind stresses will have a large impact on predicted sand (and dust) fluxes, predicted sand transport directions, and hence predicted aeolian activity and features, including dust storms. While more comprehensive measurements are desirable, near-surface measurements of bulk variables at one height do provide a crucial means of determining which models are performing best, from which we can try to understand why and learn how to improve models in general.

Combining aeolian and meteorological measurements will allow us to better understand the physics of aeolian processes in the Jezero region, and on Mars in general. Obtaining an estimate of the threshold wind stress required for saltation will help to explain currently active aeolian features all across Mars and the physical processes involved in triggering dust storms. It will also shed light on the potential past climates needed to explain the aeolian record, as preserved by the characteristics of depositional bedforms, erosional features, and some sedimentary rocks (Mars 2020 Objective A). Past saltation may have affected the distribution of micro-organisms in the near-surface, potentially by destroying radiation- or oxygen-resistant organisms via saltation-mediated abrasion (Bak et al. 2019). Present day wind and aeolian measurements should enable a clearer understanding of the age of surface aeolian features, which may then be relevant to the search for materials with high biosignature preservation potential (Mars 2020 Objective B.2). Environmental measurements will also help to constrain the weathering and preservation potential of a possible cache sample (Mars 2020 Objective C.1). Linking the occurrence of strong wind stresses and/or convective vortices to observed changes in dust cover will help us understand how dust is removed from the surface of Mars. Understanding dust lifting processes is a fundamental requirement for being able to correctly simulate – and perhaps one day predict – major dust storms. Because dust is the largest driver of Martian weather and climate variability (e.g. Read et al. 2015; Forget and Montabone 2017; Martínez et al. 2017), this has great importance for predicting atmospheric conditions that may be encountered by future robotic and manned missions (Objective D), especially at critical times such as during Aerobraking and EDL. In addition, better understanding of dust storms, dust lifting, sand motion, and the particle sizes and fluxes involved is important to both robotic and human surface operations, with the impact of dust on health especially important in the latter case.

In this work, we present predictions of the meteorology and aeolian activity at the Mars 2020 landing site in Jezero crater from nine different simulations performed using seven Mars atmospheric models. Four of the simulations are run at high spatial resolution, between $\sim1.4$ and 10 km horizontal grid spacing, while the remaining five are run at relatively low spatial resolution, between $\sim120$ and 300 km. We focus on the diurnal cycles of pressure, temperature (air and surface), and wind (speed and direction) at the landing site at the time of landing, areocentric solar longitude $\text{Ls}\sim5^{\circ}$, which corresponds to early northern spring, placing these results into context by examining the regional circulation. We also examine the expected seasonal variation in pressure, temperature, and circulation at the landing site, again placing them in regional context. We further provide aeolian predictions for the landing site from eight simulations, and for the wider Jezero crater region for the two highest-resolution simulations shown in this paper, mesoscale MarsWRF and MRAMS (see Table 1), comparing with aeolian features observed from orbit. We highlight the differences in model predictions, explaining them where possible, and demonstrate the value of Mars 2020 data for increasing our understanding of and predictive skill for the Martian near-surface circulation and aeolian activity. A companion paper, Pla-García et al. (2020, this journal), examines the detailed meteorology expected at different seasons in more detail, including the expected water cycle, again using results from the two highest-resolution model simulations shown here.

**Table 1:** Horizontal grid spacing, surface properties at the landing site, height of the midpoint of the lowest model layer, and height to which atmospheric temperature ($T_{\mathit{air}}$) is extrapolated, for the nine simulations presented here. See above and Sects. 3.1–3.7 for more explanation

Simulation	Horizontal grid spacing	Albedo	Thermal inertia ($\text{J}\,\text{m}^{-2}\,\text{K}^{-1}\,\text{s}^{-1/2}$)	Emissivity	Roughness map/method	Roughness height, $z_{0}$ (m)	Height of lowest model layer (m)	$T_{air}$ given at (m)
GEM-Mars	4^∘^ (237 km)	0.1935	288.5	0.950	Hébrard	0.0074	2	2
LMD (global)	5^∘^ (296 km)	0.186	283.0	0.950	Hébrard	0.0078	4.5	4.5
OpenMARS	5^∘^ (296 km)	0.1667	321.0	0.950	Hébrard	0.0110	5	1.5
Ames (low resolution)	1.875^∘^ (111 km)	0.1540	284.4	0.944	Uniform	0.0100	5	5
MarsWRF (global)	2^∘^ (118 km)	0.1342	260.7	0.950	Garvin	0.0267	10	1.5
LMD (mesoscale)	10 km	0.165	319.0	0.950	Hébrard	0.0110	4.5	4.5
Ames (high resolution)	6.6 km	0.1721	293.4	0.941	Uniform	0.0100	5	5
MarsWRF (domain 5)	1.4 km	0.1342	260.7	0.950	Garvin	0.0267	10	1.5
MRAMS	2.96 km	0.1362	260.0	0.950	Garvin	0.0300	14.5	14.5

Section 2 briefly describes the Mars 2020 instruments and measurements that will provide meteorological and aeolian information that may be compared with the results of this study. Section 3 describes the seven atmospheric models and how they were set up to produce the nine simulations used for this intercomparison. Section 4 describes how meteorological observations or model predictions may be combined with aeolian theory and assumed surface properties to predict aeolian features and activity. Section 5 presents multi-model meteorological predictions for the Mars 2020 landing site at the landing date of $\text{Ls}\sim5^{\circ}$, while Sect. 6 presents predictions for the landing site as a function of season. Section 7 uses output from two high-resolution simulations to predict the changes in circulation that may occur if the rover drives out of Jezero crater toward the Midway and NE Syrtis locations. Section 8 predicts sand fluxes and bedform migration rates and orientations at the landing site and across the Jezero region. Section 9 predicts dust devil activity in the Jezero region and compares with other landing sites. Section 10 compares aeolian predictions over the Jezero region with orbital observations of aeolian features and their motion. Finally, Sect. 11 summarizes results and concludes.

## Instruments and Measurements Relevant to Meteorological and Aeolian Processes

In this section we provide an overview of the Mars 2020 sensors that will be used to measure the meteorological variables and aeolian activity predicted in this paper. In addition to the measurements described below, Mars 2020 instruments will also measure water vapor relative humidity and column abundances, other trace gas abundances, and water ice properties. Those measurements are not described here as this paper does not include predictions of those quantities. That does not mean such quantities cannot be predicted by this set of models, rather that we chose to focus on the basic meteorological variables, circulation patterns, and aeolian activity. Indeed, five of the simulations (Ames x 2, GEM-Mars, OpenMARS, and global LMD; see Table 1) predicted the seasonal and diurnal variation of water vapor, but such predictions remain very sensitive to assumptions and parameters included in the models, thus were deliberately excluded. However, Pla-García et al. (2020, this journal) examine the expected variation of water vapor in Jezero crater guided by orbital observations, and a comparison of predicted water vapor abundance should form part of any future intercomparison study.

### The Mars Environmental Dynamics Analyzer (MEDA)

The primary meteorological instrument on Mars 2020 is the Mars Environmental Dynamics Analyzer (MEDA). In several respects, MEDA is similar to MSL’s Rover Environmental Monitoring Station (REMS) (Gómez-Elvira et al. 2014), but it has important additions and improvements (Rodriguez-Manfredi et al. 2020, this journal). Unlike REMS, which had very limited memory, MEDA will be able to measure a complete daily meteorological cycle at 1 Hz (or for some sensors, 2 Hz) frequency. The planned MEDA baseline is to measure at least 12 hours per sol at a frequency of 1 Hz (Rodriguez-Manfredi et al. 2020, this journal), although this may be limited by the power or data storage/downlink available.

### Temperature and Pressure

The MEDA pressure sensor (PS) sits inside the rover body, connected to a tube and HEPA filter with a geometry that is designed to be insensitive to wind velocity. The MEDA atmospheric temperature sensors (ATS) are mounted at several heights on the rover, including just below the two wind sensor booms at $\sim1.5~\text{m}$ on the Remote Sensing Mast (RSM) and on the sides of the rover body, at 0.5 m. The MEDA Thermal InfraRed Sensor (TIRS) will also provide an estimate of atmospheric temperature over a region centered at $\sim40~\text{m}$ altitude, as well as measuring surface brightness temperature about 3 m from the rover.

Pressure and air temperature can be used to estimate air density using the ideal gas equation, a vital requirement for estimating wind stress (see Sect. 2.6). These variables, in addition to wind speed and direction, also react strongly to the passage of clear or dusty convective vortices. Pressure data are particularly useful, due to the distinctive, rapid pressure drop and recovery as a vortex passes over or close to the sensor, hence pressure data have been commonly used to identify the statistics of vortex occurrence on Mars (e.g. Schofield et al. 1997; Ellehøj et al. 2010; Kahanpää et al. 2016; Steakley and Murphy 2016; Ordoñez-Exteberria et al. 2018; Newman et al. 2019a).

### Radiative Fluxes and Aerosols

Between them, TIRS and the Radiation and Dust Sensor (RDS) also included in MEDA will constrain solar and IR upward and downward fluxes, surface properties, and aerosol (dust and to a lesser extent water ice) abundances and radiative properties at the rover’s location (Rodriguez-Manfredi et al. 2020, this journal). In addition, the Mastcam-Z cameras mounted on the RSM (Bell et al. 2020), which are able to provide color images and video in any direction with a powerful zoom capability, will measure aerosol (dust and water ice) abundance, vertical distribution, size distribution, and optical properties, as done using Mastcam on MSL (Lemmon et al. 2019). The angular distribution of sky brightness observed by the Navigation (Navcam) and Hazard Avoidance (Hazcam) cameras may also be used to determine the aerosol abundances and properties, again as on MSL (Chen-Chen et al. 2019a,b). Finally, the SuperCam instrument (Wiens et al. 2020, this journal) will be used to obtain aerosol abundances and properties, as done using the ChemCam instrument on MSL (McConnochie et al. 2018). In combination, this information on radiative fluxes and aerosol abundances and properties will aid interpretation of the meteorological measurements and enable future atmospheric modeling to be performed using more realistic local surface properties and aerosol forcing.

### Wind

Like REMS, MEDA has two wind sensor booms mounted on the RSM at $\sim1.6~\text{m}$ altitude, which point at $120^{\circ}$ to each other in the horizontal plane (see Fig. 29 of Rodriguez-Manfredi et al. 2020, this journal). This is required to measure winds correctly from all directions because the RSM strongly perturbs wind that arrives at it before the sensor. Unfortunately, major damage to the wind sensors on the side-/rear-pointing REMS boom on landing produced large gaps and biases in the wind dataset (Gómez-Elvira et al. 2014; Newman et al. 2017; Viúdez-Moreiras et al. 2019a,b). Further damage to the wind sensors on the front-pointing boom on MSL $\text{sol}\sim470$ ($\sim2.2$ Mars years into the MSL mission) meant that no REMS wind data have been available since September 2016. In addition, the front-pointing wind sensor boom suffered major electronic noise for temperatures below $\sim210~\text{K}$, which meant that wind measurements could not be obtained for between 6 and 10 hours overnight, depending on season.

The design of the MEDA booms addresses many of the above issues. Where REMS has three wind sensor boards arranged around each boom, MEDA has six, providing increased redundancy. MEDA’s side-/rear-pointing boom is also longer than on REMS and unfolds post-landing, which both provides better damage protection and allows wind measurements to be made further away from flow interruptions caused by the RSM. In addition, as already done for InSight’s wind sensors (Velasco and Rodríguez-Manfredi 2015), the design of the electronics has been improved, enabling calibration of the wind sensors for wind speeds of up to 40 m/s (versus $\sim20~\text{m/s}$ for REMS) and reducing noise (Rodriguez-Manfredi et al. 2020, this journal). Mars 2020 also carries a microphone on SuperCAM that may be able to determine wind speed from the intensity of the noise at low frequencies ($<500~\text{Hz}$) and wind direction from the relative intensity of the noise measured at different rover pointings (Chide et al. 2021). These measurements will be used for cross-calibration with MEDA’s wind sensors where possible. Finally, the Ingenuity Helicopter’s flight control sensors include measurements of air pressure and vehicle acceleration that should provide insight into the winds that it encounters at various levels during flight. This may provide an idea of the vertical wind profile near the surface of Mars, and hence an alternative estimate of wind stress using the flux profile method described in Sect. 2.6.

Convective vortices (either clear or dusty) are formed of rotating air around a central, low-pressure vortex core. They therefore produce a perturbation, in both wind speed and direction, that changes sign as a vortex passes over the wind sensor. This signal may be harder to detect when the wind is highly turbulent, or when a vortex does not pass directly over the sensor, but Kahanpää et al. (2016) noted that 87% of vortices detected via their pressure drops also had a strong signature in wind direction. Note that, while Coriolis forces constrain much larger vortex features (e.g. hurricanes) to rotate either clockwise or anti-clockwise depending on the hemisphere, for dust devil-sized vortices no preferred sense of rotation has generally been observed and only a tiny impact on sense of rotation is predicted by Large Eddy Simulation modeling (Ito et al. 2011). However, in situations where the mesoscale circulation is cyclonic or anticyclonic due to local weather patterns, dust devil-sized vortices are predicted – and have been observed – to share the same sense of rotation (Ito et al. 2011; Fujiwara et al. 2012).

### Aeolian Features and Activity

As on all past landed missions, the Mars 2020 rover does not carry any dedicated aeolian instruments, such as saltation detectors or sand/dust flux sensors. However, images from Mars 2020 cameras may be used to study the properties of sand grains and aeolian features, their motion, surface albedo changes, and dust devils or other dust-raising activity. High-frequency meteorological data may then be used to infer the cause of observed aeolian activity.

#### Orientation and Motion of Surface Aeolian Features

Like Mastcam, its predecessor on MSL, Mastcam-Z will be used to obtain high-resolution views of surface aeolian features (e.g. ripples, ventifacts, and dunes). In addition to determining the characteristics of these features, such images will also be used for “change detection” experiments, in which images taken of the same surface region at different times are co-registered to look for changes, which are then attributed to aeolian processes. These techniques have been used to determine the seasonal and diurnal variation in aeolian activity in Gale crater, especially within the Bagnold Dune Field (Bridges and Ehlmann 2017; Bridges et al. 2017; Baker et al. 2018a,b). Like MSL’s Curiosity rover, Mars 2020’s Perseverance rover also carries two Navcams and six Hazcams, which are enhanced by having a wider field of view and imaging in color. While these cameras have a lower resolution than Mastcam-Z, they are far less in demand when the rover isn’t driving, hence are good choices for making more frequent aeolian observations (Greeley et al. 2010; Baker et al. 2018b). Finally, Mars 2020 will also carry a suite of Descent Imagers, one of which will look down at the surface from underneath the rover, much like the Mars Descent Imager (MARDI) on MSL. While this camera is intended primarily for EDL, it may also be used after landing to image the surface beneath the rover when it is not in shadow, specifically around sunrise and sunset, extending imaging further into the early morning and late evening to help identify the timing of observed changes (Baker et al. 2018b). The size distribution of particles involved in aeolian activity is vital for interpreting such changes (Baker et al. 2018b; Weitz et al. 2018). On Mars 2020, detailed images of surface grains down to tens of microns will be available from the Scanning Habitable Environments with Raman and Luminescence for Organics and Chemicals (SHERLOC) micro-imagers at the end of the robotic arm (Bhartia et al. 2020, this journal).

Aeolian investigations on MSL have been hampered by a lack of good wind data throughout the mission (see Sect. 2.4). InSight has benefited from improved wind data, but has lower resolution cameras and lacks any significant aeolian features at its landing site (Golombek et al. 2020). Despite this, albedo and other changes seen by InSight have been used to explore possible thresholds for particle motion and to attempt to differentiate between large-scale wind-induced and vortex-induced lifting (Baker et al. 2020; Charalambous et al. 2021). This suggests that a combination of good wind (and pressure) data, higher-quality imaging, and more access to aeolian features for the Mars 2020 mission could provide enormous insight into aeolian processes and the relative importance of “wind stress” and “dust devil” dust lifting (Newman et al. 2002a,b). On longer timescales, a wind stress dataset spanning a full Mars year can be used to predict the long-term direction of motion of local dunes and the orientation of local aeolian features, including dunes, ventifacts, and yardangs. This can be used to understand both the characteristics of currently active features and to indicate that other features most likely formed under past climate conditions.

#### Dust Devils (Dusty Convective Vortices)

Multiple images of the same view have been used on all Mars surface missions since Mars Pathfinder to detect dust devils (Metzger et al. 1999; Greeley et al. 2010; Lemmon et al. 2017). Similar to MSL, the majority of dust devil monitoring on Mars 2020 will be performed using the Navcams, Hazcams and Mastcam-Z. This monitoring, which will consist of regular surveys (a few images covering all directions) and movies/videos (multiple images or a Mastcam-Z video covering up to 30 minutes in one direction) has two purposes: (i) to study the statistics of how dust devil number and size varies with time of sol, season, and location, and (ii) to determine the characteristics of dust devils, such as their dust content, direction of motion (indicating winds at that location), and height (which may be related to the height of the PBL; Fenton and Lorenz 2015). Whenever possible, simultaneous meteorological data are taken, so that if the dust devil vortex passes over (or near to) the rover then its impact on pressure, wind, temperature, and radiative fluxes can be correlated with the imaging and used to infer more about the dust devil’s characteristics. These measurements are important for relating vortex activity to other atmospheric variables (Newman et al. 2019a; Spiga et al. 2020); for understanding when and where surface dust is lifted, especially outside of dust storms when dust devil lifting may dominate (Basu et al. 2004; Kahre et al. 2006); and for understanding the likelihood of dust-devil-induced cleaning events on Mars 2020 and other missions, especially those that rely on cleaning of dust from solar panels to maintain power.

### Obtaining Wind Stress from Mars 2020 Meteorological Data

Wind stress, $\tau $, is the primary driver of aeolian activity, and is given by:

1 $$ \tau =\rho u_{*}^{2} $$

where $u_{*}$ is drag (or friction) velocity, and $\rho $ is air density, which is given by $P/(R'T)$, where $P$ is surface pressure, $R' = 191.272~\text{J}\,\text{kg}^{-1}\,\text{K}^{-1}$ is the specific gas constant for Mars, and $T$ is near-surface air temperature. Note that $u_{*}$ can be directly measured from very accurate, high frequency measurements of the 3-D wind field near the surface, via equation:

2 $$ u_{*} = \bigl[ \bigl( \overline{u'w'} \bigr)^{2} + \bigl( \overline{v'w'} \bigr)^{2} \bigr]^{1/4} $$

where $u '$, $v '$, and $w'$ are the turbulent fluctuations of the three wind components and the overbar represents Reynolds (i.e. time) averaging. The highest frequency range would be achievable with the accuracy and frequency of e.g. a sonic anemometer, which can provide faster sampling than MEDA’s wind sensors. However, MEDA’s 1 Hz is sufficient to reach into the inertial range given the large Kolmogorov scale on Mars (e.g. Tillman et al. 1994; Schofield et al. 1997; Murdoch et al. 2017).

The alternative is to estimate $u_{*}$ using a Businger-Dyer similarity relationship between the surface momentum flux and the mean vertical profile (Businger et al. 1971; Stull 1988). Specifically, $u_{*}$ is related to the measured wind, $u$, at some height, $z$, and the estimated surface roughness, $z_{0}$, by:

3 $$ u ( z ) = \frac{u_{*}}{\kappa } \biggl[ \ln \biggl( \frac{z-d}{z_{0}} \biggr) +\psi ( z, z_{0},L ) \biggr] $$

where $\kappa $ is the Von Kármán constant (taken to be 0.4), $d$ is the zero plane displacement (the height in meters above the ground at which zero wind speed is achieved as a result of flow obstacles), $L$ is the Obukhov length from Monin-Obukhov similarity theory, and $\psi $ is a stability term. In the absence of obstacles, d may be taken to be zero. Another simplifying assumption is to assume neutral stability, in which case $\psi $ is also 0. This assumption is likely to be incorrect for much of the Martian sol, e.g. during periods of convection (when the atmosphere is unstable) or at night if a strong inversion develops (hence the atmosphere is stable). However, under these assumptions, Eq. (3) becomes:

4 $$ u ( z ) = \frac{u_{*}}{\kappa } \biggl[ \ln \biggl( \frac{z}{z_{0}} \biggr) \biggr] \equiv \frac{u_{*}}{\kappa } \ln ( z ) - \frac{u_{*}}{\kappa } \ln ( z_{0} ) $$

which requires only $z_{0}$ to be estimated.

Estimating $z_{0}$ – especially for another planetary body – is not trivial, however. A viscous sublayer can exist near the surface in which surface friction causes viscous forces to dominate over inertial forces, resulting in smooth flow. The roughness Reynolds number, $\operatorname{Re}_{r} =\rho k_{s} u_{*} /\mu $, dictates the extent to which roughness elements on the surface disrupt this flow and its value determines how $z_{0}$ should be estimated. Here $\mu $ is the dynamic viscosity and $k_{s}$ is the Nikuradse roughness (Nikuradse 1933; White 2006), which is approximately equal to particle diameter, $D_{p}$, for a homogeneous bed of monodisperse spherical particles, but more generally given by two to five times the median particle size. For $\operatorname{Re}_{r} >60$, the flow is “aerodynamically rough” (i.e., $D_{p}$ is large enough that turbulent mixing destroys the viscous sublayer) and $z_{0} \approx k_{s} /30$. For $\operatorname{Re}_{r} <4$, the flow is “aerodynamically smooth” and $z_{0}$ is given by the thickness of the viscous sublayer, which depends on $u_{*}$ and is generally much larger. Due to its much lower atmospheric density than Earth, Mars is likely in the “smooth” regime over surfaces with small roughness elements (e.g. a smooth bed of sand). However, estimates of $z_{0}$ for Mars typically use the “rough” regime definition; see Kok et al. (2012) for discussion of why this is likely appropriate when saltation occurs. Surface roughness maps are therefore produced by assuming that $z_{0} \approx k_{s} /30$ and using orbital datasets related to the height and spacing of roughness elements to estimate $k_{s}$. Methods include using Mars Orbiter Laser Altimeter (MOLA) topography and roughness maps (Heavens et al. 2008) and Mars Global Surveyor (MGS) Thermal Emission Spectrometer (TES) rock abundance maps (Hébrard et al. 2012).

A further concern is that the functional forms of the stability terms, $\psi$, and the estimations used for $z_{0}$ are empirically derived for Earth and are not yet known to work the same way under Martian conditions. Even assuming neutral conditions, for which Eq. (4) may be used and stability terms neglected, the uncertainty in $z_{0}$ is a concern. A more accurate estimate of $u_{*}$ – referred to as the flux profile method (e.g. Bi et al. 2015) – is useful as it also provides an estimate of $z_{0}$ under the neutral stability assumption. The method involves measuring $u ( z )$ at several heights, from which $u_{*}$/$\kappa $ is obtained as the slope of the least squares fitting of $u$ and $\ln(z)$; see RHS of Eq. (4). The intercept on the y-axis is then $\frac{u_{*}}{\kappa } \ln ( z_{0} )$. Unfortunately, given that MEDA measures wind at a fixed height, this method cannot be applied on Mars 2020, but it was applied to Mars Pathfinder wind sock data, which were available at three heights (Sullivan et al. 2000).

Finally, it is important to note that, unlike Earth, the air density on Mars may change by several tens of percent from day to night, and by an even greater amount with season, due to the strong diurnal and seasonal variations in surface pressure and temperature. Hence the force exerted on surface particles, as given by the wind stress, is not simply related to wind speed or even $u_{*}$. This means that wind speed is not a completely reliable indicator of when saltation is expected to occur. For example, it is possible for the peak wind stress to occur overnight (when densities are highest due to low air temperatures) while peak wind speeds occur during the daytime (e.g. Baker et al. 2018a, 2021). Thus it is critical to consider the estimated wind stress, rather than simply the wind speed at $\sim1.6~\text{m}$, when interpreting aeolian features and activity.

## Atmospheric Models and Setups Used in This Study

This study uses output from seven different Mars atmospheric models, two of which (Ames and MarsWRF, Sects. 3.4 and 3.5) are run in both “low-resolution” and “high-resolution” mode. This gives a total of nine simulations, with four run at high spatial resolution over Jezero crater (between $\sim1.4$ and 10 km horizontal grid spacing) and the others run at relatively low spatial resolution (between $\sim120$ and 300 km); see Table 1. Due to the high computational expense of running at high resolution, the full seasonal cycle is only predicted using the low-resolution simulations, while it is sampled at the landing time (∼northern spring equinox) and at up to eleven other times of year by the high-resolution simulations. Sections 3.1–3.7 describe the seven models and provides more details of how they were set up for this intercomparison study, while Sect. 3.8 describes how the model output was processed to provide a direct comparison with the fields that Mars 2020 will observe.

Unless otherwise specified, all models used surface topography derived from MOLA data (Smith and Zuber 1996; Smith et al. 2001), while surface albedo and thermal inertia over most of the surface are derived from MGS TES observations (Christensen et al. 2001; Putzig et al. 2005; Putzig and Mellon 2007), although polar ice properties are typically used as tuning parameters for the CO_2_ cycle (see Sect. 6.1). Despite this, the surface albedo and thermal inertia interpolated to the landing site differ between models. This is due to how the observed values are interpolated and smoothed onto the model grid and due to the size of the model grid cell in each simulation. Aerodynamic surface roughness, $z_{0}$, is set to be spatially uniform or according to a map derived from MOLA intrashot data (Garvin et al. 1999) or MGS TES rock abundance data (Hébrard et al. 2012), as shown in Table 1. Table 1 also provides the grid spacing and surface properties at the landing site in all nine simulations and is a useful reference for interpreting the differences between model results described in Sects. 5 and 6. While surface properties over a wider area will also influence the circulation observed at the landing site, local values of albedo and thermal inertia will have the largest impact on surface and atmospheric temperature, and – as on past missions – such observations will be used to infer the variation of these surface properties along the rover traverse (Hamilton et al. 2014; Vasavada et al. 2017).

Atmospheric dust content is a major control on the atmospheric thermal state and thus circulation of Mars (e.g. Gierasch and Goody 1972; Wilson and Hamilton 1996; Kahre et al. 2017; Wolff et al. 2017). Dust content can vary significantly from year to year, making it by far the main source of interannual variability in Martian climate. The greatest year-to-year variations occur primarily during the so-called “dust storm season,” $\text{Ls}\sim 180$ to $360^{\circ}$. Dust loading affects the strength of the large-scale circulation and thermal state, and changes in the horizontal and/or vertical variation of dust affect thermal tides and other planetary-scale waves, modifying diurnal cycles of pressure and winds at the surface. For these reasons, all simulations presented here were conducted using a seasonal variation of dust loading that corresponded to no major dust storms occurring in that year. However, the specific dust distributions were left to each modeling group to decide and ultimately reflect a broad set of choices for what best represents a “storm-free” year. The dust distributions and their evolution with time are described in each of the following sections. In addition, Table 2 provides the visible column dust opacity over the landing site at four times of year, but note that the vertical dust distribution and particle size distribution may also be crucial.

**Table 2:** Daily mean visible column dust opacity over the landing site at the four key seasons examined in Sects. 5 and 6, and a summary of the dust scenario used, for each of the nine simulations

Simulation	Visible dust opacity for season shown				Dust scenario used (see Sects. 3.1.1 to 3.7.1 for more details)
	Ls ∼ 5^∘^	Ls ∼ 90^∘^	Ls ∼ 180^∘^	Ls ∼ 270^∘^	
GEM-Mars	0.26	0.18	0.19	0.52	Interactive dust with dust devil and saltation lifting
LMD global	0.31	0.26	0.35	0.62	Interactive dust but rescaled to match dust maps from years without global storms
OpenMARS	0.32	0.26	0.30	0.61	Assimilated temperature and dust from MY32
Ames low-resolution	0.30	0.26	0.4	0.63	Interactive dust but with lifting constrained to match MY30 dust opacity map
MarsWRF global	0.30	0.19	0.33	0.44	Prescribed using TES nadir and limb dust opacities for years without global storms
LMD mesoscale	0.31	N/A	N/A	N/A	*As in LMD global model*
Ames high-resolution	0.30	0.26	0.4	0.63	*As in Ames low-resolution model*
MarsWRF mesoscale	0.30	0.19	0.33	0.44	*As in MarsWRF global model*
MRAMS	0.33	0.22	0.23	0.54	Prescribed using MY24 TES nadir dust maps and SPICAM vertical profiles

While specifying the dust identically in all model simulations would have permitted the most direct assessment of the impact of model dynamics, physics, and resolution on results, this was not possible given the scope and timeline of this study. However, the simulations shown here are those that each modeling group commonly use as their “storm-free” predictions, hence any additional spread in results is representative of the spread in model predictions that have been and may in future be used for a variety of scientific and engineering purposes. In addition, this enables us to compare the impact of different approaches used to specify dust.

### The GEM-Mars Atmospheric Model

The GEM-Mars model (Neary and Daerden 2018; Daerden et al. 2019; Neary et al. 2020) is a gridpoint-based general circulation model of the Mars atmosphere based on the GEM (Global Environmental Multiscale) model, part of the operational weather forecasting and data assimilation system for Canada. The model extends from the surface to approximately 150 km and simulates interactive carbon dioxide, dust, water, and atmospheric chemistry cycles. Dust and water ice clouds are radiatively active. The dynamical core uses a semi-Lagrangian advection scheme with a two-time-level semi-implicit integration method that allows for a relatively long time step while maintaining stability. The simulations for this study were performed at a horizontal resolution of $4^{\circ}\times 4^{\circ}$ with 103 unevenly-spaced log-hydrostatic pressure levels. The height of the lowest atmospheric layer is set to 2 m, with the next level up at $\sim13~\text{m}$. A time step of 1/48th of a Mars solar day (sol) was used.

#### Dust Distribution in the GEM-Mars Simulation

This simulation is the only one in the study to have a 3-D dust distribution that is fully self-consistent with the model circulation, rather than being directly constrained by observations. Size-distributed dust is injected according to parameterized saltation and dust devil dust lifting, followed by dust transport by advection, mixing, and sedimentation, as in (Musiolik et al. 2018). The saltation dust lifting parameterization uses a lower threshold wind stress than is typical, based on results of low-gravity experiments, and the overall dust lifting parameters are then tuned such that running the simulation provides a generally realistic variation of dust loading over the year. There is a gradual increase in dust loading after about southern spring equinox, especially in the southern hemisphere, peaking at a visible (0.67 micron) opacity of $\sim0.8$ at $\text{Ls}\sim 270^{\circ}$ at $\sim 40^{\circ}$ S latitude (Musiolik et al. 2018).

### The LMD Global Atmospheric Model

The Laboratoire de Météorologie Dynamique (LMD) Mars global circulation model (GCM) has been developed over the past 25 years (Forget et al. 1999; Lewis et al. 1999) in collaboration with LMD, Oxford University, the Open University (OU), and the Instituto de Astrofisica de Andalucía. Fluid dynamics equations for the atmosphere are solved in a finite-difference grid point dynamical core. Physical parameterizations of processes unresolved by the hydrodynamical solver include a representation of the most salient characteristics of the carbon dioxide, dust, and water cycles, with dust and water being transported by the model circulation and with both dust and water-ice particles being radiatively active (Madeleine et al. 2011; Navarro et al. 2014). A two-moment scheme is used for dust particles; this means that the dust mixing ratio and number concentration are both carried, such that the dust particle size distribution at any time and location may be inferred. The GCM includes the radiative effects of water ice clouds (Madeleine et al. 2012) that are produced by a complete water cycle model using a microphysical scheme that calculates the growth of water ice crystals onto dust nucleation cores (Navarro et al. 2014). The PBL daytime convective processes are represented by a specific “thermal plume” model described in Colaïtis et al. (2013). The baseline simulation has 64 by 48 grid points, corresponding to a resolution of $5.625^{\circ}$ in longitude by $3.75^{\circ}$ in latitude. The vertical layers are distributed using a hybrid sigma-pressure coordinate system with the first model layer centered at $\sim 4.5$ m; the model top is located at $\sim 250$ km.

#### Dust Distribution in the LMD GCM Simulation

In this simulation, the horizontal distribution is constrained to match observations from years without major dust storms, with the vertical distribution determined more self-consistently by the model. In the LMD model’s semi-interactive dust scheme, two-moment dust lifting is performed according to dust devil and wind stress parameterizations, but the atmospheric dust column is then rescaled to match daily maps obtained from observations by orbiting spacecraft (Madeleine et al. 2011). For this simulation, column dust opacities averaged over MYs 24, 26, 27, 29, 30 and 31 (well-observed years with no major storms) were used to produce average “storm-free year” dust maps as a function of season (Montabone et al. 2015).

### The OpenMARS Database

Data were taken from GCM assimilations of previous Mars years, as archived in the OU’s OpenMARS database (Holmes et al. 2019, 2020). Data assimilation is conducted for column dust opacities and thermal profiles (Lewis et al. 2007; Montabone et al. 2006), with water vapor and ice (Steele et al. 2014a,b), ozone (Holmes et al. 2018), and carbon monoxide (Holmes et al. 2019) also assimilated when available. The model used for assimilation is the OU version of the LMD GCM, which shares the LMD GCM’s surface property maps and physical sub-models (see Sect. 3.2) but uses a semi-spectral dynamical core and a semi-Lagrangian conservative tracer transport scheme (Newman et al. 2002a,b). It also includes a gravity wave drag scheme that includes low-level drag from sub-gridscale orography derived from the MOLA $1/32^{\circ}$ data set (Collins et al. 1997). Although the model has a full water cycle scheme, and TES water data were assimilated, the simulation did not include radiatively-active ice clouds, in order to ensure stability. The assimilations used were conducted using a spectral truncation of dynamical model fields at wavenumber 31 (T31, with a $3.75^{\circ}$ horizontal grid for dynamical products) and $5^{\circ}$ (300 km) horizontal grid for the physical sub-models, with 35 vertical sigma levels stretched up to about 100 km altitude and the lowest level at $\sim5~\text{m}$ above the surface.

#### Dust Distribution in the OpenMARS Simulation

As for the LMD GCM simulation, here the horizontal distribution is constrained to match observations from years without major dust storms, with the vertical distribution determined more self-consistently by the model. Temperature profiles and total column dust opacities measured by MCS in MY32, a year with no major dust storm, are assimilated. The vertical dust distribution is determined via the semi-interactive scheme described in Sect. 3.2.1 (Madeleine et al. 2011), with the atmospheric dust column at each grid point (i.e. the horizontal dust distribution) now rescaled to match MY32 observations via the assimilation process.

### The NASA Ames Mars Atmospheric Model

The Ames Mars Global Climate Model (MGCM) is based on the National Oceanic and Atmospheric Administration (NOAA)/Geophysical Fluid Dynamics Laboratory (GFDL) cubed-sphere finite volume (FV3) dynamical core and includes physical process routines developed at NASA Ames and NOAA/GFDL specifically for Mars conditions (Haberle et al. 2019; Bertrand et al. 2020). Planetary boundary layer physics are included based on the level-2 Mellor and Yamada (1982) parameterization, with turbulent fluxes computed using Monin-Obukhov similarity theory (Haberle et al. 1993, 1999). A two-stream correlated-k radiative transfer scheme is used to account for the effects of CO_2_ and H_2_O gas and atmospheric aerosols (Toon et al. 1988; Lacis and Oinas 1989). The physics of water sublimation, transport, and cloud microphysical processes (nucleation, growth, and sedimentation) are available in the model but cloud formation was deactivated for the present study.

Results from two Ames MGCM simulations are used here: one with a horizontal resolution of $1/8^{\circ}\times1/8^{\circ}$ and 30 vertical layers, and one with a horizontal resolution of $2^{\circ}\times2^{\circ}$ and 37 vertical layers. A hybrid sigma-pressure grid is used in the vertical, with decreasing resolution as altitude increases. In both cases, the midpoint of the bottom layer is at 5 m. The high resolution simulation was run for 8 sols at each of the $L_{s}=5$, 90, 180, and $270^{\circ}$ seasons, while the lower resolution simulation was run over a full annual cycle.

#### Dust in the NASA Ames Simulations

Similar to the LMD and OpenMARS simulations, the horizontal distribution is constrained to match observations from years without major dust storms, with the vertical distribution determined more self-consistently by the model. The difference is that, rather than injecting dust according to parameterizations and then rescaling the dust column, the amount of dust lifted is instead chosen such that the evolving horizontal dust distribution matches observed column opacity maps (Kahre et al. 2009). In this case, the simulations used maps for MY30, a year with no major dust storms (Montabone et al. 2015). A two-moment lognormal distribution of dust with a specified effective radiative particle size (2 μm here) is injected from into the lowest atmospheric model layer at each physics scheme timestep when the simulated dust column opacity is lower than that in the dust map for that location and time of year. The injected dust is then mixed by sub-gridscale processes, transported by model resolved winds, undergoes gravitational sedimentation, and is radiatively active (Haberle et al. 2019; Bertrand et al. 2020).

### The Mars Weather, Research and Forecasting (MarsWRF) Multiscale Atmospheric Model

The planetary Weather Research and Forecasting (WRF) model (Richardson et al. 2007), planetWRF, is modified from the widely-used National Center for Atmospheric Research (NCAR) WRF model (Skamarock and Klemp 2008; Powers et al. 2017). It is unique among planetary atmospheric dynamical models in being able to simulate the whole globe (acting as a GCM), to simulate nested higher resolution domains within a global context (acting as a two-way nested mesoscale model), and to simulate microscale turbulent motions (acting as a LES). The Mars instantiation, MarsWRF, includes the treatment of radiative transfer in the Martian atmosphere, including the effects of carbon dioxide gas and ices, aerosol dust, and water vapor and water ice (Mischna et al. 2011; Lee et al. 2018). A full description of the model dynamics, setup, and surface properties is provided in Richardson et al. (2007) and Toigo et al. (2012). The model also includes fully interactive cycles of carbon dioxide, dust, and water; however, the simulations used here do not include the effects of water vapor or ice, and the time-evolving, 3-D atmospheric dust distribution is prescribed (see below). The model’s radiative transfer, PBL, surface, and subsurface schemes are all identical to those described in Newman et al. (2017), as are the surface property maps described there. Vertical grid A shown in Table 2 of Newman et al. (2017) is used in this work. It consists of 43 layers from the surface to $\sim80~\text{km}$, with greater vertical resolution in the lowest $\sim12~\text{km}$ of the atmosphere. This grid has three layers with their midpoint below 105 m and the lowest layer midpoint at $\sim10~\text{m}$ above the surface.

**Fig. 1:**
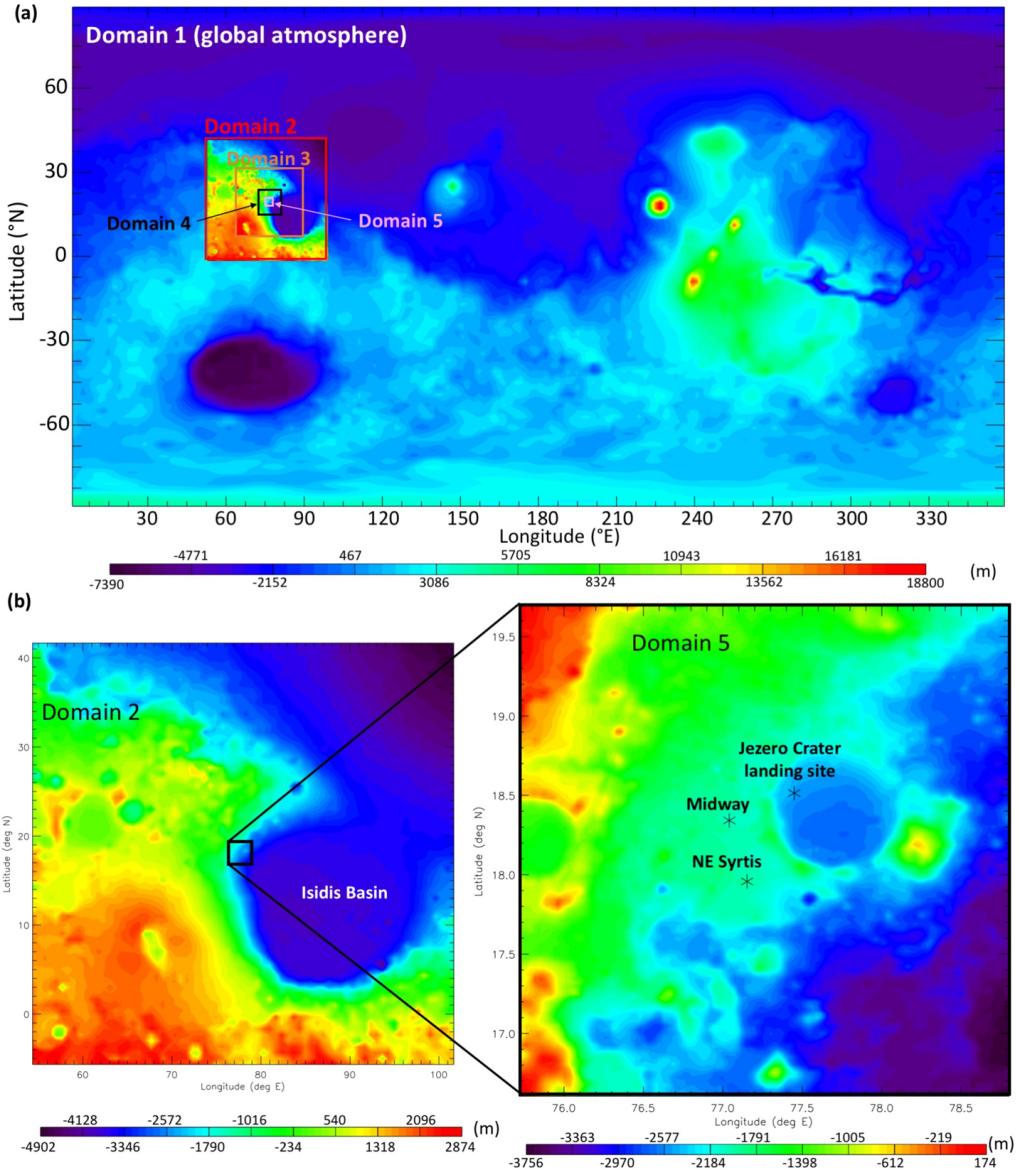
(**a**) MOLA topography in MarsWRF global domain (domain 1) and nests (domains 2–5). Note contours are rescaled for each domain to cover only the range of heights involved. (**b**) MOLA topography for domains 2 and 5, with the Mars 2020 landing site and two potential additional rover stops labeled

Results from two types of MarsWRF simulation are used here: the first type has only a global domain (d01) and is run for a full annual cycle, while the second type has five domains in total including four “nests” (d02–d05), each of which sits within its parent domain and covers a smaller area at three times the horizontal resolution. These “nested” simulations are far more computationally expensive than the global-only simulation, so are only run for 8 sols every $30^{\circ}$ of Ls over a full Mars year, with the first sol of each simulation discarded as spin-up. For the nested simulations, MarsWRF uses a nearly identical configuration to that used to simulate Gale crater in Newman et al. (2017, 2019a), except for the nests now being centered on Jezero crater; see Fig. 1. The horizontal grid spacing in d01 is $2^{\circ}$ globally, with the resolution increasing by a factor of 3 in each subsequent domain, giving a horizontal grid spacing of $\sim1.5~\text{km}$ in d05.

#### Dust Distribution in the MarsWRF Simulations

Unlike the preceding models, in these simulations the 3-D dust distribution is fully prescribed. Both the global and nested simulations use a dust distribution based on TES dust observations in years without global dust storms (Guzewich et al. 2013). Dust vertical profiles are based on TES limb measurements, interpolated spatially and temporally (including a sinusoidal interpolation between 2 pm and 2 am values). Because TES may not observe the lowest regions of the atmosphere, the integrated opacity from the vertical dust profile may miss dust in the lowest atmospheric regions. The total column opacity is therefore scaled to match concurrent TES nadir column opacities. It has been suggested that injecting the additional dust into the lowest atmospheric regions would be more consistent (Natarajan et al. 2015) but that option is not used here; instead, the mass mixing ratio in each layer is adjusted by the same percentage, chosen such that the sum of layer opacities matches the TES nadir column.

### The LMD Mesoscale Atmospheric Model

The LMD Mars Mesoscale Model (MMM) couples the hydrodynamical solver from the terrestrial limited-area mesoscale WRF model (also the basis for the multiscale planetWRF model described above). The LMD-MMM integrations use, as initial and boundary conditions, outputs from the LMD GCM obtained with the exact same physical parameterizations (code and settings), except for sub-gridscale parameterizations for topographic gravity-waves being included in GCM integrations. Details of the LMD-MMM are available in Spiga and Forget (2009). The generic configuration of the LMD-MMM for the Jezero simulations reported herein is close to that described in Spiga et al. (2017). A horizontal domain is defined for studying the environmental conditions at the Mars 2020 landing and operation sites: $181\times181$ grid points with mesh spacing of 10 km, centered at reference latitude $18.466~^{\circ}\text{N}$ and longitude $77.430~^{\circ}\text{E}$, Mercator map projection. Surface static properties in this domain are as for the global model but now interpolated from the highest-available-resolution fields. The vertical grid is composed of 61 levels with a model top at 5 Pa. Vertical levels are approximately equally spaced in altitude (resolution 800 m) with a grid refinement close to the surface giving a lowest model level $\sim4\text{--}5~\text{m}$ above the surface. The model was run for three sols at $\text{Ls}\sim 5^{\circ}$ only, with the first sol discarded as spin-up.

#### Dust Distribution in the LMD Mesoscale Simulation

This is as in the LMD global simulation; see Sect. 3.2.1.

### The Mars Regional Atmospheric Modeling System (MRAMS) Model

A full description of the MRAMS model configuration is included in Pla-Garcia et al. (2016). For this study, MRAMS is configured with five grids centered over the Jezero crater landing site, as shown in Online Resource 1. The horizontal grid spacing at the center of the five grids is 240, 80, 26.7, 8.9 and 2.96 km, respectively. All the grids have the same vertical grid configuration with the vertical winds staggered between thermodynamic levels. The lowest thermodynamic level (where temperature and pressure are prognosed) is $\sim14~\text{m}$ above the ground. This vertical spacing is gradually stretched with height until reaching a maximum spacing of 2500 m, and the levels gradually transition from terrain-following near the surface to constant altitude by the top of the model. The spacing does not exceed 100 m in the lowest 1 km, and does not exceed 400 m in the lowest 4 km. The model top is at 51 km with 50 vertical grid levels. Initial and boundary conditions for the simulations, including the CO_2_ ice distribution (which is then held fixed), come from the Legacy version of the NASA Ames GCM (Haberle et al. 2019). For $\text{Ls}\sim 0^{\circ}$, the model was run for twelve sols with the first two “spin-up” sols discarded; for $\text{Ls}\sim 90$, 180, and $270^{\circ}$, the model was run for four sols with the first two sols again discarded. The output frequency here is five Mars minutes and output from domain 5 (grid spacing $\sim2.96~\text{km}$) is used, which is comparable to MarsWRF’s domain 5 grid spacing.

#### Dust Distribution in the MRAMS Simulations

Similar to MarsWRF, the 3-D dust distribution is fully prescribed but unlike that in MarsWRF it is longitudinally-uniform (except for the effects of topography). The horizontal dust distribution is based on zonally-averaged dust column opacities observed by TES in MY24, a year with no global storm. However, the vertical dust distribution is based on Mars Express Spectroscopy for Investigation of Characteristics of the Atmosphere of Mars (SPICAM) UV solar occultation data (Määttänen et al. 2013).

### Processing of Model Output

The MEDA sensors will sit at a height of $\sim1.5~\text{m}$ above the surface. This is well below the lowest layer at which atmospheric variables are prognostically determined in any of the models, so it is necessary for us to extrapolate the model-predicted winds and atmospheric temperatures in the lowest model layer down to 1.5 m. This extrapolation may be performed inside the model during the simulation, in which case the stability function (included in Eq. (3)) at the time of the prediction is known and can be used. This method is used for MarsWRF, hence all MarsWRF output is provided at 1.5 m.

Alternatively, the extrapolation may be done post-simulation, as follows:

#### Extrapolation of Winds to 1.5 m

For winds, this is done using the model-output winds at some height ($z_{2}$) and assuming a neutral stability condition (Eq. (4)):

5 $$ u ( z_{2} ) = u ( z_{1} ) \frac{\ln ( \frac{z_{2}}{z_{0}} )}{\ln ( \frac{z_{1}}{z_{0}} )} $$

where $z_{2}$ is the height of the lowest model layer, $u(z_{2})$ is the modeled wind there, $z_{1}$ is 1.5 m, and $z_{0}$ is the roughness height used at the landing site in each model, which depends on the roughness map assumed. Table 1 includes a list of surface properties at the landing site in each model, including $z_{0}$. In all cases shown, the predicted wind speed has been extrapolated to 1.5 m using the roughness heights used in each simulation.

#### Extrapolation of Atmospheric Temperatures to 1.5 m

A range of methods exist to extrapolate atmospheric temperature but this has not been well-established for Mars conditions. In most cases, predicted atmospheric temperature is therefore shown at the height of the lowest model layer (which ranges from 2 m in GEM-Mars to $\sim5~\text{m}$ in most simulations to 14.5 m in MRAMS). The exceptions are MarsWRF, which extrapolates to 1.5 m inside the model, and the OpenMARS results, for which the method of Petrosyan et al. (2011) was used to interpolate in potential temperature between predicted surface temperature and potential temperature at $\sim5~\text{m}$, following a logarithmic profile. This method was validated by comparing with the results of a higher vertical resolution simulation.

Note that the extrapolated atmospheric temperature is only sensitive to the interpolation method during the middle of the day; at night, the surface and atmospheric temperatures are very similar. To give a sense of the maximum impact on results, MarsWRF output was interpolated to 14.5 m (the MRAMS output level) using the Petrosyan et al. (2011) method. The result was a drop of $\sim5~\text{K}$ in atmospheric temperature compared to the 1.5 m MarsWRF predictions at 15:00 (roughly when atmospheric temperatures peak).

## Predicting Aeolian Activity from Atmospheric Model Output

This section provides the theory needed to convert the meteorological variables output from the above simulations into predicted sand fluxes, bedform motion and orientation, and dust devil activity as defined in Renno et al. (1998).

### Calculating Sand Fluxes

Over recent decades, a number of equations have been constructed to calculate the sand flux for a given wind stress. These are based on field studies (e.g. Bagnold 1941), wind tunnel experiments for a subset of Mars conditions (e.g. Greeley et al. 1980; Merrison et al. 2008), and theoretical calculations (e.g. Kok 2010). For a summary of the many options available, see e.g. Kok et al. (2012). In this study, we assume a relatively straightforward formula that relates wind stress to sand flux:

6 $$ Q \propto \rho u_{*}^{2} \bigl(u_{*} - u_{*}^{t} \bigr) $$

where $Q$ is sand flux and $u_{*}^{t}$ is the threshold drag velocity for saltation (related to the threshold wind stress via Eq. (1)). This was first proposed by Lettau and Lettau (1978), although is sometimes erroneously referred to as the Fryberger (1979) equation. The predicted sand flux is then calculated by choosing the wind stress threshold above which saltation will occur.

A longstanding issue for Mars is the widespread evidence of aeolian activity (dunes, ripples, etc.) despite wind stresses being potentially well below the threshold required for saltation (e.g. Sullivan and Kok 2017). In fact, there are two relevant thresholds: a “fluid” threshold, which is the wind stress required to initiate full bed saltation, and an “impact” threshold, which is the wind stress required to continue this saltation once it has begun. The latter threshold is lower, because saltating grains returning to the surface, having extracted momentum from the boundary layer during the higher portions of their trajectories, add to the force on the surface; thus less fluid drag is needed to maintain saltation than to initiate it. On Earth, the impact threshold is $\sim80\%$ of the fluid threshold, whereas numerical modeling for Mars conditions estimates it ranging between $\sim5$ and 40% of the fluid threshold there (see e.g. Kok 2010; Sullivan and Kok 2017). Sullivan and Kok (2017) postulate that sporadic mobilization of sand grains occurs below the traditional fluid thresholds required for full bed saltation. The low Martian gravity allows these grains to gain energy as they bounce along the surface, giving rise to more grain motion, and producing ripples and other features but with very low sand fluxes. By these means, wind stresses below traditional fluid thresholds can give rise to saltation eventually, and this concept is supported by recent wind tunnel measurements (Swann et al. 2020). Another study, now based on low-gravity saltation experiments, suggests that the estimated fluid threshold is much lower than previously believed on Mars, due to the low gravity reducing the number of contacts between particles and thus decreasing the interparticle cohesion (Musiolik et al. 2018).

A final – but significant – issue is that low-resolution simulations (with grid spacings of $\sim100~\text{km}$) cannot capture wind gusts associated with e.g. local topography or wave perturbations (e.g. Toigo et al. 2012), and even mesoscale models (with grid spacings of $\sim10~\text{km}$) cannot resolve the short-lived gusts that are associated with daytime convective cells and vortices (e.g. Toigo et al. 2003; Klose et al. 2016). Thus even if we knew the actual threshold wind stress appropriate to Jezero crater’s soil composition etc., the winds predicted by the simulations used in this study may not exceed it at all, or may not exceed it over a realistic fraction of a Mars year.

In this study, we therefore make aeolian predictions for a range of plausible wind stress thresholds, from 0 Pa (which is clearly unphysical but provides an upper limit on sand motion) to 0.01 Pa (which was found to be a good effective threshold for matching observed seasonal dune migration using wind stresses from a low-resolution simulation; see Ayoub et al. 2014). Predicted sand fluxes and net sand transport directions over this range of thresholds, as a function of season, are predicted at the Mars 2020 landing site in Sect. 8.1 and at several points along the expected rover traverse in Sect. 8.3.

### Predicting Bedform Migration Speeds and Directions Based on the Net Annual Sand Flux Vector

A reasonable assumption in aeolian theory is that bedforms that migrate or elongate do so in the direction of the net sand flux vector when observed over some moderately long time period (Wilson 1971). For small and large ripples, this may be hours and weeks, respectively; for large, active dunes, this may be seasons to years. The dune migration direction may therefore be estimated by calculating the net sand flux vector multiple times per Mars sol over a full Mars year (or by sampling the diurnal cycle at regular intervals over a Mars year), as described in Sect. 4.1, then performing a vector sum over the year. These results are shown for the landing site in Sect. 8.2.

### Predicting Bedform Orientations Based on Annual Sand Fluxes

Below, we describe two theories by which bedform orientations may be predicted from wind data or model output. The correct theory to apply in a given location depends on the sand availability and spread in wind directions. In Sect. 8.4 we provide predictions for MarsWRF mesoscale model output over the Jezero region using both theories.

#### Bedform Orientations Using the Gross Bedform-Normal Transport (GBNT) Theory

The development of the Gross Bedform-Normal Transport (GBNT) theory of Rubin and Hunter (1987) provided the first means of predicting dune orientations given a known wind field. This approach has been verified in laboratory experiments (Rubin and Hunter 1987; Rubin and Ikeda 1990; Reffet et al. 2008), numerical modeling (Werner and Kocurek 1997; Kocurek and Ewing 2005; Reffet et al. 2008), and field studies (Lancaster et al. 2002; Rubin et al. 2008), and is widely accepted as producing the most accurate prediction of dune orientations in regions with loose, free-moving sediment for all dune types. The basic idea is that dunes will align so as to maximize the GBNT, which is simply the magnitude of wind-driven sand transport normal to the bedform, summed over all orientations between 0 and $180^{\circ}$ and over all times. The key here is to consider gross rather than net sand transport; transport to and fro across a dune crest cancels out in a net sense, but in reality both directions of transport move sand across the bedform and can thus build it over time. The optimal dune orientation is found by determining the orientation that results in the largest GBNT given the long-term (e.g. annual) wind field at a given location.

In Sect. 8.4, we use seven sols of mesoscale MarsWRF output every $30^{\circ}$ of Ls over a Mars year to calculate the predicted sand flux (Eq. (6)) and direction for each grid point over the Jezero region, for a range of threshold wind stress values. For each Ls period we calculate the sand flux and direction every ten minutes through each sol, then average over all seven sols for each of these 144 times of sol. We then interpolate from results at these twelve times of year to obtain estimated sand fluxes every ten minutes across a full Mars year. At each time and grid point, we calculate the sand transport perpendicular to each of 180 possible dune orientations (from 0 to $179^{\circ}$ in intervals of $1^{\circ}$), then sum over the entire year for each orientation and location. The orientation with the largest summed GBNT is the predicted “GBNT” dune orientation for that location.

#### Bedform Orientations Using the Fingering Mode Theory

Despite success in many regions, some bedforms were known to not follow the GBNT theory, and instead formed linear dunes roughly parallel to the direction of transport. This was finally explained by Courrech du Pont et al. (2014) and Gao et al. (2015), who developed the Fingering Mode theory based upon theoretical modeling and laboratory experiments. Basically, when the supply of sediment is limited, the production of a bedform is then not so much due to the arguments contained in the GBNT theory – which work if one considers an area filled with sand that is being moved around – but is instead produced by sand migrating away from its original position. In the supply-limited case, the bedform is effectively produced by migration and elongation of a sand pile, hence its orientation is typically similar to that found in Sect. 4.2 for the migration direction: the direction of net annual sand transport.

As for the GBNT approach, for each location we use MarsWRF mesoscale output to predict the sand flux vector every 10 minutes over a full Mars year for a range of thresholds, then use the equations described in the Supplementary Material of Courrech du Pont et al. (2014) to calculate the predicted “Fingering Mode” dune orientation for that location. The predicted orientations are nearly always within 5% of the net sand transport vector (Sect. 4.2).

### The Value of Predicting Bedform Orientations and Migration Directions

On smaller scales than these simulations’ grid spacing – i.e., $\sim\text{km}$ or smaller – bedform migration directions and orientations may be controlled by winds associated with topography that the simulations do not resolve. The same is true of abrasion features such as ventifacts or yardangs, the orientations of which are generally tied to the dominant wind stress direction, but which are not predicted here. On even smaller scales, bedforms and other aeolian features affect the flow themselves; for example, ripples on a dune’s surface were formed in winds modified by the dune itself. The present work is thus better able to predict larger-scale features that may be seen from orbit as well as from the surface, or smaller-scale features sitting on flat, featureless areas, than it is able to predict the finer features that may be encountered if the rover moves into a region of significant small-scale topography. However, this work should provide a general picture for what we expect in the Jezero region in general.

In addition, comparing predictions of bedform migration directions and orientations with what is seen from orbit provides a way of testing our modeled circulation for the whole area before we have even landed – and even after landing, before we have explored the vast majority of the region. As demonstrated in e.g. Newman et al. (2017), it is possible to determine the more realistic of two wind models by assessing how well they predict large-scale aeolian features, in that case the migration direction of the Bagnold Dunes in Gale crater. Once Mars 2020’s mission has begun and we begin taking wind data, we will compare the wind dataset to output from the set of simulations presented here, the goal being to identify where the models are going wrong (e.g. making the wrong assumptions, using the wrong physical parameterizations, etc.) and to improve them until they better match MEDA wind measurements. We can further test the improved models by making new predictions of aeolian characteristics across the region, and checking that they are also a better match to orbital observations.

### Predicting Dust Devil Activity

Because vortices are far smaller than a mesoscale atmospheric model’s grid spacing, they cannot be predicted directly by mesoscale or global scale models. Instead, in Sect. 9 the theory of Renno et al. (1998) is used to calculate a “dust devil activity” (DDA) based on the large-scale atmospheric state predicted by the MarsWRF mesoscale model. Note that despite the name, this theory applies equally to clear and dust-filled vortices. Complete details of how the theory is applied to MarsWRF output is provided in Sect. 3.2 of Newman et al. (2019a), thus we provide only a brief summary here. In the theory, convective vortices are modeled as convective heat engines, resulting in the DDA being set proportional to the sensible heat flux, $F_{s}$, multiplied by the vertical thermodynamic efficiency of the heat engine, $\eta $. The former depends primarily on the drag velocity and surface-to-air temperature difference, while the latter increases with the PBL depth.

The theory has been shown to have value when capturing effects related to dust devils and vortices on Mars. It is regularly used to parameterize the dust lifting by dust devils (and implicitly, other small-scale, turbulent phenomena) in Mars atmospheric models, by setting the amount lifted proportional to the DDA (Newman et al. 2002a,b). Doing so appears to successfully capture the global “background” dust as a function of season, outside of dust storms (Basu et al. 2004; Kahre et al. 2006). The theory has also been tested by comparing the predicted spatiotemporal variation of DDA with the number of vortex pressure drops detected by MSL over the first year (Kahanpää et al. 2016) and first three years of its mission (Newman et al. 2019a) and with the first half of the Mars year measured by InSight (Baker et al. 2020). The DDA is a measure of the energy that may be harnessed by vortices, but how this should be distributed into a vortex distribution is not clear at present. For example, should an increase in DDA result in a larger number of vortices, or stronger vortices (with greater pressure drops), or both? This is currently unclear. However, predictions of the seasonal and diurnal variation of DDA should provide a sense of when we expect to observe more and/or larger vortices, in the form of vortex pressure drops and also dust devils, if sufficient dust is available to be lifted. Such predictions also tell us when we should expect more dust to be removed (by dust devils) from rover surfaces or from samples cached on the surface.

The DDA theory has been called into question by Spiga et al. (2020), who argue that IR radiative heating of the near-surface atmosphere is more important than sensible heating for Mars (unlike Earth) and should be considered. As part of a separate study, we have recently investigated the sensible vs. IR radiative heating of the near-surface atmosphere in MarsWRF and find that the two are generally of comparable magnitude (Wu et al. 2020). Further, they have a generally similar variation with time of sol due to their strong dependence on temperature. However, work is needed to incorporate both heating effects into a revised DDA theory.

Spiga et al. (2020) also note a strong “observer effect” when determining the number of vortices using meteorological measurements, which leads to a strong correlation between wind speed and measured vortex activity. This arises because more vortex detections will occur when there is a background wind (which advects them over meteorological sensors) than if the background atmosphere is at rest. Thus, while the DDA may be used to predict the number of vortices expected in a given area at one instant – e.g. for comparison with orbital imaging of the number of dust devils in different regions, or with imaging from a rover – a modification that accounts for this observer effect is needed when the vortex observations come from meteorological data taken at a landed station. Currently, the DDA theory used in e.g. Newman et al. (2019a) includes the effect of wind speed via the sensible heat flux (which is proportional to drag velocity), and – as discussed above – this effect may be overly strong because IR radiative heating (which does not involve drag velocity) is neglected. The good agreement (described above) between the DDA and vortices determined from surface meteorological data may therefore exist, in part, because the over-reliance on sensible heat flux brings in a greater wind speed dependence and partially compensates for this observer effect being ignored.

For consistency with previous work, in Sect. 9 we provide predictions of DDA for Jezero crater and selected other landing sites using the DDA formulation used in Newman et al. (2019a) and other papers. Going forward, however, we should consider the IR radiative heating and observer effect when comparing predictions with observations.

## Multi-model Predictions of Diurnal Cycles at the Landing Site and Season ($\text{Ls}\sim 5^{\circ}$)

**Fig. 2:**
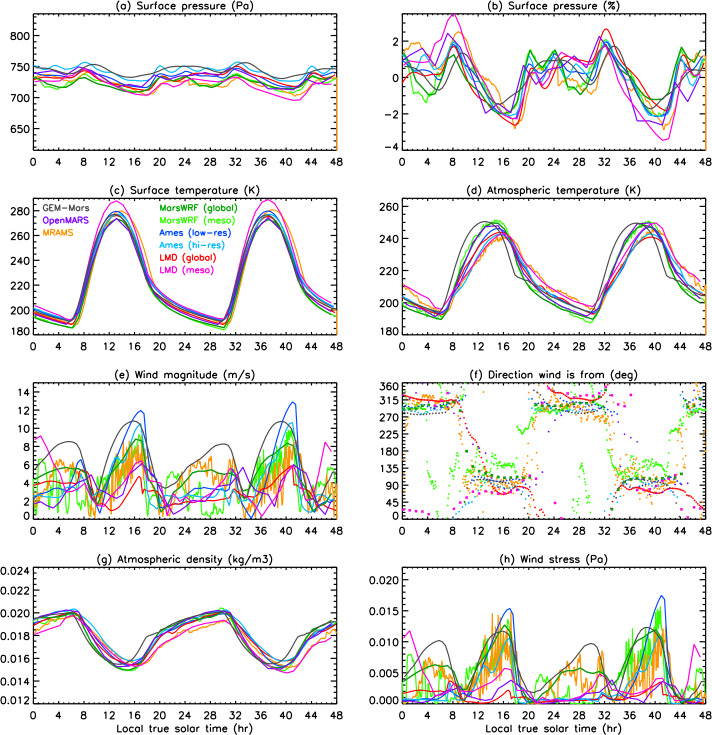
Predicted diurnal cycles from nine model simulations at $\text{Ls}\sim 5^{\circ}$. Shown are surface pressure (in Pa and as a percentage of the daily mean), surface and atmospheric temperature, wind speed, wind direction (shown as the direction from which the wind blows, in degrees clockwise from north, such that winds from the north – i.e. northerlies – have a direction of $0^{\circ}$, easterlies a direction of $90^{\circ}$, etc.), air density, and wind stress. Note that the same vertical axes are used for all figures showing diurnal cycles, for ease of comparison

Figure 2 shows the diurnal cycles of pressure, temperature, wind, and wind stress predicted in all nine model simulations at the landing site at the time of landing, $\text{Ls}\sim 5^{\circ}$ (early northern spring). In this section we interpret these results by examining the circulation drivers over the Jezero region at this time of year. We also examine the difference between the nine predictions, considering the effect on results of differences in simulation setup, such as different surface properties, dust distribution, grid spacing, etc.

### Diurnal Pressure Cycle

At this time in early northern spring the seasonal pressure cycle is approaching its secondary maximum (see Sect. 6.1) and surface pressures at the landing site are oscillating around $\sim740~\text{Pa}$ (Fig. 2a). These oscillations are due primarily to thermal tides, caused by the daily heating of the atmosphere and consequent motions that change the mass of atmosphere over any given point on the surface, and the interaction of these tides with Mars’s huge topography (e.g. Wilson and Hamilton 1996). The daily surface pressure cycle, seen most clearly in Fig. 2b which shows the pressure as a percentage of the daily mean, has three peaks per sol in the majority of simulations: at $\sim00{:}00$, $\sim08{:}00$, and $\sim20{:}00$ Local True Solar Time (LTST). The peak at $\sim08{:}00$ is present and has the highest amplitude in all simulations. The peak at $\sim00{:}00$ is most variable, occurring closer to $\sim01{:}00$ in the LMD simulations and appearing as more of an inflection in the OpenMARS simulation, which shows a clearer overnight peak at $\sim03{:}00$. The GEM-Mars simulation instead has only two daily peaks (with one broad peak spanning the $\sim20{:}00\text{--}03{:}00$ period). By contrast, the Ames high-resolution and MRAMS simulations have a fourth peak at $\sim03{:}30$ or $\sim02{:}30$, respectively, with some hint of this in the Ames low-resolution simulation also.

A large enhancement in the diurnal pressure range is observed in Gale Crater and is predicted by simulations that resolve its topography (Tyler and Barnes 2013; Wilson et al. 2017; Richardson and Newman 2018), as a result of hydrostatic adjustment due to the daily cycle of air temperature along a slope (Richardson and Newman 2018). However, the three pairs of high- and low-resolution simulations (Ames, LMD, and MarsWRF) show little consistent difference in their diurnal pressure range between the high- and low-resolution versions, despite only the former resolving the topography of Jezero crater. The reason is that this effect is far smaller in the shallow Jezero crater than in the much deeper Gale crater. Note too that, at this season, the near-resonant diurnal Kelvin wave has a big impact on the diurnal tide harmonic observed at the lander site, although not as strong as at $\text{Ls}=90^{\circ}$ (Wilson et al. 2017), which will also have a major influence on the diurnal pressure range in all simulations shown.

The highest-resolution simulations (mesoscale MarsWRF and MRAMS) also show small fluctuations of pressure on timescales of the output frequency (5 minutes). Even if the low-resolution simulations were output more frequently, such fluctuations would not be apparent, as they are due to smaller-scale perturbations not resolved by the low-resolution models. These may include anything from convective cells to gravity waves and are discussed in more detail in Pla-García et al. (2020, this journal). The mesoscale LMD simulation, despite having a slightly lower resolution (Table 1), likely also contains such features, but output was only available every half hour so this cannot be confirmed at present. Finally, there is significant sol-to-sol variability in most simulations. The mesoscale LMD simulation shows the most over the two sols shown here, possibly due to strong wave activity in the global LMD simulation that is amplified in the smaller-scale model. However, we acknowledge that two sols is inadequate to make a true assessment of the degree of variability in all simulations.

At this season, both MarsWRF simulations have a much stronger semi-diurnal vs. diurnal mode than in all other simulations (see Online Resource 2). This may be due to the fact that the vertical dust distribution, and also the day-night variation in dust, is directly imposed based on TES observations, whereas these factors are predicted by model transport in most other simulations. In MRAMS, the vertical dust distribution is imposed based on SPICAM observations, but there is no longitudinal or time-of-day dust variation. A more thorough exploration of the link between the 4-D dust distribution and predicted pressure cycles, including a detailed analysis of the migrating and non-migrating tidal harmonics over more sols of output, would be highly informative and assist in interpreting MEDA pressure observations. This is beyond the scope of the present work but will be examined in a future study.

In summary, aside from the lack of a third peak in one simulation and the presence of a fourth peak in two, and an enhanced semi-diurnal vs. diurnal mode in the MarsWRF simulations, the overall amplitude and shape of the pressure cycle is rather similar between all nine simulations. This may be due to the similar dust prescription at this time of year (Table 2 and Sect. 3.1), as dust abundance and distribution has a large impact on atmospheric tides (e.g. Leovy and Zurek 1979; Wilson and Hamilton 1996; Guzewich et al. 2013).

### Diurnal Temperature Cycles

The general shape of the diurnal cycles of surface and air temperature (respectively Figs. 2c and 2d) are similar between all nine simulations, but this is expected given that they are strongly controlled by the time variation of solar insolation. Looking more closely, there are some significant differences between the model predictions. The first is the difference in nighttime surface and atmospheric temperatures. The former varies by up to 10 K while the latter varies by more than 10 K across the nine simulations. One possible explanation is that this is due to differences in the surface thermal inertia assumed in each simulation at the landing site location, as a higher thermal inertia will result in a slower loss of heat from the surface after dusk, which will similarly enable the near-surface atmosphere to remain warmer for longer, while a low thermal inertia will result in a faster loss of heat from the surface at night and hence also faster cooling of the near-surface atmosphere. A comparison with Table 1 reveals that the simulations that are warmest and coldest at night (mesoscale LMD and both MarsWRF simulations, respectively) have very nearly the highest and lowest thermal inertias, respectively. However, the simulations with the highest and lowest thermal inertias (OpenMARS and MRAMS, respectively) both sit mid-pack. This suggests that differences in dust opacity and/or in how the models exchange heat between the subsurface, surface, and atmosphere are also involved. A more detailed investigation would be informative but is beyond the scope of the current study.

The second clear difference is the timing and magnitude of the afternoon peak in atmospheric temperature, despite afternoon surface temperatures being mostly very similar. This peak occurs relatively late (at $\sim15{:}30$ or 16:00) in most of the simulations, but at least an hour earlier in the OpenMARS and both MarsWRF simulations and at least two hours earlier in the GEM-Mars simulation. Also, the peak atmospheric temperatures reached vary from $\sim242~\text{K}$ in the global LMD and MRAMS simulations to $\sim250~\text{K}$ in the GEM-Mars, OpenMARS, and both MarsWRF simulations. Given that output is provided at 1.5 or 2 m altitude for the latter set of simulations, compared to 4.5, 5, or 14.5 m for the rest (Table 1), and given that the near-surface temperature decreases rapidly with height at this time of day, this spread is somewhat expected (see Sect. 3.8.2). However, there remains considerable sol-to-sol variation in peak atmospheric temperatures in several simulations, despite only 2 sols being shown. This may be due to changes in dust opacity and/or differences in planetary wave activity from sol to sol.

Finally, the mesoscale LMD simulation has by far the highest peak surface temperatures, more than 10 K warmer than the closest other prediction, yet its atmospheric temperatures are in company with those of the other simulations. The reason for this is unclear from the differences in surface and dust properties shown in Tables 1 and 2. Even more puzzling is the fact that the global LMD simulation – which drives this mesoscale simulation – has daytime surface temperatures similar to those of the other simulations.

### Diurnal Cycles of Wind Direction

Figure 2f shows the direction from which the wind blows as a function of time at $\text{Ls}\sim 5^{\circ}$ over two sols. There is a modest agreement across all nine simulations over much of each sol. Between $\sim22{:}00$ and shortly after sunrise ($\sim07{:}00$), predicted winds are almost entirely from between 270 and $340^{\circ}$ – i.e., range from westerlies to north-north-westerlies. Between $\sim10{:}30$ and 18:30 ($\sim\text{dusk}$), winds are almost entirely from between $\sim70$ and $135^{\circ}$ – i.e., range from east-north-easterlies to east-south-easterlies, the exception being high-resolution MarsWRF which has daytime winds from a south-easterly direction (see also Sect. 5.6). Winds rotate between these periods, and there is no clear sense of whether clockwise or anticlockwise rotation dominates the post-dawn and post-dusk rotations, with some simulations even showing a different rotation sense from one sol to another. The highest-resolution simulations, mesoscale MarsWRF and MRAMS, both show significant fluctuations in wind directions during the daytime and also at night, particularly in the case of the highest-resolution simulation (MarsWRF); see Sect. 5.6 for interpretation. However, the mean wind direction averaged over any two-hour period is still as described above.

This similarity of wind directions over the main daytime and nighttime periods, with changes in direction occurring shortly after dawn and dusk, is an indicator of strong control by flows associated with large slopes. As explained in e.g. Tyler et al. (2002) and Richardson and Newman (2018), daytime heating causes air to flow up slopes, while nighttime cooling causes air to flow down slopes. This pattern of wind directions is very similar to that observed by MSL in Gale crater, in which the slopes involved were the very large local slopes of Aeolis Mons in the crater center. The Mars 2020 landing site is again in a crater, now close to the crater rim. However, Jezero crater is far shallower than Gale crater and the slopes near the landing site are far shallower than those close to the landing site of MSL. In addition, the largely similar pattern of behavior between the high- and low-resolution simulations demonstrates that the slopes of Jezero crater itself cannot be responsible, as it is not resolved in the latter simulations. We must therefore look to more regional slopes for an explanation.

**Fig. 3:**
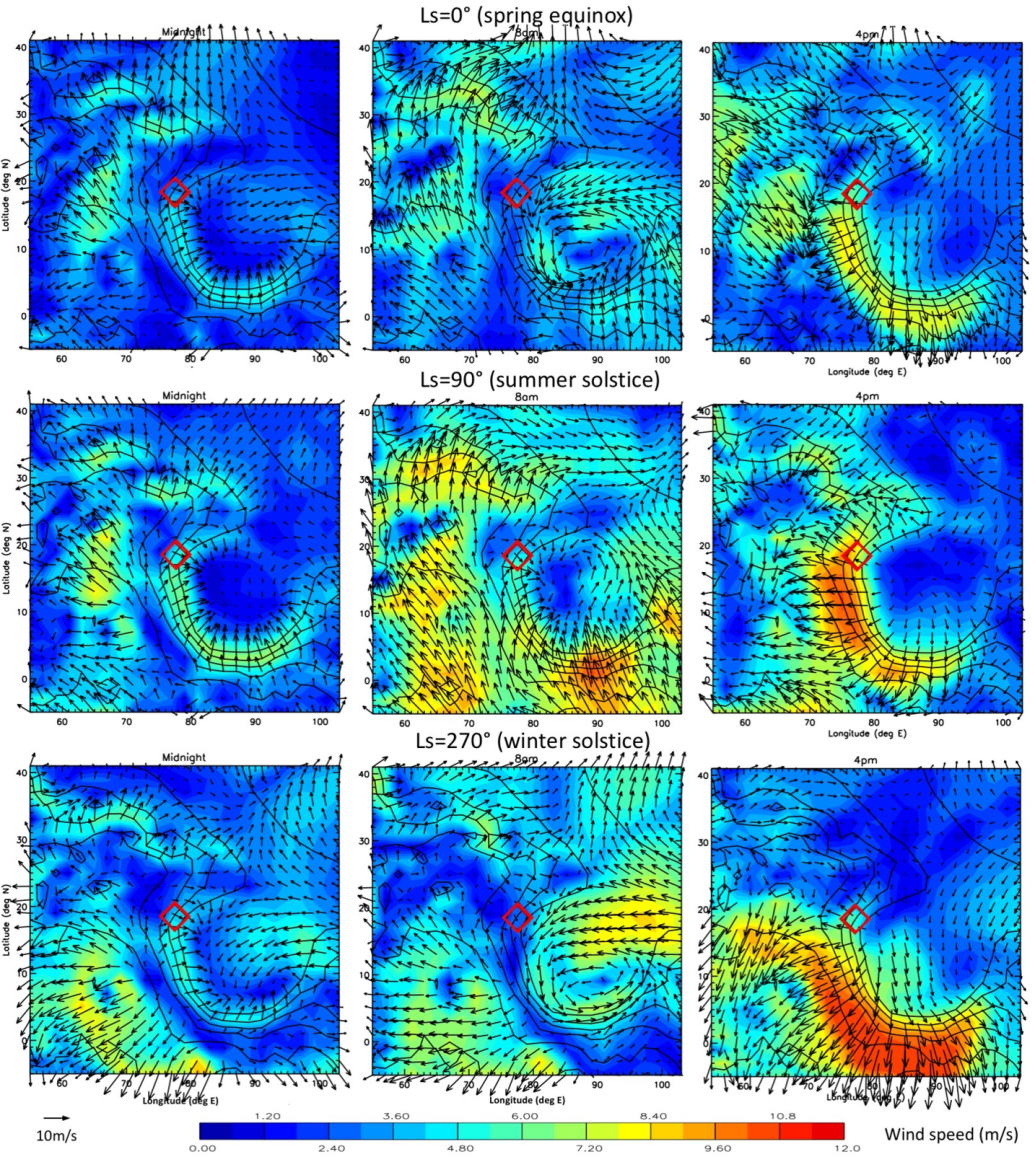
Snapshot of 1.5 m altitude winds (vectors) and wind magnitudes (shading) at three times of sol for $\text{Ls}=0^{\circ}$ (local spring equinox), $90^{\circ}$ (summer solstice) and $270^{\circ}$ (winter solstice) zoomed in on the Jezero/Isidis region in the global MarsWRF simulation. Times shown are local true solar time (LTST) at the center of the domain. Topography at 1 km intervals is shown as black contour lines. The red diamond is centered on the landing site coordinates and is sized to guide the eye without obscuring wind vectors

Figure 3 shows snapshots of winds at three times of sol and for three seasons from a portion of the global MarsWRF simulation, with the location of Jezero crater indicated by a red diamond. The topography is shown as black contours in Fig. 3 (and more clearly in Fig. 1b), and shows that Jezero crater sits on the NW rim of the large Isidis impact basin, which sits just north of the equator on the topographic dichotomy boundary, to the east of Syrtis Major. The top row of Fig. 3 shows MarsWRF predictions for roughly the landing season ($\text{Ls}\sim 0^{\circ}$). Looking at the time variability of wind direction inside the red diamond, it is clear that the slopes of Isidis basin control the bulk of the diurnal cycle seen in Fig. 2f. Nighttime winds flow down the local Isidis basin slope, from $\sim\text{NW}$ to SE (or WNW to ESE), as shown in the midnight plot. These flows continue through shortly after local sunrise, hence still describe much of the flow in the 8 am plot as well. By contrast, daytime winds flow up the local slope from $\sim\text{SE}$ to NW (or ESE to WNW), as shown in the 4 pm plot. Between these two flows, wind directions vary rapidly and wind speeds are generally very weak (not shown). The animation in Online Resource 3 shows the full diurnal cycle of global MarsWRF winds over multiple sols at this season, zoomed in on the same region, while Online Resource 4 shows the same for the GEM-Mars simulation.

Regional circulations are also influenced by the global mean meridional circulation and atmospheric tides, however. Around the Martian equinoxes ($\text{Ls}\sim 0$ and $180^{\circ}$), the global mean meridional circulation consists of two Hadley cells that rise roughly at the equator and descend at mid-latitudes in both hemispheres, with near-surface return flow toward the equator. In the northern hemisphere, one might therefore expect a general northerly background flow at this location ($\sim 18.5^{\circ}$ N) and season, but this is not readily apparent in the Fig. 3, $\text{Ls}=0^{\circ}$ results, thus it may be relatively weak and overcome by other controls. The final control on winds at Jezero is that by atmospheric tides, but determining their local impact is very difficult as they are a complicated function of location and season.

### Diurnal Cycles of Wind Speed

As shown in Fig. 2e, there is considerable variability in predicted wind speed between the simulations. While they all predict roughly two peaks in wind speed per sol, the exact timing of the morning peak differs greatly, as does the relative magnitude and shape of the two peaks. All models predict a late afternoon peak in wind speed at $\sim17{:}00$, corresponding to the peak in the daytime upslope flows on the NW slopes of Isidis basin. A strong upslope flow has been well-established for several hours by that time and there is a delay between air temperatures (which drive the upslope flow) decreasing and the flow starting to decrease. In general, the late afternoon has the strongest wind speeds over the entire sol, although there is significant sol-to-sol variability in when the strongest winds occur in the LMD global and (especially) mesoscale simulation results. The afternoon peak corresponds to ∼east-south-easterly, easterly, or east-north-easterly winds (Fig. 2f) in most simulations, except for the mesoscale MarsWRF simulation which predicts south-easterly winds at the time of peak wind speeds (see Sect. 5.3). However, peak wind speeds vary by more than a factor of two, with winds in the Ames low-resolution simulation exceeding $12~\text{ms}^{-1}$ while the peak LMD global winds are below $6~\text{ms}^{-1}$.

The overnight period varies even more between simulations. In most there are moderate winds overnight, which rise (sometimes abruptly) to a peak shortly before $\sim08{:}00$ before rapidly declining. This behavior is associated with the peak of the nighttime downslope flows on the Isidis basin slopes, which would be expected to increase from dusk through dawn. However, the MarsWRF global simulation shows nearly constant wind speeds from midnight to $\sim08{:}00$ in both sols. The four high-resolution simulations show more fluctuations in wind speeds overnight and also more sol-to-sol variability, all of which may be a result of their sensitivity to more variable local crater flows and/or to the nighttime turbulence they can resolve (see Sect. 5.6 and Online Resources 5, 6, and 7, which show wind animations from the mesoscale MarsWRF, mesoscale LMD, and MRAMS simulations, respectively). Nighttime wind speeds are even more variable between simulations, with speeds often below $2~\text{ms}^{-1}$ in mesoscale MarsWRF at night, but with speeds pre-dawn exceeding $8~\text{ms}^{-1}$ in GEM-Mars in both sols, and briefly peaking above $8~\text{ms}^{-1}$ in the mesoscale LMD simulation at midnight in some sols.

Most simulations predict a dip in wind speeds twice every sol, at the time when wind directions are rotating between the upslope and downslope directions ($\sim10{:}00$ and $\sim21{:}00$). The exception is the second sol of the OpenMARS simulation, which predicts a peak and unusual wind direction at around 10:00. This may be due to OpenMARS being an assimilation run, in which the model is very constrained by observations of temperature that may include strong wave activity, while the other simulations are either entirely “free-running” (GEM-Mars) or constrained by at most twice-daily observed dust opacity changes.

The impact of model resolution on wind results is discussed in Sect. 5.6. The boundary layer schemes and vertical grid resolutions used in each simulation are likely also a major cause of differences in wind predictions. Another possible source of differences between results is the method of extrapolating winds from the lowest model layer to 1.5 m assuming a log wind profile in most cases, as described in Sect. 3.8.1, rather than the simulations doing this extrapolation within their own boundary layer schemes in which the stability profiles are available. Investigating this is beyond the scope of this work, but each modeling group will likely consider this independently when ground truth wind measurements in Jezero crater become available.

### Diurnal Cycles of Atmospheric Density and Wind Stress

As shown in Fig. 2g, atmospheric density varies by almost 30% over the course of a sol on Mars. This is mostly due to the huge diurnal cycle in near-surface atmospheric temperature (which varies by a similar amount; Fig. 2d), rather than the diurnal cycle in surface pressure (which varies by only a few percent, as shown in Fig. 2b), which explains the similarity between the simulated densities despite the slight variations in the pressure cycles. Density peaks at $\sim06{:}00$ and has a minimum at $\sim15{:}00-16{:}00$ each sol. As described in Sect. 2.6, atmospheric density variations are often neglected in aeolian investigations, yet the linear dependence of wind stress on density (Eq. (1)) means that a 30% change may have a significant impact on the daily peak and integrated sand flux and direction. Even on Earth, typical diurnal variations in atmospheric temperature in desert regions can result in an atmospheric density variation of $\sim10\%$, and day-to-day variations of up to 30% have been recorded, thus it should probably not be ignored even for terrestrial studies.

Wind stress (Fig. 2h) depends on atmospheric density and drag velocity squared (Eq. (1)), thus differences between predictions of wind speed are heightened. In addition, while the timing of the afternoon peak in wind speed coincides with the time of lowest daily atmospheric density, the morning peak in wind speeds in most models coincides with the period of highest atmospheric density. As a result, if slightly stronger wind speeds are predicted during the morning (as in the first sol of the global LMD simulation) then significantly stronger wind stresses occur in the morning, when winds are from the northwest, than in the afternoon. In virtually all cases, however, simulations predict significantly stronger wind in the afternoon and the effects of squaring the wind speed are only partially offset by the lower atmospheric density, hence wind stress also peaks in afternoon, at a time when winds are from the east or southeast.

As for wind speed, there is a large spread in predicted wind stress across the nine simulations. In most cases here, however, either wind stress was output directly from the model or drag velocity was output and converted to wind stress using Eq. (1). Thus the issues with extrapolating should not be a factor. As such, the large spread in predicted wind stress is a direct consequence of differences between the models, the reasons for which are not clear. In this study we look at other seasons at the landing site location, but examining whether these differences extend planet-wide, and whether the results are consistent at different locations (e.g. Model A always predicts stronger wind stresses than Model B) is beyond the scope of this work. However, we note that drag velocity and wind stress are key parameters for the surface exchange of heat, momentum, and tracers (e.g. water vapor), and lifting or motion of dust and sand. Thus understanding these differences in predicted wind stress may be key to understanding why models differ in their predictions of the water cycle, dust storms, and more. See further discussion in Sect. 6.3.2.

### Influence of Resolution and Crater Topography on Wind Speed and Direction

We can compare the diurnal cycles of wind speed and direction in the high-resolution vs. low-resolution models to search for the impacts of local scale effects, in particular the local Jezero crater topography. It might be expected that higher-resolution models would predict the strongest peak wind speeds, as they are able to resolve smaller-scale flows (driven by waves or topography) that are effectively averaged out in the lower-resolution models. For example, the GEM-Mars grid spacing is such that one grid box covers the same area as $\sim6{,}000$ grid boxes in MarsWRF domain 5 (see Table 1). However, Fig. 2e shows that the Ames low-resolution simulation predicts stronger winds than the Ames high-resolution simulation, despite there being virtually no differences between these simulations except for horizontal resolution. Similarly, nighttime mesoscale MarsWRF winds are often far lower than those in the global MarsWRF model, although this is typically reversed during daytime. The reason is that while higher resolutions can capture stronger wind gusts they equally enable the local, low-level topographic component to interfere with the deeper, larger scale (planetary) component. If, for example, the large-scale flow is northerly but daytime upslope winds on local topography are southerly, the two flows will oppose each other and the wind speed will be reduced. Thus it is incorrect to assume that a low-resolution simulation will always predict lower wind speeds than a high-resolution simulation, or vice versa; the difference is typically very sensitive to the exact time, location, and resolution differences.

**Fig. 4:**
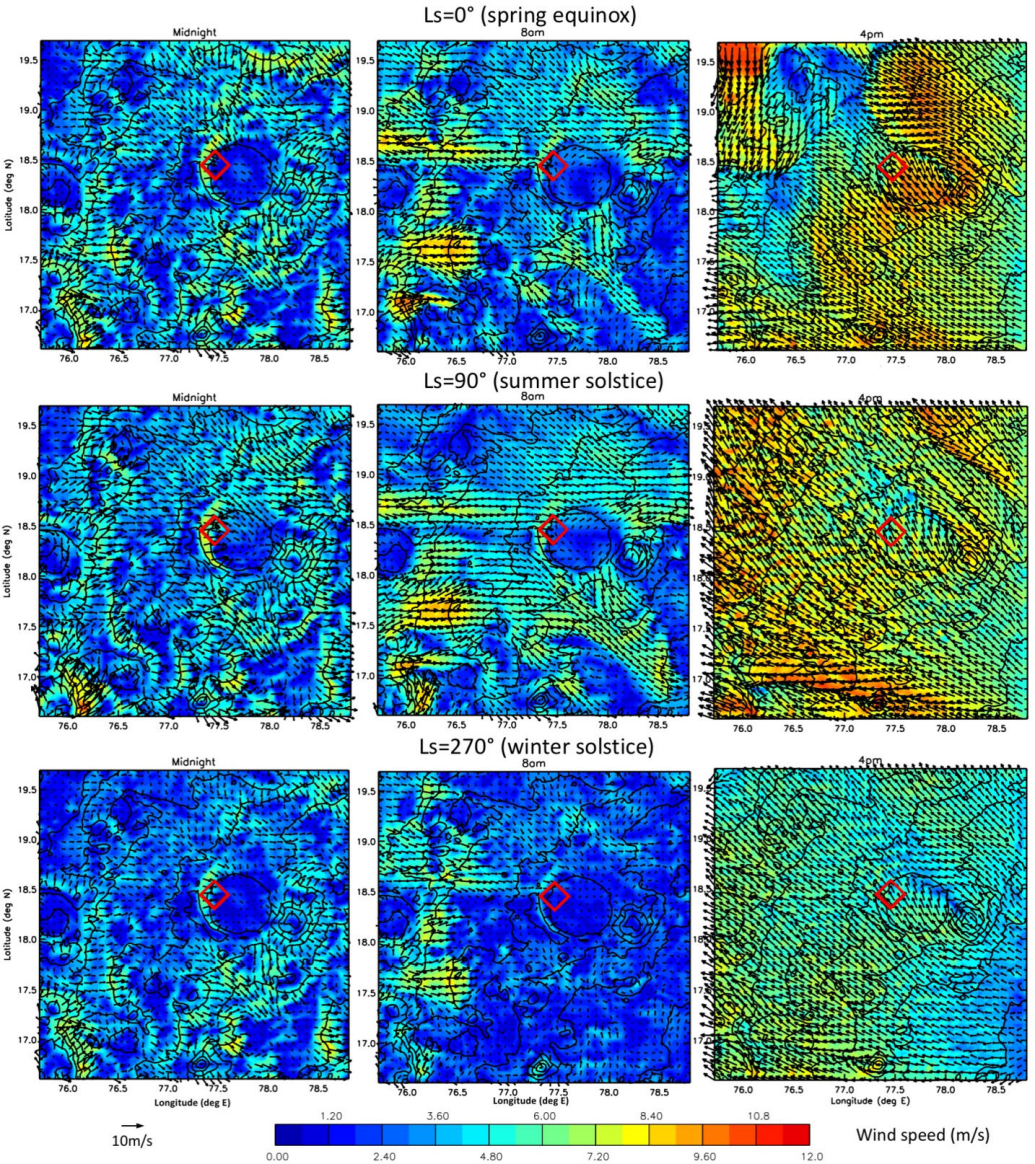
As in Fig. 3 but now showing winds at six times of sol in domain 5 of the mesoscale MarsWRF simulation for $\text{Ls}=0^{\circ}$ only, with every third wind vector shown

To study this further, we focus on results from the mesoscale MarsWRF simulation. Figure 4 shows a snapshot of mesoscale MarsWRF winds as a function of time of sol in three seasons from the innermost nest of the simulation (domain 5), while the animation in Online Resource 5 shows winds every 10 minutes over a full sol for $\text{Ls}\sim 5^{\circ}$ only. In domain 5, Jezero crater itself is well-resolved (note that for clarity only every third wind vector is plotted in Fig. 4), and the red diamonds again mark the landing site location. As shown in Fig. 1, the landing site is located both near the NW slope of Isidis basin and the NW slope of Jezero crater. This means that, to a large extent, slope flows associated with both the regional and local topography should operate in the same directions and reinforce rather than oppose each other. Indeed, looking at the top row of Fig. 4 showing $\text{Ls}=0^{\circ}$, it is clear that downslope winds on the WNW slopes of Jezero crater at midnight (top left panel) – and on other, similarly-oriented slopes in the domain – are enhanced compared to the surrounding north-westerly winds associated with the regional Isidis basin downslope flow. However, note that the landing site location is sufficiently far from the crater rim that these stronger winds do not always penetrate to it, as shown by the animation in Online Resource 5. Indeed, while Isidis basin’s large-scale downslope winds may be reinforced on the Jezero crater slopes, much of the remainder of the crater interior appears to be decoupled from the regional-scale circulation at night, due to the local-scale crater circulation. This explains the variability in nighttime wind speeds and directions predicted at the landing site in mesoscale MarsWRF (Fig. 2e and f), with that location sometimes experiencing strongly reinforced downslope winds from the $\sim\text{NW}$ and sometimes experiencing relatively weak winds from other directions. Similar behavior likely exists inside Gale crater, with nighttime downslope winds on the crater’s northern and western slopes only sometimes penetrating to MSL’s location (Baker et al. 2021).

Conversely, during the daytime the upslope south-easterly winds over much of this region appear to flow over Jezero and nearby slopes taking little notice of the local topography. As also shown in Online Resource 5, the primary cause of the daytime variations in wind speed and direction (Fig. 2e and f) is then strong afternoon convection, with convective cells advected across the crater. The daytime slope flows operate over a broader vertical region compared with the nighttime slope flows, which are typically confined close to the surface, thus may be less sensitive to small-scale topographic differences. However, a detailed comparison of the winds at the landing site compared with the Midway and NE Syrtis locations that the rover may visit (see Fig. 1c and Sect. 7) shows that daytime winds are slightly weaker at the landing site inside Jezero crater, compared with just to the west of the crater. This suggests that the regional-scale daytime upslope winds, from the E/SE, encounter opposition from upslope winds on the interior E/SE rim of Jezero crater before reaching the landing site, and are thus weakened slightly.

Finally, recall that the mesoscale MarsWRF simulation produces a stronger southerly daytime wind component than other mesoscale simulations for $\text{Ls}\sim5^{\circ}$ (see Sect. 5.3), and this is also true, albeit to a lesser degree, for $\text{Ls}\sim 180$ and $270^{\circ}$ (see Sect. 6). While global MarsWRF often has the most southerly daytime winds of the remaining simulations it still remains in family with them, whereas mesoscale MarsWRF is a clear outlier. Looking at Fig. 4’s 4 pm plots, we can see that, while wind vectors inside the red diamond have a slightly larger southerly component than many of the wind vectors outside the crater, the general background flow is already south-easterly (rather than east-south-easterly). In other words, this is not purely due to the Jezero crater topography but is at least partly due to differences in the larger-scale flow in the nested MarsWRF simulation, which has two-way feedbacks enabled such that each nest affects the flow in its parent domain (Fig. 1). Comparing the zoomed-in movie of global MarsWRF wind vectors in Online Resource 3 with a movie showing domain 2 of the nested MarsWRF simulation (Online Resource 8), we can see that flow directions at the scale of Isidis basin are also more southerly in the nested simulation than in the standalone global run. Indeed, re-plotting Fig. 2 but now using output from the global domain of the nested simulation (Online Resource 9), we find the d01 nested MarsWRF wind directions sit roughly midway between the global MarsWRF and mesoscale (d05 nested) MarsWRF predictions. This result suggests that small-scale flows in this region have a noticeable impact on the regional circulation too. Opposing this argument is the fact that the Ames high-resolution simulation (which also permits feedbacks between small-scale and larger-scale flows) predicts wind directions that are very similar to those in the Ames low-resolution simulation (see Fig. 2f). However, the Ames high-resolution simulation has a resolution that is $\sim4$ times lower than that of d05 MarsWRF, thus does not capture such fine-scale features. It is also possible that the very high resolutions used in mesoscale MarsWRF (and also MRAMS) push too far into the so-called “gray zone” (Wyngaard 2004) in which a model both resolves and parameterizes eddies, and that this is adversely affecting their predictions (more so in the case of mesoscale MarsWRF due to the two-way feedbacks, whereas MRAMS is forced by pre-existing output from a separate global model). This will be explored by running the nested MarsWRF model down to only d04 or d03 in the near future. It will also, of course, be very informative to see how these differing predictions of wind direction compare with MEDA wind observations in Jezero crater.

## Multi-model Predictions of Seasonal Variations at the Landing Site

**Fig. 5:**
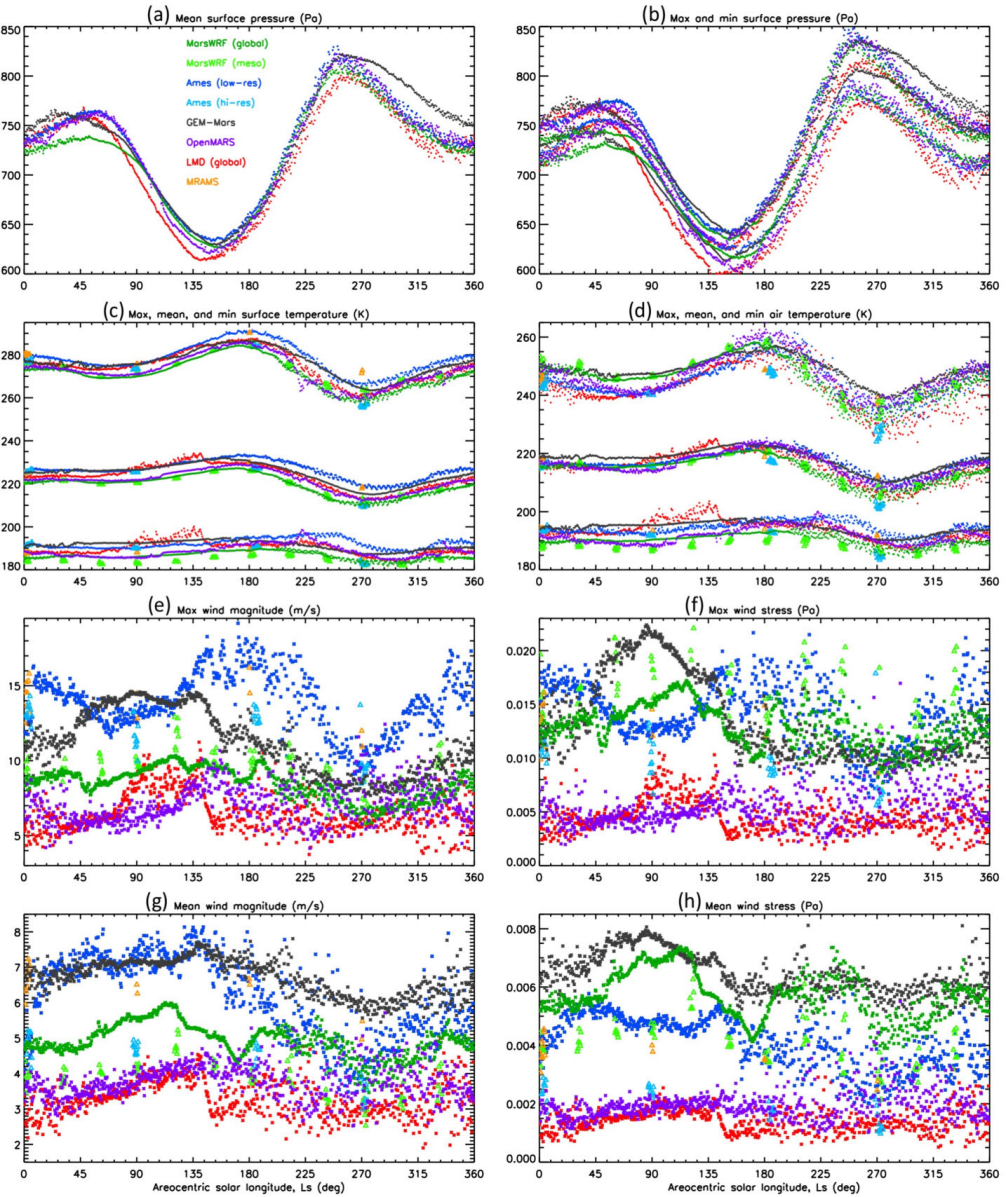
Predicted seasonal cycles of daily (**a**) mean surface pressure, (**b**) maximum and minimum surface pressure, (**c**) maximum, mean, and minimum surface temperature, (**d**) maximum, mean, and minimum atmospheric temperature at 1.5 m above the surface, (**e**) maximum wind speed at 1.5 m, (**f**) maximum wind stress at 1.5 m, (**g**) mean wind speed at 15 m, and (**h**) mean wind stress at 1.5 m, from eight of the simulations shown in Table 1

In this section we examine first the full seasonal cycle of meteorological variables then their diurnal cycles at three key seasons ($\text{Ls}=90^{\circ}$, $180^{\circ}$, and $270^{\circ}$) for comparison with the analysis of the landing season ($\text{Ls}\sim 5^{\circ}$) presented in Sect. 5. Figure 5 shows the predicted daily maximum, mean, and minimum in pressure, surface and atmospheric temperature, and also the maximum and mean wind speed and wind stress, at the landing site as a function of season. Seasonal variations in wind direction are more complicated to visualize so are assessed in other ways, as laid out in Sect. 6.3.3. In Fig. 5, results from the five low-resolution simulations are shown for all Ls, while results from three of the high-resolution simulations are shown for the Ls that were available at the time of publication: every $30^{\circ}$ of Ls for mesoscale MarsWRF and every $90^{\circ}$ of Ls for the high-resolution Ames and MRAMS simulations. Note that the Legacy Ames model simulation used to drive the MRAMS mesoscale model had not been tuned to provide a realistic pressure cycle, so MRAMS pressures are not shown in Fig. 5. For the high-resolution MarsWRF and Ames simulations, the daily max, mean, and min pressures are very similar to those of the parent (low-resolution) simulations, so are not included either.

### Seasonal Pressure Cycle

Figure 5a shows good agreement in the predicted seasonal pressure variation across all five global simulations. This is due to each model having been tuned (at some point) to match previously-observed pressure curves. Indeed, two of the models used in this work were re-tuned in the early stages of the study, prior to which they had timing offsets of up to $35^{\circ}$ in Ls and mean pressure offsets of up to 40 Pa, resulting in a pressure difference of over 100 Pa in some seasons. The seasonal pressure cycle at any location is produced by a combination of the CO_2_ cycle (in which the polar caps sublime and condense enough CO_2_ during spring/summer and fall/winter, respectively, to vary the mean surface pressure across Mars by about 30%) and atmospheric transport. The seasonal sublimation and condensation of CO_2_ at the polar caps is highly sensitive to the assumed cap properties, which include the albedo and emissivity of water ice and CO_2_ ice (e.g. Wood and Paige 1992; Guo et al. 2009) and also the depth of the sub-surface ice layer in models where this is allowed to affect surface thermal inertia (e.g. Haberle et al. 2008). To tune a model, these properties are typically adjusted until the simulated pressure curves from a global model, interpolated to the two Viking Lander sites, closely match those observed by Viking Landers 1 and 2. This tuning must be repeated for any significant changes to the model setup, parameterizations, or parameters that may impact the CO_2_ cycle. It is also possible to tune any pressure prediction by comparing with Viking pressure curves post-simulation (e.g. Forget et al. 2007). However, the benefit of tuning the model itself is that the CO_2_ cycle and global-scale pressure field inside the model are made more realistic during the simulation. Thus, all Mars global models will have been tuned at some point to match observed pressure curves, although this tuning may not have been repeated for the exact model setup used to produce the simulations presented in this work.

The minimum surface pressure at $\text{Ls}\sim 145^{\circ}$ occurs shortly after the large, condensing southern seasonal CO_2_ cap reaches its maximum extent, while the maximum at $\text{Ls}\sim 250^{\circ}$ (close to perihelion) occurs when the southern seasonal cap has nearly sublimed away and the smaller northern seasonal cap is still condensing. All simulations agree to within $10^{\circ}$ of Ls on the timing of the maxima and minima, with greater differences in the predicted surface pressure magnitudes in some seasons. The main outliers are the GEM-Mars simulation, which predicts slightly higher surface pressures during winter ($\text{Ls}=270\text{--}360^{\circ}$), and the MarsWRF global simulation, which predicts slightly lower surface pressures during spring ($\text{Ls}=0\text{--}90^{\circ}$). In both cases, this is due to the model’s parameters not having been retuned for the exact dust distribution used.

There is generally more sol-to-sol variability in the diurnal pressure cycle, seen as more scatter in the mean (Fig. 5a) and maximum and minimum (Fig. 5b) curves, through most of fall and winter through early spring. This is due to strong transient wave activity being present in northern high and mid-latitudes over most of this period, but almost absent during the summer (e.g. Lewis et al. 2016).

### Seasonal Temperature Cycles

Figures 5c and 5d also show a reasonable match in the predicted seasonal cycles of, respectively, surface and atmospheric temperatures across all simulations. Except for during the height of the dusty season ($\text{Ls}\sim 270^{\circ}$), the predicted maximum, mean, and minimum temperatures differ between simulations by under 10 K, with much closer agreement in many cases. All predict maximum daily max and mean surface and air temperatures at $\text{Ls}\sim 180^{\circ}$ (fall equinox), with a secondary maximum in max temperatures at $\text{Ls}\sim 360^{\circ}$, and predict minimum daily max and mean surface temperatures at $\text{Ls}\sim 270^{\circ}$ (winter solstice), with a secondary minimum in max temperatures at $\text{Ls}\sim 80^{\circ}$. Temperatures are thus lowest at local winter solstice but – far from peaking around local summer solstice – reach their warmest point a quarter of a year later. This is a result of both the landing site latitude, which is less than $20^{\circ}$ from the equator, and Mars’s eccentric orbit, which puts Mars at its furthest point from the Sun close to northern summer solstice. In winter, Mars is closer to the Sun but peak heating is at mid southern latitudes, hence Jezero crater does reach its coldest point. However, in northern summer, Mars is far from the Sun and peak heating is at mid northern latitudes, hence Jezero crater remains cool until equinox, when peak heating occurs at the equator.

At the height of the dusty season ($\text{Ls}\sim 270^{\circ}$) there is also more sol-to-sol variability within simulations, due to increased temporal variability in dust loading in this period. There is also more divergence between simulations, due to this being the period with the greatest spread in atmospheric dust distributions (see Sect. 3). During the daytime, more atmospheric dust will reduce the solar radiation reaching the surface and hence reduce surface and near-surface temperatures, which appears consistent with the lowest maximum atmospheric temperatures being predicted by the LMD global and high-resolution Ames simulations, which have the highest daily mean column dust opacities in this season (Table 2). However, note that the low-resolution Ames simulation predicts much warmer maximum temperatures, despite also having the highest mean dust opacity. Similarly, the MarsWRF simulations, which have the lowest daily mean dust opacities at this season, do not predict the highest maximum atmospheric temperatures; these occur in the GEM-Mars simulation, which has the next-lowest mean dust opacity. This lack of strict correlation between temperature and mean dust opacity is due to several factors: differences in surface properties between simulations (Table 1), the fact that the daytime dust opacity will be higher than the daily mean value in most of the simulations, and the difference in height at which atmospheric temperature is provided (Sect. 3.8.2), all of which should ideally be made identical across simulations in any future intercomparison studies.

A final point to note is that the global LMD model has elevated mean and minimum (nighttime) surface and air temperatures between $\text{Ls}\sim 90$ and $150^{\circ}$ due to water ice clouds in the low-latitude “aphelion cloud belt.” The latter is produced by upward transport of water-rich air (from the subliming northern cap) in the upwelling (northern) branch of the solsticial Hadley cell, combined with low condensation altitudes at low latitudes due to the cooler temperatures in this period. Unlike for dust, there is little signature in the maximum (daytime) temperatures, but nighttime temperatures increase due to radiative IR emissions from the clouds to the surface, which then heats the near-surface atmosphere (e.g. Wilson and Guzewich 2014). This effect is only visible in the global LMD simulation, although the GEM-Mars simulation also included cloud radiative effects. Such a strong signature of clouds in temperature data has not been seen at the low-latitude MSL landing site (e.g. Martínez et al. 2017), although small nighttime surface warmings attributed to clouds have been observed there (de la Torre-Juarez et al. 2019). Hence this prediction may be realistic and a function of the higher latitude of the Mars 2020 landing site than MSL, or may be the result of too much cloud abundance and/or heating in this global LMD simulation.

### Seasonal Cycles of Wind and Wind Stress

#### Mean and Maximum Daily Wind Speed

As shown in Fig. 5g, MRAMS and the Ames high-resolution simulation predict the strongest mean wind speeds at $\text{Ls}=0^{\circ}$, but results from those simulations are only available at four times of year, hence the true timing of their seasonal peak is unclear. Across the remaining six simulations, the predicted seasonality of mean wind speeds varies considerably. Four predict that mean daily wind speeds will peak some time in early-to-mid summer ($\text{Ls}\sim90\text{--}145^{\circ}$) and be lowest around winter solstice ($\text{Ls}=270^{\circ}$), whereas two (global LMD and OpenMARS) predict the strongest mean wind speeds will occur in winter, albeit with a large spread in daily mean wind speeds from sol to sol. Overall, however, the early-to-mid summer period contains the most consistently large mean wind speeds in all six simulations.

Wind speeds peaking in northern summer runs counter to the frequent description of northern winter as being the “windy” season due to the stronger mean meridional circulation at this time of year and its association with dust raising and storm events. However, recall from Sect. 5.3 that winds at the landing site location are strongly controlled by the regional Isidis basin topography. The result of combining regional topographic and global-scale flows is shown for the MarsWRF global simulation in Fig. 3, which shows near-surface wind predictions for northern spring equinox ($\text{Ls}=0^{\circ}$), summer solstice ($\text{Ls}=90^{\circ}$), and winter solstice ($\text{Ls}=270^{\circ}$). The equinoctial circulation was described in Sect. 5.3. At both solstices, the global mean meridional circulation consists of a single, more powerful Hadley cell that rises in summer mid-latitudes and descends in winter mid-latitudes, with near-surface flow across the equator from the winter to the summer hemisphere. Due primarily to the raised southern hemisphere topography compared to the north, in combination with increased solar forcing near perihelion and feedbacks between winds and dust lifting that result in increased dust loading, the $\text{Ls}=270^{\circ}$ solsticial cell is stronger than that at $\text{Ls}=90^{\circ}$ (e.g. Richardson and Wilson 2002). Overall, and for the Mars 2020 landing site at $\sim 18.5^{\circ}$ N, this means that: at $\text{Ls}=0^{\circ}$, the global mean background flow is weakly from the north; at $\text{Ls}=90^{\circ}$, it is moderately strongly from the south; and at $\text{Ls}=270^{\circ}$, it is very strongly from the north. Comparing the 4 pm plots in Fig. 3, and looking first at the very strong winds on the southern rim of Isidis basin, it is clear that these winds are strongest for $\text{Ls}=270^{\circ}$, when they are enhanced by the strong global mean winds from the north, but are weakest for $\text{Ls}=90^{\circ}$, when they are opposed by the moderately strong global mean winds from the south, with $\text{Ls}=0^{\circ}$ being slightly stronger overall than $\text{Ls}=90^{\circ}$. However, on the *northwest* slopes of the Isidis Basin, the situation is quite different, as the strong daytime upslope winds now come from the $\sim\text{ESE/SE}$. Hence for the region where Jezero crater sits, shown by the red diamond, and in the global MarsWRF model, the global mean circulation acts to *strengthen* these daytime upslope winds at $\text{Ls}=90^{\circ}$ and *weaken* them at $\text{Ls}=270^{\circ}$.

Note that one might expect this effect to reverse at night, when the slope winds are from the NW, hence the global mean circulation should strengthen them at $\text{Ls}=270^{\circ}$ and weaken them at $\text{Ls}=90^{\circ}$. However, nighttime winds at $\text{Ls}=270^{\circ}$ are as weak or weaker than at $\text{Ls}=90^{\circ}$ in nearly all simulations (see Sect. 6.6.2). We discuss this further in Sects. 6.4–6.6 where we look at the diurnal cycles of wind speed at $\text{Ls}=90^{\circ}$, 180, and $270^{\circ}$, respectively.

The timing of daily maximum wind speeds shown in Fig. 5e varies hugely between simulations. In the GEM-Mars, OpenMARS, and both MarsWRF simulations, peak wind speeds are roughly aligned with the peak in the mean (although global MarsWRF has a very large secondary maximum at $\text{Ls}\sim 190^{\circ}$). By contrast, in the low-resolution Ames simulation, peak wind speeds occur at $\text{Ls}\sim 180^{\circ}$, well after the peak in mean wind speed values, with a large secondary maximum in wind speeds at $\text{Ls}\sim0^{\circ}$. And interestingly, the LMD global simulation predicts peak wind speeds in mid-summer, despite daily mean winds peaking in mid-winter, although there are secondary maxima in peak winds at the latter time of year and also in early summer.

Also inconsistent across simulations, as discussed in Sect. 5.4, are the wind speed magnitudes. Daily mean wind speeds over the full Mars year vary from between $\sim2$ and $4.75~\text{ms}^{-1}$ in the global LMD simulation to between $\sim3.75$ and $8.25~\text{ms}^{-1}$ in the low-resolution Ames simulation. Daily maximum wind speeds differ even more, varying from between $\sim4$ and $11~\text{ms}^{-1}$ in the global LMD simulation to between 9 and $19~\text{ms}^{-1}$ in the low-resolution Ames simulation.

#### Wind Stress

The variation of wind stress with season (shown in Figs. 5f and 5h) is qualitatively similar to the variation of wind speed with season, suggesting that the seasonal variation in atmospheric density does not cause the timing of peaks in mean of maximum wind stress to differ significantly from the timing of peaks in mean or maximum wind speed. There is, however, a shift in relative magnitudes. Put simply, some of the simulations appear to get more of a “bump” than others when converting from wind speed to wind stress. (It should be noted that wind stress was output in addition to wind speed for most simulations, hence generally accounts for different stability conditions at that time in the model – see Eq. (3) – the exception being the global LMD simulation for which it was calculated from the wind speed at the lowest model height via Eqs. (1) and (4).) At $\text{Ls}\sim 90^{\circ}$ for example, the ordering of simulations in terms of mean wind speeds (from lowest to highest) is: LMD & OpenMARS, mesoscale MarsWRF, high-resolution Ames, global MarsWRF, MRAMS, then low-resolution Ames & GEM-Mars. Yet the ordering in terms of mean wind *stresses* is: LMD & OpenMARS, high-resolution Ames, MRAMS, mesoscale MarsWRF & low-resolution Ames, global MarsWRF, then GEM-Mars. Considering instead maximum winds and wind stresses: both MarsWRF simulations predict much lower maximum wind speeds at $\text{Ls}\sim 90^{\circ}$ than MRAMS, low-resolution Ames, and GEM-Mars (which are all quite similar); yet MarsWRF maximum wind stresses are greater than those for MRAMS and low-resolution Ames, while those in GEM-Mars are far greater.

**Fig. 6:**
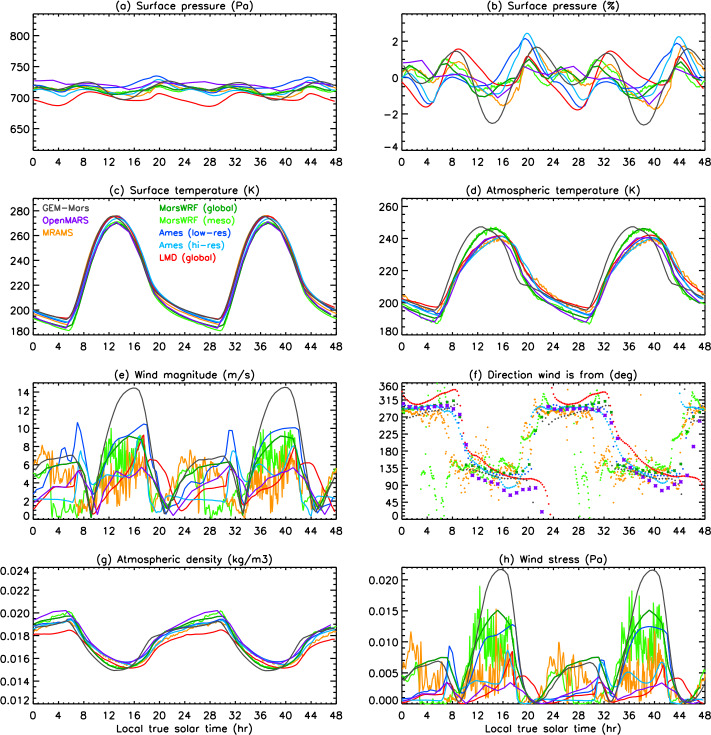
As in Fig. 2 but now for $\text{Ls}=90^{\circ}$ (northern summer solstice)

A possible explanation is that the strongest winds in one simulation occur at a different time of sol (with a much higher or lower atmospheric density) than the strongest winds in another. In combination with the amplification of wind speed differences (via the $u_{*}^{2}$ term in Eq. (1)), this likely explains some of the differences. However, inspection of the diurnal cycles of wind speeds and atmospheric densities at $\text{Ls}=90^{\circ}$ (see Fig. 6) confirms that this does not explain everything. Another possible explanation is the difference in roughness heights between the simulations, as shown in Table 1. The Ames and GEM-Mars simulations both use $z_{0}$ of $\sim1~\text{cm}$, which is about a third of that used in the MarsWRF and MRAMS simulations ($\sim3~\text{cm}$), due to their use of different surface roughness maps in these models. From Eq. (4), and assuming the same value of wind speed at 1.5 m in both pairs of simulations, this would result in the Ames and GEM-Mars simulations having $u^{*}$ about 80% lower than in MarsWRF and MRAMS, and consequently wind stresses about 60% lower. This likely explains the big “bump” experienced by the MarsWRF simulations when converting from wind speed to wind stress, compared to most other simulations. However, it does not explain why the MRAMS simulation does not see a similar bump, nor does it explain the large Ames/GEM-Mars difference here.

Given the differences in how 1.5 m wind speed is estimated for each simulation (Sect. 3.8.1) and the fact that wind stress is typically calculated using $u^{*}$ directly from the models, it appears that the apparent mismatch between relative wind speed and wind stress magnitudes may be a result of either our extrapolation method or the physics connecting $u^{*}$ and the near-surface wind profile in the models themselves. For example, while $z_{0}$ relates to the expected impact of surface elements on mixing, if one model generates more thermal instability than another during the daytime, this thermal effect will also mix more momentum down to the surface and result in greater wind stress. Such ideas should be investigated further in future.

#### Wind Direction

Daily mean wind direction can in principle be calculated either in terms of the frequency at which winds come from each direction or by weighting by wind speed. In either case, the meaning is very difficult to interpret, especially if (as shown in Fig. 2f) the two primary wind directions in a given sol differ by $\sim180^{\circ}$. We therefore examine diurnal cycles of wind speed and direction in key seasons instead (Sects. 6.4.2, 6.5.2, and 6.6.2). In addition, we consider the aggregate effect of varying wind speeds and directions on predicted aeolian transport by providing the direction of net sand transport for different wind stress thresholds (Sect. 8.1).

### Diurnal Cycles at $\text{Ls}=90^{\circ}$

To understand the changing circulation with season, it is necessary to go beyond daily maximum and minimum values. For pressure, this provides information on thermal tides; for winds, this allows maximum wind speeds to be correlated with time of sol and wind direction. Section 6.4 to 6.6 therefore examine the diurnal cycles at key seasons over the course of a Mars year. Note that diurnal cycles from the mesoscale LMD simulations are not available outside of the landing season, thus only eight simulations are compared here.

#### Surface Pressure at $\text{Ls}=90^{\circ}$

**Fig. 7:**
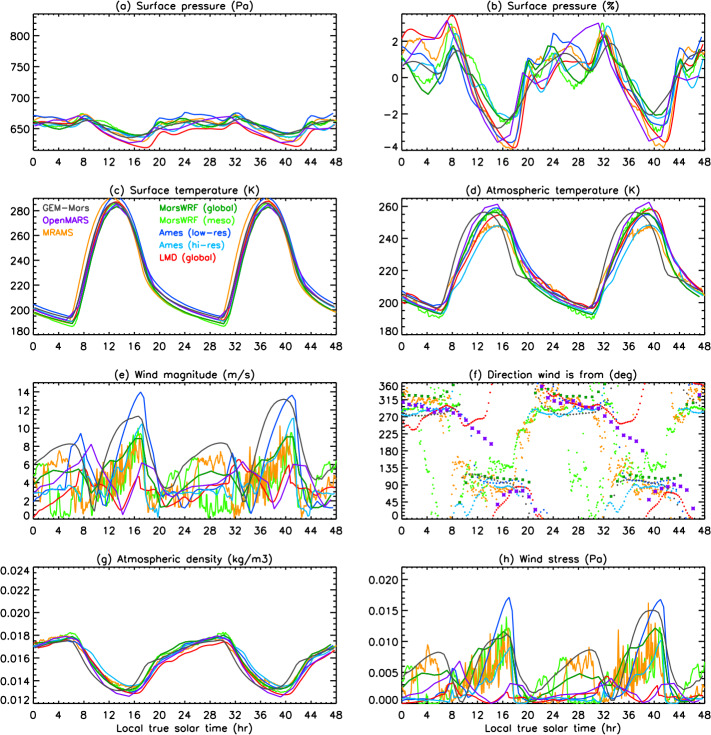
As in Fig. 2 but now for $\text{Ls}=180^{\circ}$ (northern fall equinox)

**Fig. 8:**
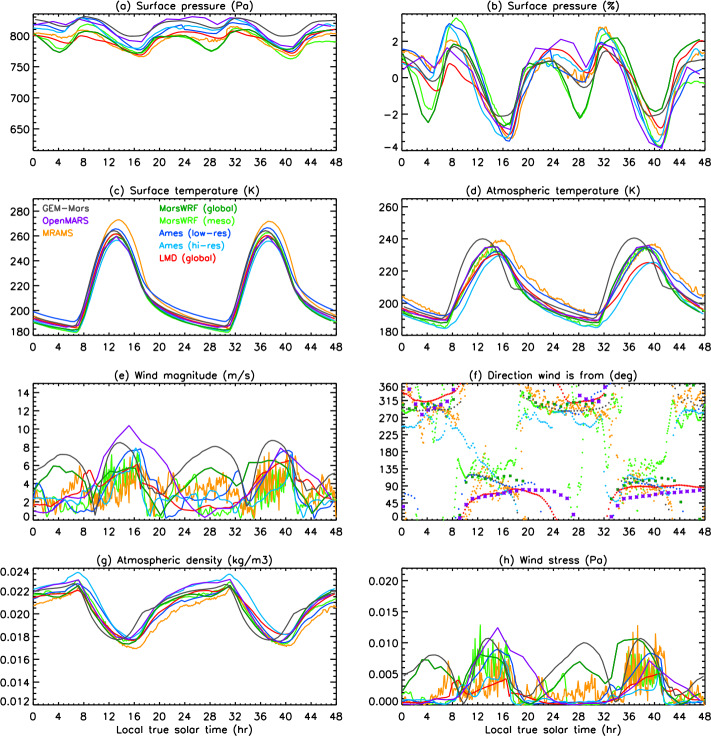
As in Fig. 2 but now for $\text{Ls}=270^{\circ}$ (northern winter solstice)

Figure 6 shows diurnal cycles as in Fig. 2, but now for northern summer solstice. Some key differences to the landing season are the shape of the diurnal pressure cycle, which has a weaker diurnal range than in other seasons in most simulations (compare Fig. 6b with Figs. 2b, 7b, and 8b). In the MarsWRF model, this is a result of the diurnal and semi-diurnal pressure tides being especially weak in this region of Mars in this season (Guzewich et al. 2016), and the diurnal and semi-diurnal amplitudes are lowest in this season in most simulations (see Online Resource 2). The main exception is GEM-Mars, which predicts a slightly larger diurnal pressure range in this season than at $\text{Ls}\sim 5^{\circ}$. Given the similarity between the column dust opacities in e.g. MarsWRF and GEM-Mars at $\text{Ls}=90^{\circ}$ shown in Table 2 (0.19 vs. 0.18), the difference in predicted pressure cycles may be due to differences in the vertical (or possibly also horizontal) distribution of atmospheric dust, which is prescribed according to TES nadir and limb observations in MarsWRF but determined self-consistently via parameterized dust lifting and transport in GEM-Mars. Alternatively, it may be due to differences in horizontal diffusion or damping in the models, either explicitly added or a result of the dynamical solver. Such differences are typically hard to tease out and not often considered when comparing model output with observations or other model output, but can have a surprisingly large impact on the pressure cycle. As an example, the midnight peak in MarsWRF simulations for $\text{Ls}\sim 5^{\circ}$ (Fig. 2a, b) only appeared when a horizontal momentum damping parameter inside MarsWRF was reduced by 75%. Indeed, the erroneously large size of this parameter in previous work, in addition to the simple dust prescription used, was a major reason why MarsWRF predictions of the pressure cycle inside Gale crater lacked the higher harmonics observed (see e.g. Fig. 15 of Newman et al. 2017).

At $\text{Ls}\sim 90^{\circ}$, most of the simulations predict a three-peak structure (with peaks at $\sim 01{:}30$, $\sim08{:}00$, and $\sim20{:}00$) while the global LMD and GEM-Mars simulations predict only the $\sim08{:}00$ and $\sim20{:}00$ peaks. Comparing these predictions to observations in this season should be a very good test of which simulations capture thermal tides well in the Jezero region and provide insight into why this is.

#### Wind and Wind Stress at $\text{Ls}=90^{\circ}$

As also shown in Figs. 5e and g, for most simulations this time of year has some of the strongest winds of the four seasons we are considering in detail. The main exceptions are the MRAMS and two Ames simulations, which predict overall stronger winds at both $\text{Ls}\sim 5$ and $180^{\circ}$. The main difference to the landing site season (Fig. 2e) is the relative size of the morning and afternoon peaks, and this differs between simulations. At $\text{Ls}\sim 5^{\circ}$, the afternoon peak was dominant in most simulations. At $\text{Ls}\sim 90^{\circ}$, GEM-Mars shows an even stronger afternoon peak relative to the morning one, whereas the Ames and MRAMS simulations produce the opposite behavior, with a morning peak that is now the same strength (albeit far briefer in the global Ames case) as the afternoon one. In addition, at summer solstice the three high-resolution models now predict a more sustained period of strong winds from late morning through sunset, whereas at spring equinox they predicted instead a large increase over that period to a peak at $\sim17{:}00$. The same holds true for wind stress (and hence associated aeolian transport).

Many of these changes are due to the southerly Hadley cell winds that blow across the Jezero region in northern summer, as described in Sect. 6.3.1. Looking at Fig. 3, showing global MarsWRF winds, there is a clear increase in southerly wind components at $\text{Ls}=90^{\circ}$ vs. the other seasons presented. In particular, strong southerly flows to the west of Isidis basin develop overnight and are still strong in the plot for 8 am. In some simulations (both Ames, global LMD, and OpenMARS), these merge with the strong nighttime downslope flows on the western slopes of Isidis basin to produce very strong downslope winds from the $\sim\text{NW}$ right around sunrise, as shown in Fig. 6e and in an animation of low-resolution Ames simulation output in Online Resource 10. In other simulations (GEM-Mars and global MarsWRF), the nighttime downslope flows on Isidis build gradually overnight but appear largely decoupled from the strong southerly winds (see the $\text{Ls}=90^{\circ}$, 8 am plot in Fig. 3, showing global MarsWRF results) with no pronounced morning peak around sunrise (Fig. 6e). Finally, in the two highest-resolution simulations (MRAMS and mesoscale MarsWRF), strong winds occur at night on the western slopes of Jezero crater, due to the Isidis basin downslope flows being reinforced by local downslope flows here. However, these appear not to penetrate far enough into the crater to reach the landing site coordinates for much of the night (see the $\text{Ls}=90^{\circ}$, 8 am plot in Fig. 4, showing mesoscale MarsWRF results), resulting in low wind speeds and lots of variability in wind direction around sunrise in both simulations (Fig. 6e). See discussion in Sect. 5.6.

Except for mesoscale MarsWRF, which already has a larger southerly component at $\text{Ls}\sim 5^{\circ}$, daytime wind directions show a stronger southerly component than at spring equinox, typically ranging between $\sim110\text{--}160^{\circ}$ (rather than $\sim70\text{--}135^{\circ}$ as at $\text{Ls}\sim 5^{\circ}$). This is due to the constructive combination of daytime upslope winds (from the SE or ESE) and background southerly flow associated with the Hadley circulation.

Finally, we note that daily maximum wind stresses occur earlier (at $\sim11{:}00$) in MarsWRF than in all other simulations, putting peak sand transport at a time of sol when winds (in all simulations) have a larger southerly component than later in the afternoon. Given the proximity to the season of peak MarsWRF wind stresses (Fig. 5f), this behavior is likely why the mesoscale MarsWRF simulation predicts a more southerly trend in sand transport than other models, as discussed in Sect. 8.

### Diurnal Cycles at $\text{Ls}=180^{\circ}$

Figure 7 shows diurnal cycles as in Fig. 2, but now for northern fall equinox.

#### Surface Pressure at $\text{Ls}=180^{\circ}$

The behavior in pressure is rather similar to that at the opposite equinox (i.e. the landing season) shown in Fig. 2b, other than the mean surface pressure being lower and the peak in pressure in the $\sim23{:}00\text{--}03{:}00$ period being stronger. This is linked to a much stronger diurnal amplitude at this time of year in all but the Ames high-resolution simulation (see Online Resource 2). There is substantial agreement in the shape of the pressure curves between simulations, although there is some spread in the diurnal pressure range, which varies from $\sim4\%$ (GEM-Mars and global MarsWRF) to over 7% (global LMD) of the mean. This is not correlated with dust opacity but may be linked to the 3-D dust distribution; however, there is also considerable sol-to-sol variability in pressure for most simulations, so the differences seen here in only two sols of output may not be significant.

#### Wind and Wind Stress at $\text{Ls}=180^{\circ}$

Diurnal cycles of wind speed, direction, and stress are also quite similar to those at the opposite equinox in most simulations. The main differences are stronger winds and wind stresses at most times of sol at this equinox in the low-resolution Ames simulation, potentially stronger morning winds and wind stresses in the OpenMARS and global LMD simulations (although the former assimilation run has too much sol-to-sol variability to be certain), and changes to the wind directions predicted by the OpenMARS and global LMD simulations. In the hours around noon, OpenMARS predicts a period of south-westerly winds then abrupt switch to north-easterlies, while global LMD predicts a slow rotation through northerlies, whereas all other simulations have already rotated to ∼easterly/south-easterly winds by noon.

### Diurnal Cycles at $\text{Ls}=270^{\circ}$

Figure 8 shows diurnal cycles as in Fig. 2, but now for northern winter solstice.

#### Surface Pressure at $\text{Ls}=270^{\circ}$

Unlike other times of year, the timing of peaks is nearly identical across all simulations around winter solstice, with peaks at $\sim08{:}00$ and $\sim23{:}00$, and no other significant peaks in the daily cycle. This is likely due to the increased dust loading in this season compared to the other three seasons examined (see e.g. the column dust opacities in Table 2). The semidiurnal amplitude is strongest at this time of year (compared to the other three times examined) in most simulations (see Online Resource 2). The exceptions are GEM-Mars, for which it is stronger at $\text{Ls}=90^{\circ}$, and the high-resolution Ames simulation, for which it is actually weakest at this time of year. In both MarsWRF simulations, the semidiurnal amplitude is at least three times the diurnal amplitude, resulting in two relatively smooth peaks of similar magnitude. GEM-Mars is the only other simulation to have a stronger semidiurnal than diurnal tide in this season, suggesting that GEM-Mars and especially MarsWRF have more vertically-extended dust heating than other simulations at this time of year, which are known to excite a stronger migrating semidiurnal tide (e.g. Guzewich et al. 2013). A detailed examination of the 3-D dust distribution and the associated excitation of migrating and non-migrating tides in each simulation would be needed to fully explore this, however.

#### Wind and Wind Stress at $\text{Ls}=270^{\circ}$

Wind directions are again similar to those at $\text{Ls}\sim 5^{\circ}$, but in the majority of simulations wind speeds and stresses are weaker. In this season, the general circulation produces strong northerly winds, which oppose the direction of the strong daytime upslope flows associated with Isidis basin (which generally dominate the daytime wind direction over the whole of Jezero crater, as demonstrated by the 4 pm plots in Fig. 4). The interference between these flows thus leads to generally weaker daytime winds, as shown in Fig. 4 (bottom right panel) and Figs. 8e and h. Despite this effect, however, we can see that the OpenMARS simulation has stronger peak winds and wind stresses than in the other seasons examined here. This is in agreement with Fig. 5e and g, which show that OpenMARS has its strongest mean and maximum wind speeds and stresses in this season. This may be a result of near-surface flows being more strongly coupled to higher-altitude winds than in other simulations, but a full examination is beyond the scope of this study.

### Summary of Predicted Seasonal Variations in Wind at the Landing Site

Based on predictions from eight atmospheric model simulations: We expect the winds observed at the Mars 2020 landing site to be controlled largely by slope winds on the NW slopes of Isidis basin, modified by the global mean circulation which enhances upslope winds over most of the daytime period in local summer. The greatest differences in wind patterns occur around local summer solstice ($\text{Ls}\sim 90^{\circ}$), when the global circulation creates strong southerly near-surface winds at low latitudes. This serves to reinforce the already-strong daytime upslope flows on NW Isidis basin from the east-south-east or south-east and strengthens winds earlier in the afternoon than in other seasons. As a result, nearly all simulations predict a more southerly daytime wind direction at $\text{Ls}\sim 90^{\circ}$ and most also predict the strongest wind speeds and stresses at that or nearby times of year, although two simulations predict peak daily mean wind speeds in winter instead, and one of those simulations predicts peak daily maximum wind speeds then. This has a profound impact on aeolian predictions at Jezero, as presented in Sect. 8.

We also expect some smaller modifications and local variations (e.g. from the landing site to Midway) due to local topography (especially the Jezero crater slopes and rim), but for these to primarily occur at times of sol when the strong Isidis basin upslope winds do not dominate over the region. For example, despite Isidis basin and Jezero crater nighttime downslope winds reinforcing on Jezero’s western rim, the highest-resolution simulations predict more variable and overall weaker nighttime winds at the landing site, due to those winds not always penetrating that far into the crater and the crater topography itself seeming to block the large-scale flow. These predictions are explored further in the next section.

## Expected Variations in Circulation Across the Mars 2020 Mission

**Fig. 9:**
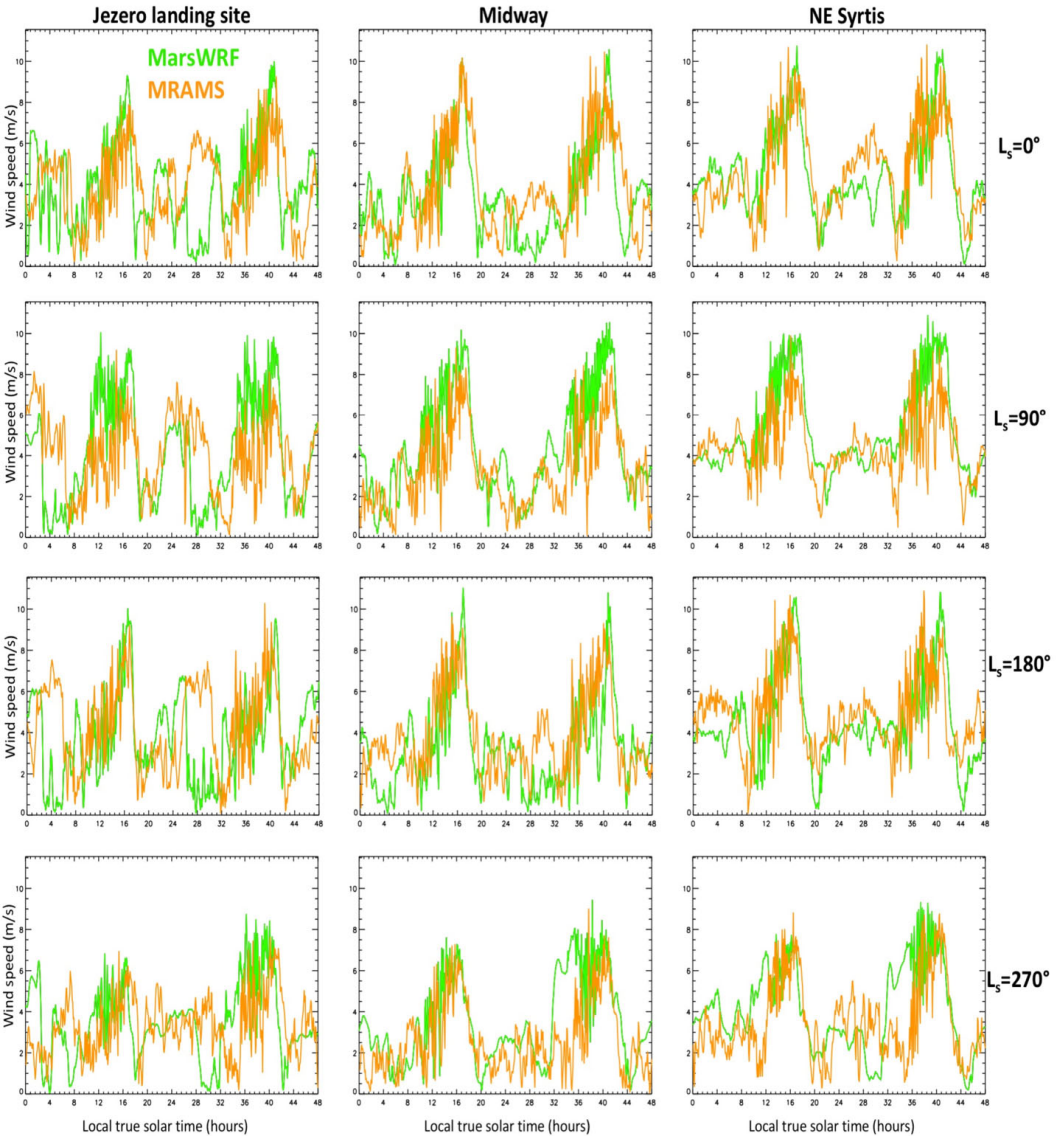
Predicted 1.5 m wind speeds for $\text{Ls}=0$, 90, 180, and $270^{\circ}$ at the landing site, Midway, and NE Syrtis locations, in the mesoscale MarsWRF and MRAMS simulations

**Fig. 10:**
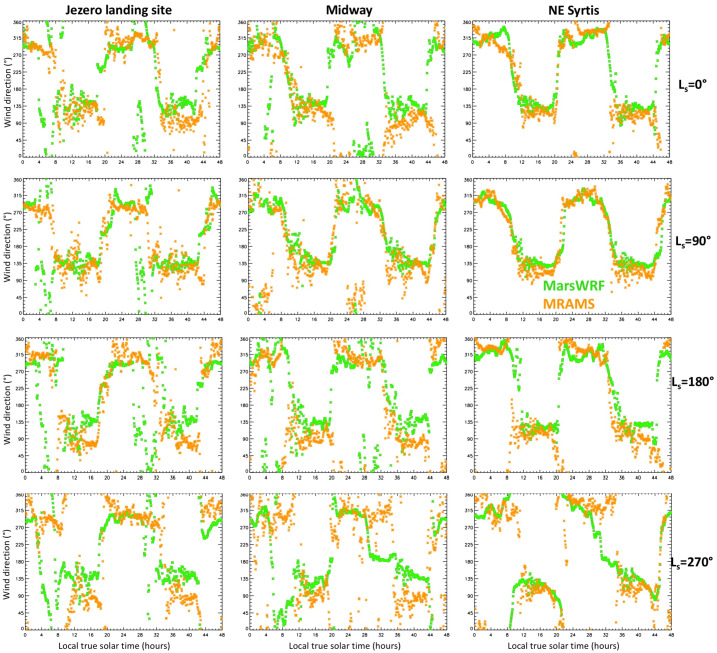
As in Fig. 9 but now showing predicted 1.5 m wind directions

In this section we use the two highest-resolution simulations, mesoscale MarsWRF and MRAMS, to examine differences that may occur along the rover traverse, focusing on the Midway ($77.05~^{\circ}\text{E}$, $18.27~^{\circ}\text{N}$) and NE Syrtis ($77.16~^{\circ}\text{E}$, $17.89~^{\circ}\text{N}$) locations that may potentially be visited during an extended Mars 2020 mission. Their locations are shown in Fig. 1b. Figures 9 and 10 show respectively wind speeds and directions over 2 sols for the landing site, Midway, and NE Syrtis, for $\text{Ls}=0$, 90, 180, and $270^{\circ}$ in the two simulations. As discussed in Sects. 5 and 6, these simulations differ in some aspects at the landing site. For example, there are some periods (e.g. pre-dawn in most seasons) when mesoscale MarsWRF predicts almost zero wind speeds while MRAMS does not. This may be due to the slightly higher resolution of the MarsWRF simulation, which allows the nighttime downslope flows on Jezero’s western rim to be modeled in more detail, and may make the difference between those flows reaching the landing site or not. In addition, MarsWRF predicts generally more southerly afternoon wind directions than MRAMS outside of summer – although MRAMS occasionally predicts more southerly wind directions too. In general, however, the predicted diurnal and seasonal variation in wind speed and (in particular) wind direction is very similar at the landing site location. Moreover, the range of predicted wind magnitudes is comparable in mesoscale MarsWRF and MRAMS, although MarsWRF predicts stronger winds than MRAMS at $\text{Ls}=90^{\circ}$, especially earlier in the afternoon.

The similarity in the timing and magnitude of predicted wind speeds between the simulations extends to the Midway and NE Syrtis locations, with mesoscale MarsWRF winds again predicted to be stronger than those in MRAMS at $\text{Ls}=90^{\circ}$. Interestingly, however, once outside the crater the pre-dawn wind speeds are far more similar between the two simulations. This may be due to the slight resolution difference between the two models not being relevant when it comes to capturing flows outside the crater itself.

Comparing the three locations, wind speeds during the daytime (when peak speeds occur in both simulations in all seasons shown) are predicted to either remain similar or increase modestly from the landing site to Midway to NE Syrtis. While upslope winds in NW Jezero crater and NW Isidis basin are in roughly the same direction and hence might be expected to interfere constructively, there is a slight non-alignment of the upslope direction at the landing site and across this portion of the Isidis Basin slope. Hence the crater topography actually appears to slightly weaken the flow at the landing site, compared to that outside it (see 4 pm plots in Fig. 4). Interestingly, overnight peak wind speeds around midnight and in the early morning hours are suppressed at the Midway location compared to the other two locations in all seasons shown here. This may be due to the landing site receiving a “bump” (at least occasionally, in MarsWRF) in downslope winds from the nearby crater slope, and NE Syrtis (outside the crater) receiving a similar “bump” due to a ridge to its west, while Midway (also outside but closer to the crater) does not. In reality, smaller-scale topographic obstacles – e.g. small canyons or buttes – will be capable of producing much larger variations in wind speed and direction along the rover traverse, as observed by e.g. MSL in Gale crater (Newman et al. 2017).

Wind directions show slightly more changes with location, with both simulations predicting a marginally greater northerly component to nighttime winds at Midway, and more clearly at NE Syrtis, most likely due to local topography.

Determining where multiple model predictions agree or disagree with observations – and determining the causes of those differences by comparing the physics and setup of those models – is vital to make progress in better understanding and predicting the near-surface circulation. While seasonal changes are important to understanding, the relationship between winds and topography is also of key interest, especially on Mars with its major topography. Hence there is great potential for refining our understanding of the model physics associated with predicting winds if Mars 2020 measures even small changes with location over the course of the mission.

## Multi-model Predictions of Sand Fluxes and Bedform Characteristics in the Jezero Region

Section 8.1 predicts sand fluxes at the landing site as a function of season for eight simulations, while Sect. 8.2 combines a full year of sand flux predictions to find the expected orientation and migration direction of bedforms at the landing site. Section 8.3 uses results from the mesoscale MarsWRF simulation to examine the expected variation in sand fluxes with location over the region that may be traversed by the rover, while Sect. 8.4 uses the same results to examine the predicted bedform orientations and migration directions across the whole Jezero region. These predictions will be useful for anticipating the timing and location of peak aeolian activity during the mission, for comparing with the seasonal and diurnal timing of observed grain motion and surface changes made by Mars 2020 cameras, and for placing observations along the rover traverse in context with nearby aeolian observations made from orbit.

### Predicted Seasonal Variation of Sand Flux and Net Transport Direction at the Landing Site

**Fig. 11:**
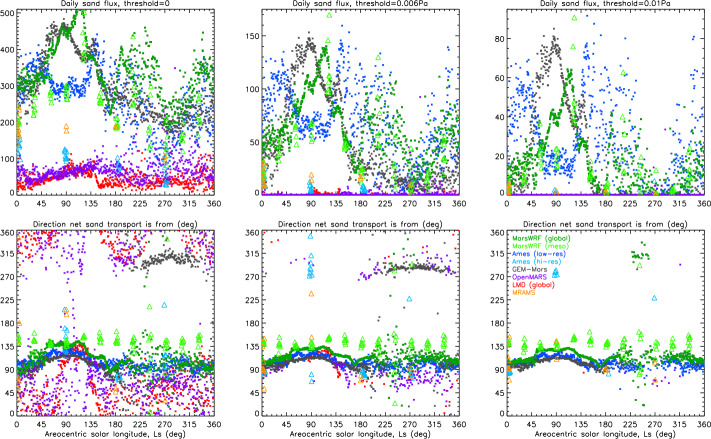
Predicted seasonal variation of daily total sand flux (top row) and net sand transport direction (bottom row) for eight simulations, assuming the Lettau and Lettau (1978) sand flux formulation and a threshold wind stress for saltation of 0 Pa (left column), 0.006 Pa (middle column), and 0.01 Pa (right column)

Figure 11 shows the daily total sand flux and net sand transport direction assuming three different wind stress thresholds, predicted at the landing site as a function of season using the methods described in Sect. 4.1. Results are shown for eight out of nine simulations, as output was only available at the landing season for the mesoscale LMD simulation. The seasonal variation of daily total sand flux for a threshold of 0 (Fig. 11a) has a very similar pattern to the seasonal of daily mean wind stress shown in Fig. 5f. While wind stress depends on $u_{*}^{2}$, sand flux calculated using the Lettau and Lettau (1978) formula depends on $u_{*}^{3}$, which accentuates further the differences in wind magnitudes between the simulations. This results in the global LMD and OpenMARS simulations predicting sand fluxes during summer that are ∼ three times lower than in the mesoscale MarsWRF and Ames global simulations, and ∼ five times lower than in the global MarsWRF and GEM-Mars simulations.

As the threshold increases, the difference between simulations increases further, as some begin to have the majority of wind stresses below the assumed threshold. For a threshold of 0.006 Pa, the global LMD simulation predicts that saltation will occur mostly in the first half of summer. For a threshold of 0.01 Pa, virtually no saltation at all is predicted by the global LMD or OpenMARS simulations, while most other simulations predict saltation will cease for at least part of the $\text{Ls}\sim 225\text{--}315^{\circ}$ period – i.e., around winter solstice.

Ignoring the two simulations for which we only have information at four times of year (MRAMS and high-resolution Ames), most simulations predict peak sand fluxes in roughly the first half of summer, $\text{Ls}\sim 85$ to $150^{\circ}$, for all thresholds shown, although the low-resolution Ames and both MarsWRF simulations also predict large fluxes in mid-fall ($\text{Ls}\sim 210^{\circ}$), with the low-resolution Ames simulation also predicting large fluxes from late winter through early spring ($\text{Ls}\sim 340\text{--}45^{\circ}$). In addition, the global LMD simulation predicts its strongest sand fluxes in a handful of sols during winter, and the OpenMARS simulation also predicts peak sand fluxes during fall and winter, though with great variability from sol to sol.

The daily net sand transport direction has a clear physical meaning: it is a vector sum of the sand transport over every time of sol, giving the expected direction in which a sand grain would be observed to move over that sol, or in which a sand pile might be expected to elongate, or a ripple field migrate (see e.g. Wilson 1971 and Sect. 4.2). Larger aeolian features would be expected to evolve more in line with the time-integrated effects of such sand fluxes over a full Mars year (or more), as discussed in Sect. 4.3 and examined in Sect. 8.2.

In all models, the predicted net sand transport direction is distinctly different around summer solstice than at other times of year. For a threshold of 0 Pa, and away from summer solstice, most models predict a spread in net sand transport directions, although transport from a direction of $\sim150\text{--}270^{\circ}$ (roughly, transport toward the NNW and clockwise through Eastward) is very rare. Around summer solstice, however, most simulations show a much narrower spread in predicted net transport direction, with most models predicting transport from a direction of $\sim115\text{--}135^{\circ}$ (i.e., transport toward the WNW through NW) in all sols. The major outliers are the OpenMARS simulation, which predicts net transport from nearly every direction right around solstice, and the high-resolution Ames and MRAMS simulations, which predict a greater spread of directions up to $\sim205^{\circ}$ (i.e., transport toward the NNE). Excluding those outliers, the time of peak sand flux in each simulation coincides with a net sand transport direction in the range $110\text{--}155^{\circ}$.

As noted above, however, a threshold of 0 Pa is unphysical, thus of more interest is the predicted net sand transport direction for more realistic values of the threshold. Interestingly, as the threshold is increased the simulations converge further in terms of sand flux direction. For a threshold of 0.006 Pa, the range of predicted net sand transport directions shrinks to $\sim55\text{--}155^{\circ}$ for most simulations, the exceptions being in the second half of the Mars year (when transport is also from $\sim280^{\circ}$ in some sols for several simulations) and for MRAMS and the high-resolution Ames simulation around summer solstice. And for a threshold of 0.01 Pa, virtually all sand transport is from directions between $\sim90$ and $160^{\circ}$, or from between $\sim90$ and $135^{\circ}$ (i.e., toward the W through NW) if only low-resolution models are included.

In summary, roughly the first half of summer ($\text{Ls}\sim85\text{--}150^{\circ}$) is predicted to have the strongest aeolian activity in terms of wind-driven sand fluxes in most simulations, with net sand transport over this period predicted to come from between $\sim110$ and $135^{\circ}$ (i.e., toward the WNW through NW) for a threshold of 0.006 Pa or higher. The higher-resolution mesoscale MarsWRF simulation predicts sand transport at the landing site more toward the NNW. By contrast, the weakest aeolian activity in terms of wind-driven sand fluxes is predicted to occur around local winter solstice ($\text{Ls}=270^{\circ}$) in most simulations, despite this being the time of the strongest mean circulation often referred to as the “windy season” on Mars, due to regional topographic winds associated with the NW rim of Isidis basin that oppose the mean circulation at this time of year. The main exception is the OpenMARS simulation, which predicts peak sand fluxes in fall and winter, with transport to the WSW or ESE.

For the Mars 2020 mission, the majority of results suggest that most aeolian activity in terms of saltation should occur between $\text{Ls}\sim 340$ and $225^{\circ}$ – i.e., in late winter through mid fall – with reduced activity in the second half of fall and most of winter. This of course assumes that no major dust storms occur. It would be very interesting to see how the models predict the circulation and aeolian activity might change if an equinoctial or solsticial global dust storm occurred. This is beyond the scope of the present study but will hopefully be investigated before Mars 2020 experiences its first dust storm season on Mars.

### Predicted Net Annual Transport at the Landing Site

Table 3 shows the resulting total annual sand fluxes and net sand transport directions at the landing site for ten thresholds, as predicted by each simulation shown in Fig. 11. The MRAMS and high-resolution Ames predictions vary greatly with threshold, likely due to having access to simulation output at only four times of year as well as large sol-to-sol variability that may not be sufficiently captured by the small number of sols used in each period (especially for MRAMS, for which only two sols per season are used), so are not considered further. As suggested by the Fig. 11 results, the net sand transport direction over a full year converges to between 103 and $119^{\circ}$ (transport toward the WNW) at a threshold of 0.01 Pa in four of the remaining six simulations (GEM-Mars, global LMD, low-resolution Ames and global MarsWRF). By contrast, OpenMARS predicts a net sand transport direction of $71^{\circ}$ (transport toward the WSW), which reflects its peak wind speeds occurring in local winter when daytime wind directions are most northerly (see Fig. 8), while mesoscale MarsWRF predicts a direction of $143^{\circ}$ (transport toward the NW), due to its more southerly daytime wind directions than other simulations except around summer solstice (see Fig. 11).

**Table 3:** Predicted total annual sand flux and net sand transport direction (given as the direction from which the sand is transported, in deg clockwise relative to N) for ten different wind stress thresholds for the seven simulations shown in Fig. 11. *The mesoscale MarsWRF prediction is made using 7 sols of output every $30^{\circ}$ of Ls, scaled up by the number of sols corresponding to that time of year (e.g. there are more sols per $30^{\circ}$ of Ls around aphelion than perihelion) then summed to give an annual total. **The Ames high-resolution prediction is made using 10 sols of output every $90^{\circ}$ of Ls, multiplied by $669/(10*4)$, and the MRAMS prediction is made using only 2 sols of output every $90^{\circ}$ of Ls, multiplied by $669/(2*4)$, hence both provide only a rough estimate and are likely not representative of the behavior predicted if the year were sampled more completely

Simulation		Threshold wind stress (Pa)									
		0	0.002	0.004	0.006	0.008	0.01	0.012	0.014	0.016	0.018
GEM-Mars	Flux	2.04e5	1.01e5	6.19e4	3.69e4	2.19e4	1.33e4	7.54e3	3.57e3	1.11e3	108
	**Dir**	**102**	**105**	**106**	**106**	**107**	**108**	**108**	**109**	**109**	**109**
LMD global	Flux	3.06e4	4.98e3	855	139	14.3	0.479	0	0	0	0
	**Dir**	**59**	**79**	**92**	**97**	**98**	**107**	**–**	**–**	**–**	**–**
OpenMARS	Flux	4.63e4	1.06e4	2.82e3	771	218	71.3	23.5	7.18	1.12	0
	**Dir**	**48**	**61**	**68**	**71**	**71**	**71**	**72**	**72**	**74**	**–**
Ames low-resolution	Flux	1.80e5	9.76e4	6.78e4	4.67e4	3.07e4	1.83e4	9.15e3	3.74e3	1.03e3	126
	**Dir**	**104**	**105**	**105**	**104**	**104**	**103**	**101**	**100**	**99**	**99**
MarsWRF global	Flux	2.32e5	1.18e5	7.374	4.48e4	2.55e4	1.26e4	4.72e3	1.08e3	80.2	0
	**Dir**	**114**	**116**	**118**	**118**	**119**	**119**	**122**	**125**	**126**	**–**
Ames high-resolution**	Flux	6.04e4	2.27e4	1.09e4	4.77e3	1.72e3	445	94.4	37.0	13.6	0
	**Dir**	**90**	**90**	**86**	**86**	**90**	**140**	**234**	**229**	**230**	**–**
MarsWRF mesoscale*	Flux	1.62e5	8.03e4	5.01e4	3.07e4	1.82e4	9.98e3	4.86e3	2.11e3	832	312
	**Dir**	**146**	**144**	**143**	**143**	**143**	**143**	**144**	**146**	**148**	**152**
MRAMS**	Flux	1.10e5	4.52e4	2.28e4	9.84e3	3.49e3	1.14e3	304	84.6	7.64	0
	**Dir**	**356**	**355**	**354**	**348**	**343**	**35**	**322**	**270**	**263**	**–**

### Predicted Seasonal Variation of Sand Fluxes at Jezero, Midway, and NE Syrtis

**Fig. 12:**
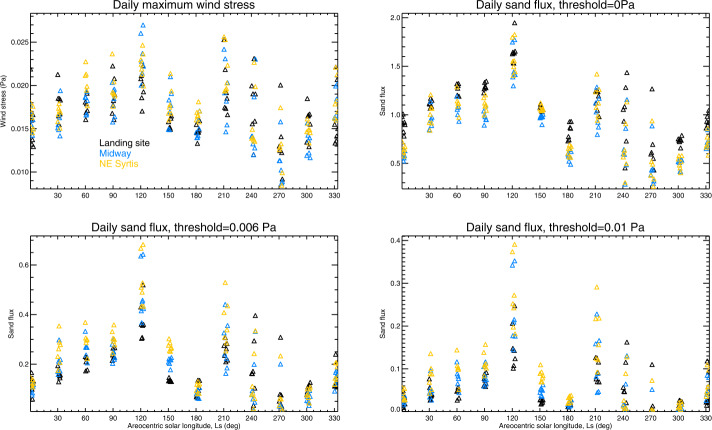
Mesoscale MarsWRF domain 5 predicted daily maximum wind stress (top left), as well as the daily sand flux for three threshold wind stress values, for the landing site, Midway, and NE Syrtis locations, as a function of time through the Mars year (Ls). Note that the sand fluxes are in arbitrary units; given the uncertainties for Mars, we do not apply a constant of proportionality in front of the RHS of Eq. (6)

Over the course of the Mars 2020 mission the Perseverance rover may drive from the landing site to the Midway and perhaps also the NE Syrtis locations (shown in Fig. 1b). Figure 12 compares the predicted seasonal variation of wind stress and sand fluxes at all three locations, using output from the mesoscale MarsWRF simulation only. In line with the variation in wind speeds across the three locations (Sect. 7 and Fig. 9), there is a trend toward generally larger sand fluxes at Midway than at the landing site, and at NE Syrtis than at Midway, but this becomes most apparent and significant as the threshold increases. During the mission, these mesoscale MarsWRF results suggest that most aeolian activity in terms of saltation will likely occur in summer or in mid-fall, especially in later years of the mission as the rover traverses out of Jezero toward Midway and perhaps NE Syrtis.

### Predicted Bedform Orientations and Migration Directions Across the Jezero Region

**Fig. 13:**
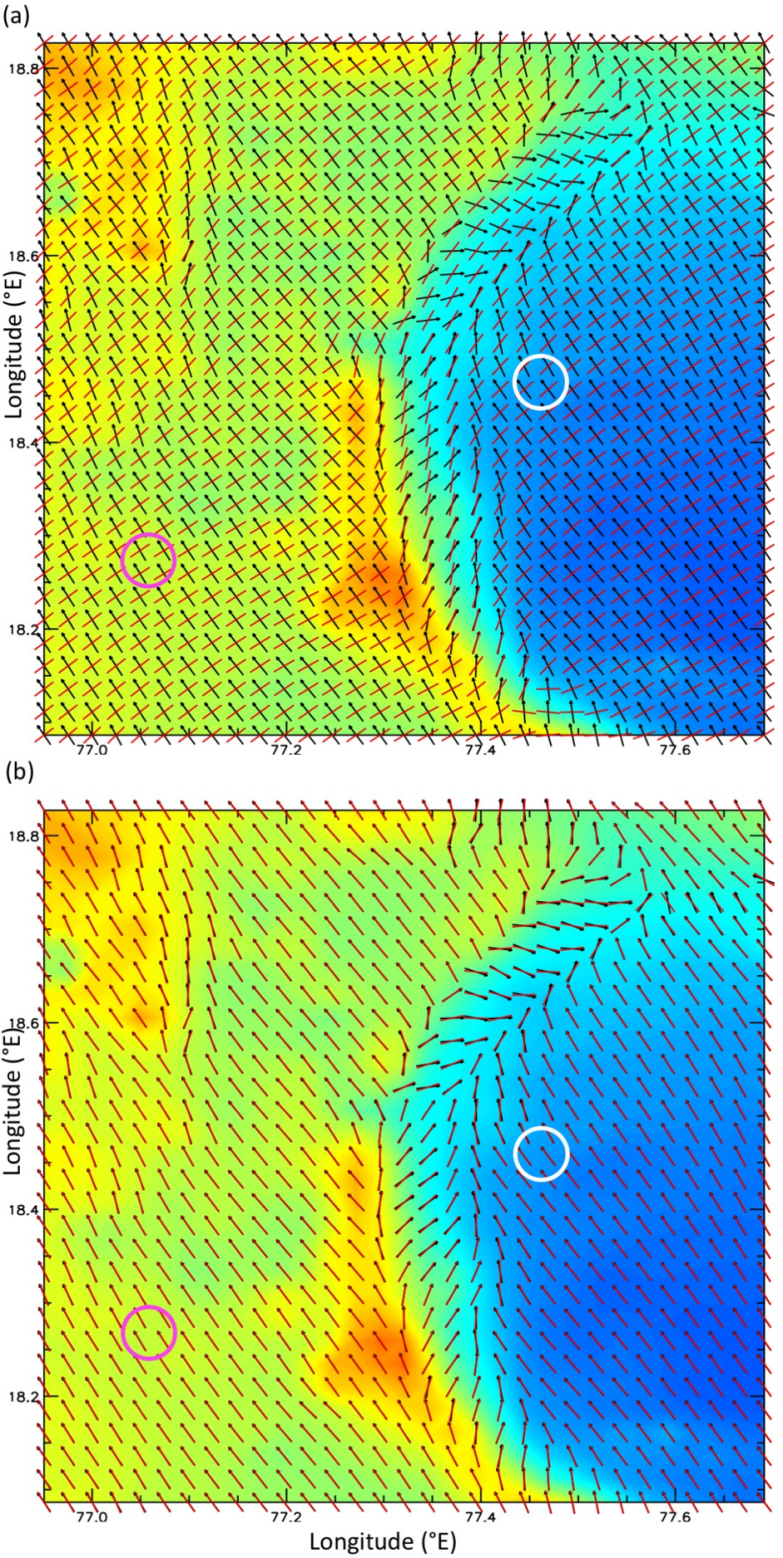
Predicted net dune migration direction over a Mars year (black arrows) as well as predicted orientation of bedform crests (red lines) assuming the (**a**) GBNT or (**b**) Fingering Mode (bottom) theory of dune formation, for the region surrounding the landing site in NW Jezero crater using mesoscale MarsWRF output. The shaded background shows topography. Note that the net dune migration is independent of the chosen dune formation theory hence the black arrows are identical between plots. Also shown is topography (colored shading) and the location of the Mars 2020 landing site and Midway (white and pink circles, respectively). Note that arrows show only net sand transport direction and are not scaled to show predicted sand flux

Following the steps laid out in Sect. 4.3, we use the annual cycle of sand fluxes from the mesoscale MarsWRF model, sampled every $30^{\circ}$ of Ls, to produce Fig. 13, which shows bedform orientations (red lines) and migration directions (black arrows, which are not scaled by sand flux) for a threshold of 0.005 Pa, across the region covering the landing site and Midway locations. As MRAMS predictions were only available at four times of year we did not repeat this exercise using those output. Figure 13 shows that the dominant transport toward the NW, predicted at the landing site by mesoscale MarsWRF, is present across most of Jezero crater and much of the region outside the crater also. Different sand transport directions are predicted near the crater rim, however, due to the perturbation of winds there by the strong topographic gradients (as shown in the animation of mesoscale MarsWRF winds at $\text{Ls}\sim 5^{\circ}$ in Online Resource 3). In fact, net sand transport toward the N or NE is predicted in several areas.

In terms of predicting bedform orientations, the GBNT theory (Fig. 13a) predicts these will largely be from SW to NE over most of the area shown. Again, however, near the crater rim a greater range of orientations is predicted, including nearly S to N (just inside the western part of the crater) and W to E (on the area of high topography outside it). Interestingly, some of the bedforms along or just inside the western crater rim are predicted to be nearly aligned with the net transport direction, suggesting nearly equal and opposite sand fluxes from either side of the bedform over a year. By contrast, the Fingering Mode theory (Fig. 13b), which should apply in the case of a limited sand supply, predicts dunes virtually parallel to the predicted sand transport directions in all locations. Under this assumption, bedforms would be oriented from NW to SE, again except along the crater rim where bedforms oriented SW to NE or S to N would occur, and to the east of a topographic ridge (top left) where S to N bedforms are also predicted.

## Predicted Dust Devil Activity in the Jezero Region and at Other Landing Sites

Following the theory laid out in Sect. 4.5, in Sect. 9.1 we use seven sols of output from the mesoscale MarsWRF simulations every $30^{\circ}$ of Ls to predict the seasonal and diurnal cycles of sensible heat flux, vertical thermodynamic efficiency, and dust devil (or convective vortex) activity (DDA) across the Jezero region. (Note that the variables needed to calculate DDA were not available from the other simulations at the time of writing.) As discussed in Sect. 8, these predictions may be useful for anticipating the timing and location of peak aeolian activity during the mission. They may also be useful for comparing with the observed seasonal, diurnal, and spatial variation of meteorological indications of vortex activity (e.g. rapid pressure drops), dust devil imaging, and surface changes or rover “cleaning events” that may be associated with raising of dust by dust devils. In Sect. 9.2 we compare with predictions for two other missions currently operating on Mars’s surface, MSL and InSight, to place the Mars 2020 predictions in context.

### Predicted Variation of Dust Devil Activity Across the Jezero Region

**Fig. 14:**
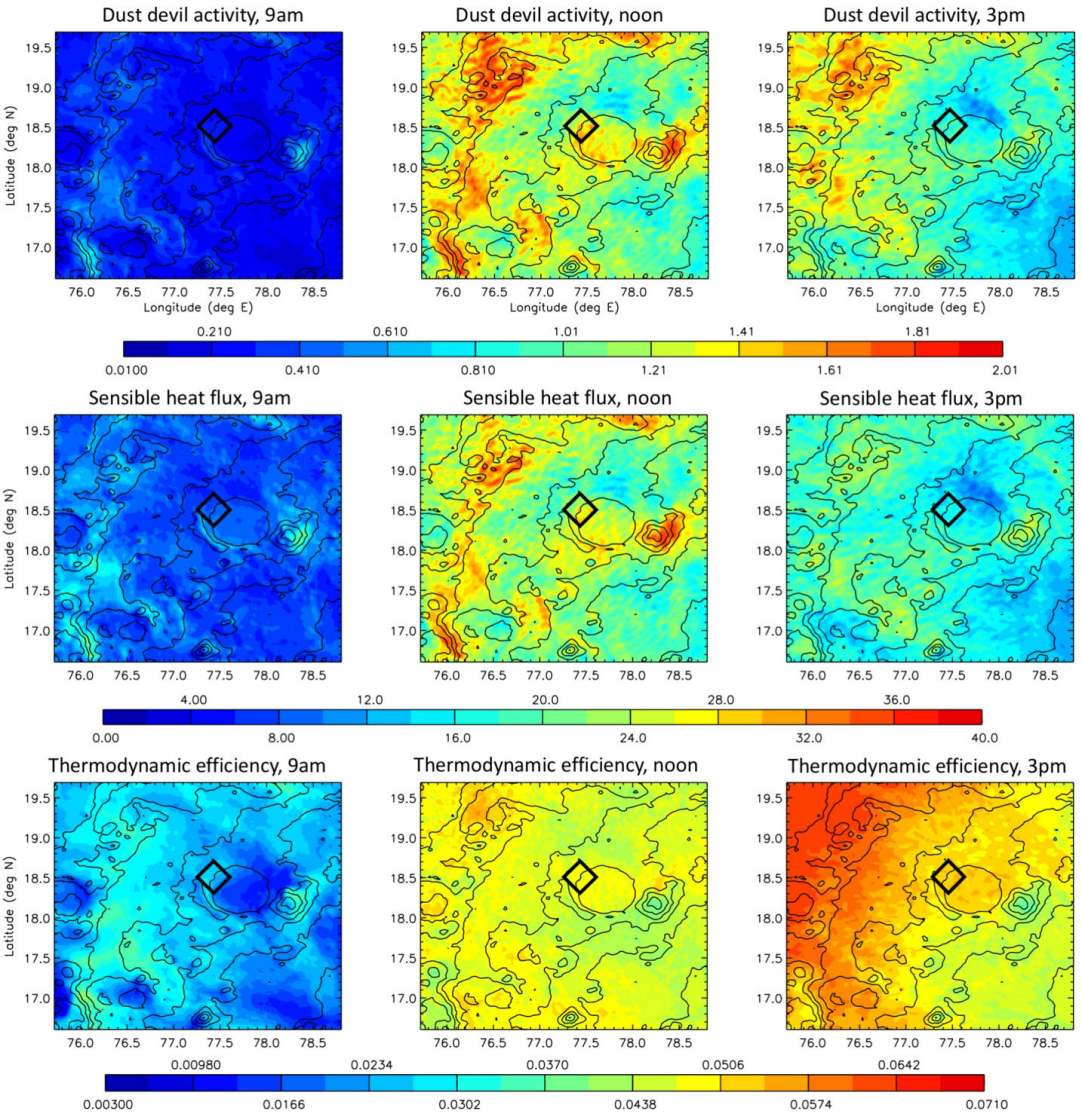
Predicted DDA (top row), sensible heat flux (middle row), and vertical thermodynamic efficiency (bottom row) across MarsWRF domain 5 at local summer solstice ($\text{Ls}=90^{\circ}$), for 9 am (left column), noon (middle column), and 3 pm (right column) LTST. The unit of sensible heat flux is $\text{Wm}^{-2}$. The black diamond is centered on the landing site coordinates and is sized to guide the eye without obscuring shading

Figure 14 shows the predicted spatial variation of DDA, sensible heat flux ($F_{S}$), and vertical thermodynamic efficiency ($\eta $) across domain 5 of the MarsWRF simulation at three times of sol for one time of year (local summer solstice, $\text{Ls}=90^{\circ}$). The landing site, Midway, and NE Syrtis are all located inside the black diamond. Several areas stand out as having far lower predicted DDA around noon and at 3 pm, including a narrow region to the north of Jezero crater and a wider region to the south east. The region to the west of Jezero, corresponding to the rim of the Isidis basin, also shows up as having larger DDA values. Looking back at Fig. 4 (second row), these appear to correspond to changes in the wind pattern at this time of year, such as the increased strength of daytime upslope winds on the basin rim. A similar (albeit smaller) enhancement in the DDA and sensible heat flux is visible at noon on the western slopes of Jezero crater, again due at least in part to the slight increase in winds there shown in Fig. 4 (second row).

**Fig. 15:**
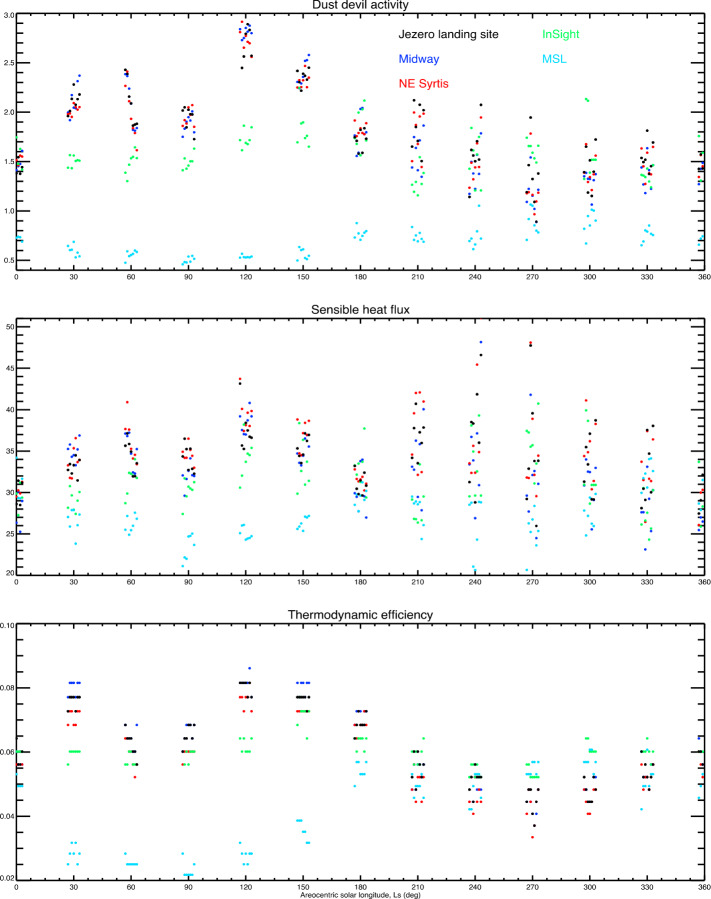
Seasonal variation of daily maximum (**a**) dust devil activity (DDA), (**b**) sensible heat flux, and (**c**) vertical thermodynamic efficiency, predicted using 7 sols of output every $30^{\circ}$ of Ls from domain 5 of the mesoscale MarsWRF simulation, for the Mars 2020 landing site, Midway, and NE Syrtis locations. A similar model setup and resolution, centered on the InSight landing site, was used to make the InSight predictions (e.g. Baker et al. 2020). A similar model setup and resolution, except using vertical grid B described in Newman et al. (2017) and centered on the middle of Gale crater, was used to make the MSL predictions, which are for the location of MSL at the start of its fourth year on Mars (on MSL Sol 2007 when the rover was partway up Aeolis Mons at $-4.725, 137.381\text{E}$). The unit of sensible heat flux is $\text{Wm}^{-2}$

Predicted variations along the potential rover traverse may be demonstrated more quantitatively by extracting time-series at different locations. Figure 15 shows the predicted seasonal variation of the daily maximum DDA, $F_{S}$, and $\eta $ at the landing site (in black), Midway (in royal blue), and NE Syrtis (in red). Similar predictions at the locations of MSL and InSight are also shown, and are discussed in Sect. 9.2. The first point to notice is that there is no consistent difference between predictions for the three locations. This is likely due to the relatively weak variation of surface properties, including topography, over this region. As discussed in Sect. 7, while small differences are expected between wind speeds at the three locations, affecting $u_{*}$, they are evidently not large enough to produce consistent variations in $F_{S}$ or the DDA with respect to location. By contrast, there is significant variation of $F_{S}$ with season (Fig. 15b), caused by stronger wind speeds in summer (see Fig. 5e and g) and stronger surface-to-air temperature gradients in some sols (see Fig. 5c and d) in winter.

Unlike $F_{S}$, $\eta $ (Fig. 15c) – which is related to boundary layer depth – is at its lowest in the winter season, instead peaking in mid-to-late summer with values that are at least double. Note that the daily maximum in $F_{S}$ will occur earlier in a given sol than the maximum in $\eta $, due to the time needed between peak heating and maximum boundary layer extent; this can be seen by comparing the noon and 3 pm plots in Fig. 14. Hence the daily maxima in $F_{S}$ and $\eta $ will differ from their values at the time of peak DDA. However, the overall result (Fig. 15a) is a prediction of peak daily maximum DDA in mid-to-late summer with the lowest DDA predicted around winter solstice.

### Comparison with Other Missions

Based on MSL observations, Newman et al. (2019a) suggest that DDA above a certain threshold value may be proportional to the number of vortex pressure drops above a certain intensity. However, it remains unclear how one should convert the DDA into a predicted number (or intensity) of vortex pressure drops per hour, say. In addition, the number of dust devils imaged may depend greatly on surface dust availability. We thus seek context for the meaning of these DDA predictions by comparing with predictions for two ongoing missions, at which the vortex activity has already been observed. Hence Fig. 15 also shows MarsWRF predictions for the InSight landing site (in green) and for the location of MSL (in light blue) at the start of its fourth year on Mars (on MSL Sol 2007 when the rover was partway up Aeolis Mons at $-4.725, 137.381\text{E}$). This location is chosen for MSL because the number of vortex pressure drops and predicted dust devil activity were both found to increase (when comparing the time of year) as the rover climbed over its first three years (Newman et al. 2019a). A similar model setup was used to produce these simulations but with domain 5 now centered on respectively the InSight landing site and center of Gale crater; see details in Newman et al. (2019b) and Newman et al. (2017, 2019a), respectively.

If we first examine the seasonal trends in predicted DDA for all three missions, and account for the fact MSL sits in the southern hemisphere (hence local summer solstice occurs at $\text{Ls}\sim 270^{\circ}$), we find generally similar behavior patterns of predicted daily maximum vertical thermodynamic efficiency and DDA with respect to local summer and winter across all three missions. The relationship to wind patterns (which are not shown for InSight or MSL in this paper) makes predictions of sensible heat flux harder to interpret, but they appear to have a smaller seasonal range than PBL thickness hence have a smaller impact on seasonal DDA variation. In all cases, thermodynamic efficiency (related to PBL thickness) is lower in local fall and winter than in spring and summer, with a minimum around winter solstice and a maximum in mid-to-late summer. Thus the predicted DDA also has a similar seasonal variation at all sites, although the increase in local summer appears to begin slightly earlier at MSL than at InSight or Jezero.

However, the most obvious differences between the three missions is in the magnitude of maximum daily DDA predicted. The largest values predicted at MSL barely exceed the smallest values predicted at Jezero and are entirely below the smallest values predicted at InSight. In addition, while InSight’s lowest maximum daily DDA values exceed the lowest predicted for Jezero, the maximum daily DDA expected at InSight is well below the maximum expected in Jezero. In short, these predictions suggest that peak vortex activity in Jezero will be greater than that at InSight, and significantly greater than at MSL’s location in Gale crater.

These predictions are consistent with greater numbers of and larger magnitudes of vortex pressure drops having been measured by InSight than by MSL (e.g. Newman et al. 2019a; Spiga et al. 2020). This suggests that a larger number of and/or larger magnitude of vortex pressure drops should be measured by the MEDA pressure sensor on Mars 2020. This might also suggest that Mars 2020 cameras will capture many images of dust devils. However, while InSight’s location in Elysium Planitia has many dust devil tracks visible on the surface (Perrin et al. 2020) and while InSight’s cameras have detected several surface changes attributed to passage of a vortex (Baker et al. 2020; Charalambous et al. 2021), no dust devils have yet been imaged by InSight’s cameras in more than a Mars year of operation. Conversely, while no clear dust devil tracks have been seen inside Gale crater, MSL has imaged well over a hundred dust devils in its more than four Mars years of operation (personal communication, Mark Lemmon). One possible explanation is a lack of available dust at InSight’s location. If only a single layer of dust were available, vortices might raise it easily from the surface and in doing so would bare the underlying regolith (changing the albedo and causing tracks to appear on the surface), but would run out of supply before the vortex becomes dusty enough to be visible. Thus we only predict that Jezero will have more and larger vortex pressure drops than measured by MSL or InSight, rather than predicting large numbers of imaged dust devils.

**Fig. 16:**
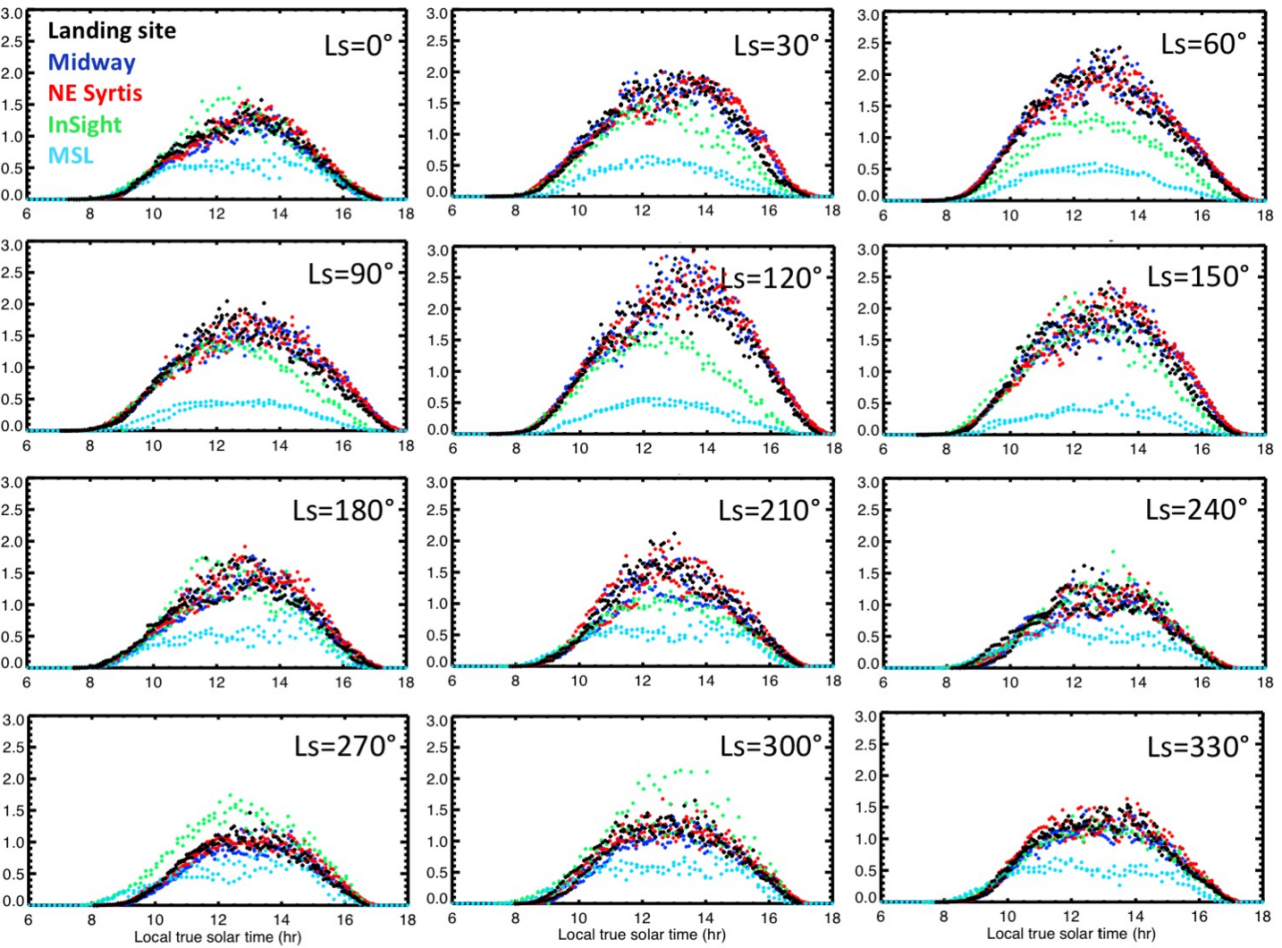
As in Fig. 15 but now showing the diurnal variation of DDA for 12 times of year at all five locations

Figure 16 shows the predicted diurnal variation of DDA at the Mars 2020 landing site, Midway, and NE Syrtis, compared with the two other missions. Peak DDA is predicted between $\sim12{:}00$ and 14:00 throughout the year, and slightly later (by up to an hour) than the time of peak DDA predicted at InSight in some seasons. For $\text{Ls}\sim 60\text{--}120^{\circ}$, the DDA remains non-zero up to 20 minutes later in the day than at MSL or InSight.

We end by noting that, as discussed in Sect. 4.5, these predictions do not include the effects of IR radiative heating or the observer effect that results in more vortex detections for faster ambient wind speeds. Both effects should be considered before comparing the predicted DDA with MEDA-derived vortex pressure drop statistics in Jezero crater.

## Comparing Predicted Aeolian Activity and Features with Orbital Observations

In this section we compare the predictions of Sects. 8 and 9 with evidence of aeolian activity observed from orbit.

### Wind Streaks

Day and Dorn (2019) measure several aeolian features inside Jezero crater itself. Wind streaks on the crater floor, 334 of them in total, were oriented consistently east-to-west (toward a direction of $263^{\circ}\pm8^{\circ}$) over the $\sim10$-year coverage of HiRISE imaging, with no apparent variation with season, although the majority of wind streak measurements were made around $\text{Ls}\sim 180^{\circ}$ or in local winter ($\text{Ls}\sim 270\text{--}340^{\circ}$). This is roughly consistent with easterly (or slightly more northerly) wind directions at the LTST of peak wind stress predicted by most of the simulations at $\text{Ls}\sim 180^{\circ}$ and $270^{\circ}$, especially the high-resolution Ames, global LMD, OpenMARS, and MRAMS simulations (Figs. 7f and 8f). Least consistent are results from the mesoscale MarsWRF simulation, which predicts ∼south-easterly winds at those times. However, a complication is the fact that 80% of the observed streaks were bright streaks (Taylor Dorn, personal communication, 2020), which are typically associated with deposition in the lee of obstacles, rather than dark streaks which are more likely to be erosional. As discussed in Fenton and Richardson (2001), depositional streaks are likely to occur at times when the atmosphere is more stable, which may not coincide with the strongest wind speeds. Hence more detailed investigation is needed to assess the daytime stability conditions in the models and the implications for when the streaks may have formed.

### Bedform Orientations and Transport Directions

Chojnacki et al. (2018) use HiRISE images to identify numerous dunes to the north, west, and east of the landing site, and estimate sand fluxes for those that are active. High sand fluxes are found in dune fields in a crater to the NE of Jezero crater, with low flux dunes in Jezero crater’s inlet channel, and a patch of smaller sand ripples adjacent to the fan are active also. The dunes closest to Jezero show migration toward the WNW, while sand ripples closer to the landing site also show migration but with an unclear direction. In addition, Day and Dorn (2019) map seven barchanoid dunes with distinct lee faces in the inlet channel valley crossing Jezero crater’s northern rim. These dunes are elongated parallel to channel walls, and the dip direction of the dune lee faces ranges from northwest to southwest, indicating a dominance of transport and migration to the west overall. Superimposed large ripples largely reflect the orientations of the dune lee faces, with more complex patterning near the dune crests. These findings appear consistent with the majority of simulations predicting net sand transport toward the WNW or slightly N of W for reasonable threshold wind stresses (Sect. 8.2, Table 3) but less consistent with predictions of transport to the WSW (OpenMARS) and inconsistent with simulations that predict net transport toward the NW over much of the Jezero region (mesoscale MarsWRF; see also Fig. 13).

### Yardangs

Day and Dorn (2019) find that yardang erosional features are oriented SW-NE, with blunting on the southwest side suggesting formative winds from the southwest rather than the northeast. Given the difference in implied wind direction compared to other aeolian features, Day and Dorn (2019) suggest that these yardangs are evidence of an ancient wind regime. This idea is supported by our predictions that the strongest present-day sand transport is primarily from the ESE (Fig. 11), and by the fact that no model predicts significantly south-westerly winds or sand transport in any of the seasons shown here (Figs. 2, 6–8, and 11).

### Dust Devils and Tracks

A full survey of dust devils and/or dust devil tracks has not been published for Jezero crater. However, an image taken at $\text{Ls}\sim 270^{\circ}$ by the CaSSIS camera on the Trace Gas Orbiter shows a large number of dust devil tracks, including in the vicinity of the landing site (Nick Thomas, CaSSIS Principal Investigator, personal communication). Such images may be used both to infer daytime wind directions at the time of track formation and – if taken over a range of Ls – to infer seasonal variations in dust devil activity. This will be investigated both prior to and during the course of the Mars 2020 mission, and will hopefully enable correlations between orbital observations of dust devils/tracks and surface observations of dust devils and evidence of vortices, as has been possible most recently for InSight (Perrin et al. 2020).

## Summary and Conclusions

Nine simulations using seven Mars atmospheric models are used to predict the meteorology and aeolian activity of the Mars 2020 landing site in Jezero crater, Mars. The models use a wide range of different dynamical solvers, parameterizations of sub-gridscale processes, and atmospheric dust distributions, although all dust distributions are intended to represent a year without a major dust storm. The horizontal grid spacings also vary between models, ranging from $2^{\circ}$ to $5^{\circ}$ across the five low-resolution simulations and from $\sim1.4$ to $\sim10~\text{km}$ across the four high-resolution simulations. In combination, the results shown in this paper provide a wide envelope of predictions for the Mars 2020 mission. They also provide model-to-model comparisons that may be used to identify model errors and make improvements, especially as ground truth data for Jezero crater become available during the mission.

There is generally strong agreement on the predicted seasonal variations of pressure across all simulations. This is largely due to the parameters that influence the CO_2_ cycle having been tuned in most models to match the pressure curves at the Viking Landing site. Minimum surface pressure is predicted at $\text{Ls}\sim 145^{\circ}$ and maximum at $\text{Ls}\sim 250^{\circ}$, with all simulations agreeing to within $10^{\circ}$ of Ls on the timing of the maxima and minima. Diurnal cycles of surface pressure at the landing season ($\text{Ls}\sim 5^{\circ}$, shortly after spring equinox) and other cardinal seasons ($\text{Ls}\sim 90$, 180, and $270^{\circ}$) also show strong similarities across simulations, although differences include the number of daily pressure maxima (especially for the landing season, $\text{Ls}\sim 5^{\circ}$), the relative strength of peaks, the semidiurnal amplitude, and the diurnal pressure range. Overall, however, we find that the equinoxes have three or four maxima each sol while the solstices have two; the dusty season ($\text{Ls}\sim 270^{\circ}$) generally has the strongest semi-diurnal tidal amplitudes; and the diurnal pressure range is generally smallest at $\text{Ls}\sim 90^{\circ}$.

Similar agreement is found in predicted surface and atmospheric temperatures, with at most $\sim10~\text{K}$ differences between simulations in terms of their predicted maximum, mean, and minimum temperatures at any time of year. Differences in predicted nighttime temperatures cannot be explained by the different thermal inertias assumed at the location of the landing site, thus more investigation is needed. However, differences in daytime air temperatures are almost certainly due to these being output at different heights in the surface layer in some simulations, thus a future intercomparison study should be sure to extrapolate them to the same altitude. Despite these differences, all simulations predict maximum daily max and mean surface and air temperatures at $\text{Ls}\sim 180^{\circ}$, with a secondary maximum in max temperatures at $\text{Ls}\sim 360^{\circ}$, and minimum daily max and mean surface temperatures at $\text{Ls}\sim270^{\circ}$, with a secondary minimum in max temperatures at $\text{Ls}\sim80^{\circ}$. Temperatures are thus lowest at local winter solstice but are warmest at fall equinox rather than summer solstice. Mars reaches its furthest point from the Sun shortly before northern summer solstice, hence Jezero crater (at $\sim18.5~^{\circ}\text{N}$) does not reach peak temperatures until the following equinox, when peak heating occurs at the equator.

Wind speeds and to some extent directions vary the most between simulations. Jezero crater is located in northern low latitudes, inside and close to the NW rim of the huge Isidis impact basin which sits on the dichotomy boundary, with the Mars 2020 landing site located inside the much smaller Jezero crater and close to its NW rim. At this location, the main circulation components that combine to produce winds are the global circulation, slope winds on the NW slopes of Isidis (which are far more massive than those inside Jezero), and flows associated with thermal tides and their interaction with topography. In general, all simulations predict the winds at the landing site to be dominated by the Isidis basin slope winds in most seasons, with downslope nighttime winds from the NW and upslope daytime winds from the ESE or SE, although the mesoscale MarsWRF simulation predicts a slightly more southerly upslope wind component than other simulations at most times of year. The exception is around local summer solstice ($\text{Ls}\sim90^{\circ}$), when the global circulation creates strong southerly near-surface winds at low latitudes. This reinforces the already-strong daytime upslope flows from the ESE or SE on the NW basin slopes, which results in more southerly daytime wind directions and daytime winds in all simulations. This mechanism is likely why daily mean wind speeds peak in the first half of summer in most simulations, despite the global circulation being generally much stronger in the second half of the Mars year. In two simulations, global LMD and OpenMARS, daily mean wind speeds do peak during the latter period (in mid-fall through late winter), but show significant sol-to-sol variability in wind speed. The timing of daily maximum wind speeds shows more variability between simulations, but peak values generally occur within $\pm45^{\circ}$ of Ls of the daily mean peak, although for the LMD global simulation they occur $\sim90^{\circ}$ of Ls earlier (in mid-summer). Wind speeds also vary hugely between simulations, with the calmest simulation (global LMD) predicting daily maximum speeds between $\sim4$ and $11~\text{ms}^{-1}$ and the windiest (low-resolution Ames) predicting daily maximum speeds between 9 and $19~\text{ms}^{-1}$.

The cause of these differences is likely a complex function of surface properties, boundary layer mixing schemes, dust distribution, and grid spacing (both horizontal and vertical), as well as potentially the method used to extrapolate winds to a height of 1.5 m. A more detailed investigation is needed to understand this.

The two highest-resolutions simulations are used to predict differences in wind speed and direction in the region that may be explored by the Perseverance rover over its mission. Peak wind speeds are predicted to be smallest at the landing site, marginally stronger at Midway overall, and slightly larger again in NE Syrtis in most seasons. While upslope winds in NW Jezero crater and NW Isidis basin overall should be in the same direction and hence might be expected to interfere constructively, a slight non-alignment of the upslope direction at the landing site and across this portion of the Isidis basin slope appears to result in a weakened flow inside the crater versus outside it.

Aeolian activity at the landing site and across the Jezero region is also predicted using the six simulations for which wind stress is available at at least twelve times of year. Roughly the first half of summer ($\text{Ls}=85\text{--}150^{\circ}$) is predicted to have the strongest sustained activity in terms of wind-driven sand fluxes in all but one simulation (OpenMARS). The weakest sand fluxes are predicted around local winter solstice in most simulations, despite this being a time often referred to as the “windy season” on Mars due to the strongest global mean circulation occurring then. In Jezero crater, however, regional topographic winds associated with the NW slopes of Isidis basin oppose the mean circulation, causing peak wind speeds and wind stresses to be consistently smaller in winter for all simulations except OpenMARS, although the global LMD simulation also predicts its largest daily mean wind stresses in a handful of sols in mid-fall and late winter.

Net annual sand transport is predicted to be toward the WNW in four out of the six simulations, due to the strongest winds being daytime east-southeasterlies up the Isidis basin slope. The outlier simulations are mesoscale MarsWRF, which predicts peak sand transport in the first half of summer but net annual transport toward the NW, and OpenMARS, which predicts peak sand transport in the second half of the year and net annual transport toward the WSW. The more northerly sand transport predicted by OpenMARS is due to more notherly wind directions associated with the global overturning circulation in the second half of the year. By contrast, the cause of the more southerly wind directions and hence sand transport predicted by mesoscale MarsWRF vs. all other simulations (including global MarsWRF) is unclear, but appears to be a consequence of feedbacks between very small-scale flows and the regional circulation, rather than a result of better resolving the landing site topography in particular.

Easterly (or slightly more northerly) wind directions are predicted at the LTST of peak wind stress by most simulations at $\text{Ls}\sim 180^{\circ}$ and $270^{\circ}$, which appears consistent with the orientation of wind streaks observed in these periods, although as most of these streaks are bright (hence likely depositional) they may have been emplaced when atmospheric stability was reduced, rather than when wind stress peaked, thus more investigation is needed here. The prediction of net annual sand transport to the WNW at the landing site by most simulations appears consistent with general sand transport directions across the region, as inferred from aeolian features inside Jezero crater and with the observed motion of dunes just to its north. The mesoscale MarsWRF simulation is used to expand this prediction over the region containing the likely Mars 2020 rover traverse, and predicts sand transport to the NW over most regions except close to the crater rim and topographic ridges. This shows that its more southerly daytime wind directions than other simulations are not restricted to the landing site region, and the mismatch with aeolian features in the region suggests that this prediction may be incorrect.

Dust devil (or convective vortex) activity as a function of season is predicted using mesoscale MarsWRF output and the Renno et al. (1998) theory that relates the amount of daytime convective vortex activity to sensible heat flux and boundary layer depth. Although modifications may be needed – for example, to account for the greater role of IR heating of the near-surface atmosphere on Mars and for the “observer effect” of ambient wind blowing more vortices over a stationary sensor per hour – the theory has generally matched Martian observations quite well to date. The DDA is predicted to peak between 12:00 and 14:00 in all seasons and to be greatest in mid-to-late summer, when daytime sensible heat fluxes are moderately high and PBL depths are at maximum, with the lowest DDA predicted in some sols around winter solstice, when daytime sensible heat fluxes are low in some sols and PBL depths are at their minimum. The magnitude of DDA is not predicted to vary greatly along the expected rover traverse. The DDA is predicted to be significantly greater than at MSL’s location in Gale crater; even at local (northern) winter solstice in the Jezero region, the predicted DDA barely falls below the predicted maximum value at MSL’s location in local (southern) summer. The maximum DDA is also far greater than that predicted for InSight, although the seasonal variation is greater (likely due to the higher latitude) hence the minimum DDA predicted for the Jezero region is lower. In short, peak vortex activity should generally be greater than at InSight and significantly greater than at MSL’s location. This may not translate to greater numbers of visible vortices (i.e. dust devils), which may depend on dust availability and other surface properties, but should result in larger and/or greater numbers of vortex pressure drops in Jezero than for the previous missions, at least when observer effects are accounted for.

## Supplementary Information

Below are the links to the electronic supplementary material.

Online Resource 1: High-resolution topography and the areal coverage of MRAMS grids 4-7, where grid 7 is the innermost nest. The dot indicates the landing site inside Jezero crater. (TIFF 614 kB)

Online Resource 2: Table showing the (1) diurnal, (2) semidiurnal, (3) terdiurnal, and (4) quaddiurnal pressure amplitudes, as a percentage of the total pressure perturbation from the daily mean, for four seasons ($\text{Ls}\sim0$ or $5^{\circ}$, $90^{\circ}$, $180^{\circ}$, and $270^{\circ}$), using the output plotted in Figs. 2b, 6b, 7b, and 8b, respectively. (DOCX 87 kB)

Online Resource 3: Animation of a portion of global MarsWRF showing winds (vectors) and wind speeds (shaded) over 10 sols at $\text{Ls}\sim5^{\circ}$. Also shown is topography (black contours). (MP4 17.8 MB)

Online Resource 4: Animation of the Isidis basin region of the global GEM-Mars model showing winds (vectors) over two sols at $\text{Ls}\sim5^{\circ}$. Also shown is topography (shaded). (MP4 3.7 MB)

Online Resource 5: Animation of mesoscale MarsWRF domain 5 winds (vectors) and wind speeds (shaded) over one sol at $\text{Ls}\sim5^{\circ}$. Also shown is topography (black contours). (MP4 35.9 MB)

Online Resource 6: Animation of mesoscale LMD winds (vectors) over one sol at $\text{Ls}\sim5^{\circ}$. Also shown is topography (shaded). (GIF 4.9 MB)

Online Resource 7: Animation of MRAMS domain 5 winds (vectors) and potential temperature (shaded) over one sol at $\text{Ls}\sim5^{\circ}$. Also shown is topography (black contours). (GIF 73.2 MB)

Online Resource 8: Animation of nested MarsWRF domain 2 winds (vectors) and wind speeds (shaded) over one sol at $\text{Ls}\sim90^{\circ}$. Also shown is topography (black contours). (MP4 34.1 MB)

Online Resource 9: As in Fig. 2 but now with the “global MarsWRF” result coming from domain 1 of the same nested MarsWRF simulation from which the “mesoscale MarsWRF” (domain 5) result is taken. (PDF 269 kB)

Online Resource 10: Animation of the Isidis basin region of the low-resolution Ames model showing winds (vectors) over one sols at $\text{Ls}\sim90^{\circ}$. Also shown is topography (shaded). (GIF 3.7 MB)
