# Supplementary figures and images for: Multi-model Meteorological and Aeolian Predictions for Mars 2020 and the Jezero Crater Region
Source: Space Sci Rev. 2021 Feb 8;217(1):20. doi: 10.1007/s11214-020-00788-2 (PMC7868679; doi:10.1007/s11214-020-00788-2)

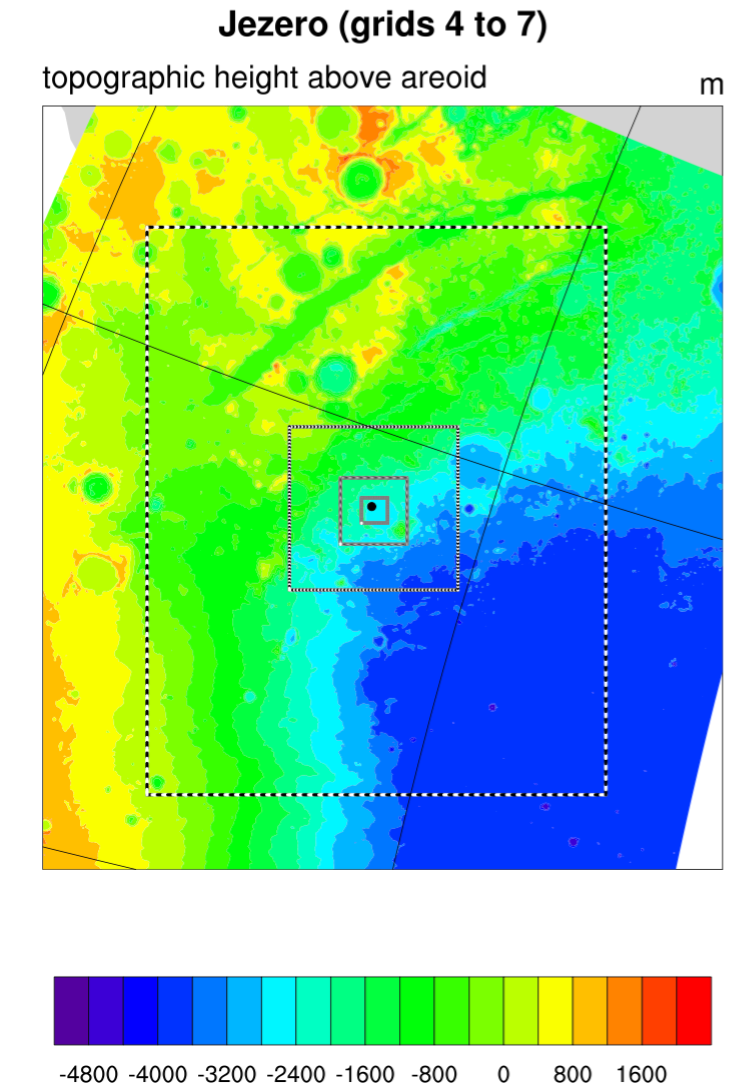

Supplement: Supplementary file 1 — Online Resource 1: High-resolution topography and the areal coverage of MRAMS grids 4-7, where grid 7 is the innermost nest. The dot indicates the landing site inside Jezero crater. (TIFF 614 kB) [file 11214_2020_788_MOESM1_ESM.tiff]

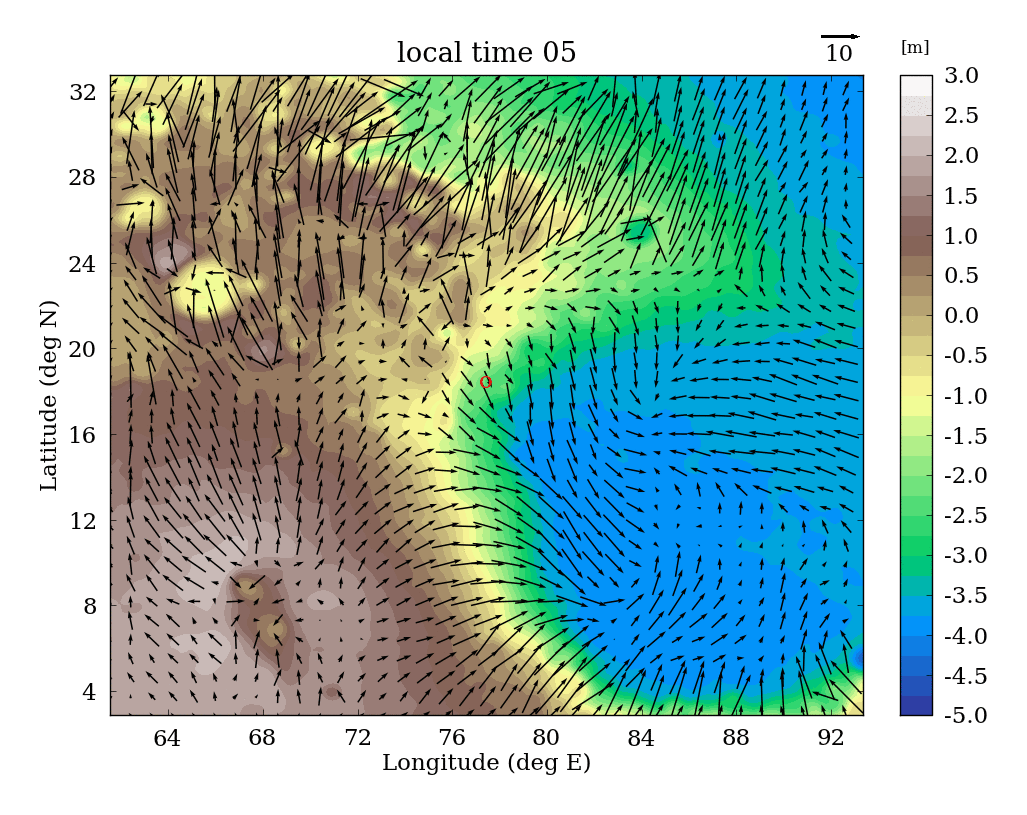

Supplement: Supplementary file 6 — Online Resource 6: Animation of mesoscale LMD winds (vectors) over one sol at $\text{Ls}\sim5^{\circ}$Ls∼5∘. Also shown is topography (shaded). (GIF 4.9 MB) [file 11214_2020_788_MOESM6_ESM.gif]

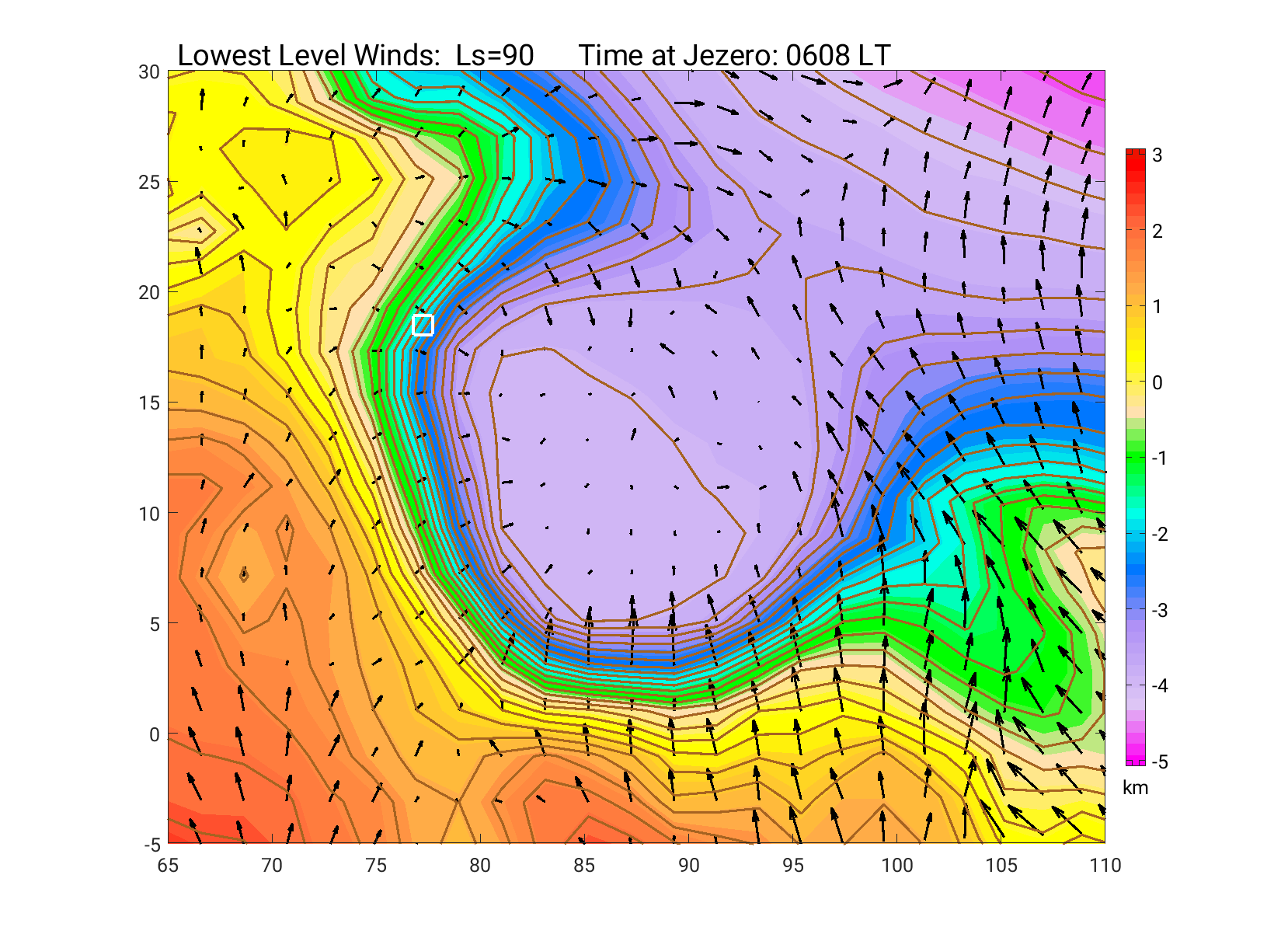

Supplement: Supplementary file 10 — Online Resource 10: Animation of the Isidis basin region of the low-resolution Ames model showing winds (vectors) over one sols at $\text{Ls}\sim90^{\circ}$Ls∼90∘. Also shown is topography (shaded). (GIF 3.7 MB) [file 11214_2020_788_MOESM10_ESM.gif]
