# Supplementary material for: Multi-model Meteorological and Aeolian Predictions for Mars 2020 and the Jezero Crater Region
Source: Space Sci Rev. 2021 Feb 8;217(1):20. doi: 10.1007/s11214-020-00788-2 (PMC7868679; doi:10.1007/s11214-020-00788-2)
Supplement: Supplementary file 2 — Online Resource 2: Table showing the (1) diurnal, (2) semidiurnal, (3) terdiurnal, and (4) quaddiurnal pressure amplitudes, as a percentage of the total pressure perturbation from the daily mean, for four seasons ($\text{Ls}\sim0$Ls∼0 or $5^{\circ}$5∘, $90^{\circ}$90∘, $180^{\circ}$180∘, and $270^{\circ}$270∘), using the output plotted in Figs. 2b, 6b, 7b, and 8b, respectively. (DOCX 87 kB) [file 11214_2020_788_MOESM2_ESM.docx]

|  |  | GEM-Mars | LMD (global) | Open  MARS | Ames (low res) | Mars  WRF (global) | LMD (meso  scale) | Ames (high res) | Mars  WRF (dom5) | MRAMS |
| --- | --- | --- | --- | --- | --- | --- | --- | --- | --- | --- |
| Ls=0° | 1 | 0.73 | 1.28 | 1.71 | 1.12 | 0.70 | 1.98 | 1.28 | 0.71 | 1.22 |
|  | 2 | 0.89 | 0.73 | 0.67 | 1.03 | 1.16 | 0.82 | 0.81 | 1.29 | 1.01 |
|  | 3 | 0.41 | 0.06 | 0.39 | 0.09 | 0.22 | 0.14 | 0.09 | 0.15 | 0.15 |
|  | 4 | 0.19 | 0.54 | 0.30 | 0.32 | 0.41 | 0.49 | 0.36 | 0.41 | 0.54 |
| Ls=90° | 1 | 1.00 | 0.66 | 0.61 | 1.02 | 0.55 | - | 0.85 | 0.34 | 0.71 |
|  | 2 | 1.43 | 1.05 | 0.22 | 0.67 | 0.45 | - | 0.37 | 0.45 | 0.90 |
|  | 3 | 0.19 | 0.09 | 0.34 | 0.21 | 0.15 | - | 0.01 | 0.18 | 0.21 |
|  | 4 | 0.09 | 0.27 | 0.16 | 0.27 | 0.24 | - | 0.01 | 0.27 | 0.35 |
| Ls=180° | 1 | 1.48 | 2.54 | 2.60 | 2.09 | 1.03 | - | 0.79 | 1.30 | 2.50 |
|  | 2 | 0.95 | 0.88 | 0.91 | 1.00 | 1.23 | - | 0.46 | 1.33 | 1.20 |
|  | 3 | 0.33 | 0.12 | 0.30 | 0.08 | 0.23 | - | 0.45 | 0.12 | 0.13 |
|  | 4 | 0.21 | 0.56 | 0.41 | 0.57 | 0.37 | - | 0.07 | 0.40 | 0.66 |
| Ls=270° | 1 | 1.03 | 1.32 | 2.03 | 1.94 | 0.31 | - | 1.48 | 0.63 | 1.77 |
|  | 2 | 1.18 | 1.18 | 1.11 | 1.46 | 2.01 | - | 0.32 | 2.09 | 1.30 |
|  | 3 | 0.19 | 0.17 | 0.26 | 0.02 | 0.26 | - | 0.14 | 0.26 | 0.18 |
|  | 4 | 0.20 | 0.35 | 0.32 | 0.28 | 0.40 | - | 0.05 | 0.43 | 0.37 |
