# Supplementary material for: Multi-model Meteorological and Aeolian Predictions for Mars 2020 and the Jezero Crater Region
Source: Space Sci Rev. 2021 Feb 8;217(1):20. doi: 10.1007/s11214-020-00788-2 (PMC7868679; doi:10.1007/s11214-020-00788-2)
Supplement: Supplementary file 9 — Online Resource 9: As in Fig. 2 but now with the “global MarsWRF” result coming from domain 1 of the same nested MarsWRF simulation from which the “mesoscale MarsWRF” (domain 5) result is taken. (PDF 269 kB) [file 11214_2020_788_MOESM9_ESM.pdf]

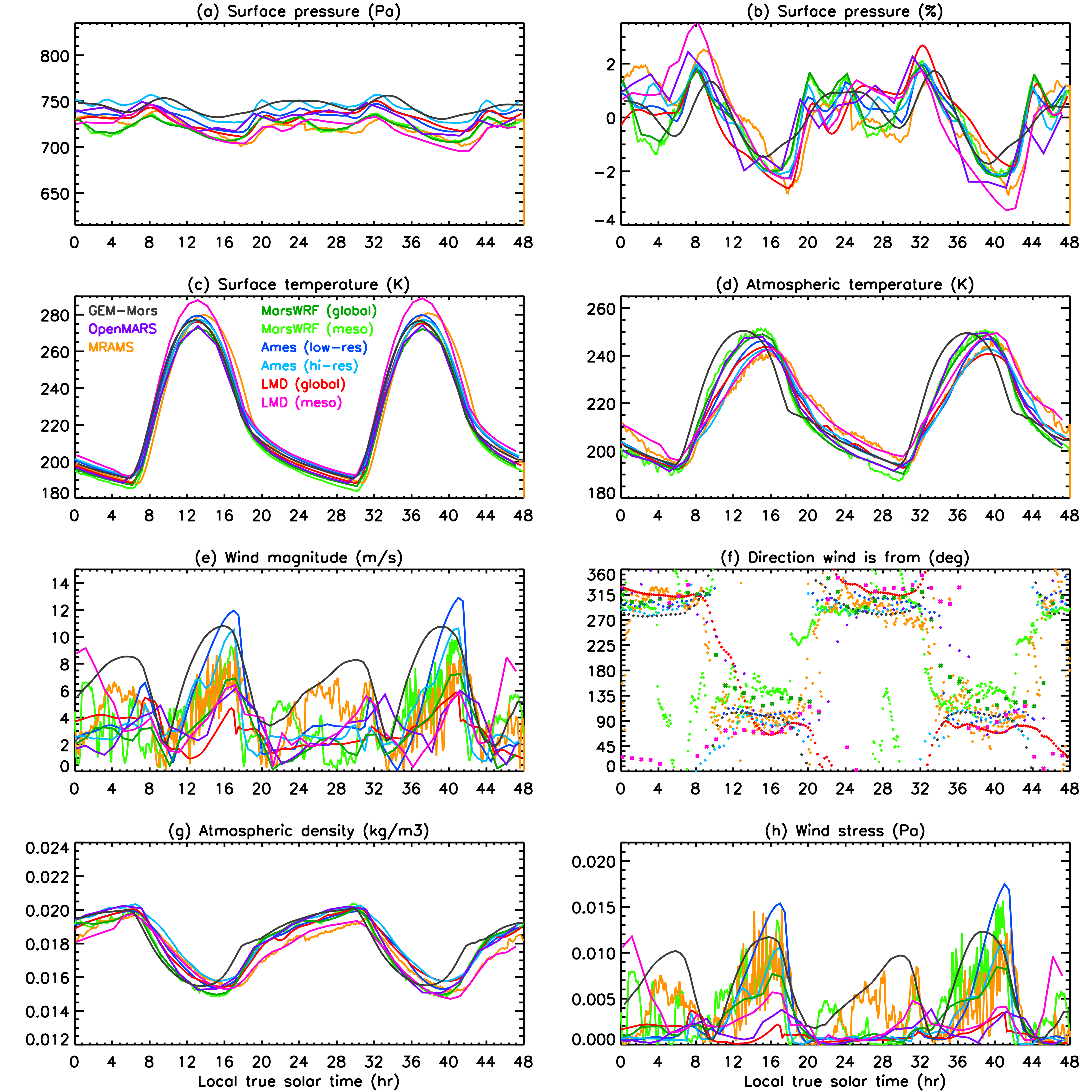

(a) Surface pressure (Pa)
(b) Surface pressure (%)
(c) Surface temperature (K)
(d) Atmospheric temperature (K)
(e) Wind magnitude (m/s)
(f) Direction wind is from (deg)
(g) Atmospheric density (kg/m3)
(h) Wind stress (Pa)
GEM-Mars
OpenMARS
MRAMS
MarsWRF (global)
MarsWRF (meso)
Ames (low-res)
Ames (hi-res)
LMD (global)
LMD (meso)
Local true solar time (hr)
